# Chemo-enzymatic total synthesis of the spirosorbicillinols

**DOI:** 10.1038/s42004-023-00996-1

**Published:** 2023-09-06

**Authors:** Tobias M. Milzarek, Tobias A. M. Gulder

**Affiliations:** 1 Chair of Technical Biochemistry, Technical University of Dresden, Bergstraße 66, 01069 Dresden, Germany; 2 Helmholtz Institute for Pharmaceutical Research Saarland (HIPS), Department of Natural Product Biotechnology, Helmholtz Centre for Infection Research (HZI), Saarland University, 66123 Saarbrücken, Germany; 3 Department of Pharmacy, Saarland University, 66123 Saarbrücken, Germany; 4 Present Address: Laboratory of Catalysis and Organic Synthesis, École Polytechnique Fédérale de Lausanne, EPFL, SB ISIC LCSO, 1015 Lausanne, Switzerland

**Keywords:** Natural product synthesis, Biocatalysis, Oxidoreductases, Natural product synthesis

## Abstract

The natural product class of the sorbicillinoids is composed of structurally diverse molecules with many strong, biomedically relevant biological activities. Owing to their complex structures, the synthesis of sorbicillinoids is a challenging task. Here we show the first total synthesis of the fungal sorbicillinoids spirosorbicillinols A–C. The convergent route comprises the chemo-enzymatic transformation of sorbicillin to the highly reactive sorbicillinol and the assembly of scytolide and isomers starting from shikimic and quinic acid analogs. The key step in the total synthesis is the fusion of both building blocks in a Diels-Alder cycloaddition leading to the straightforward formation of the characteristic sorbicillinoid bicyclo[2.2.2]octane backbone. This work provides unifying access to all natural spirosorbicillinols and unnatural diastereomers.

## Abstract (web summary)

Spirosorbicillinols A–C are fungal natural products that display various biological properties, but their total synthesis has yet to be accomplished. Here, the authors report the total synthesis of spirosorbicillinols A–C based on a chemo-enzymatic transformation of sorbicillin into sorbicillinol, which is fused to synthetically prepared scytolide and isomers by a Diels-Alder cycloaddition reaction.

## Introduction

**Fig. 1:**
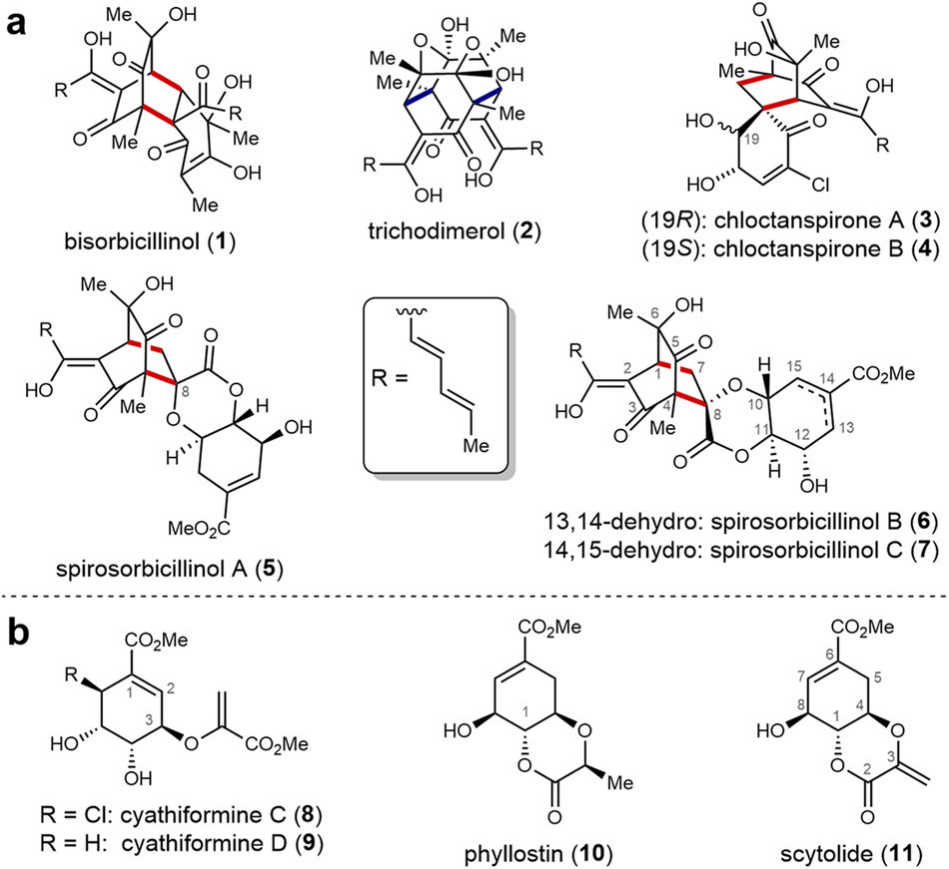
Natural product structures of this work. **a** Diverse sorbicillinoids based on Diels–Alder cycloaddition (red bonds) and Michael addition (blue bonds). **b** Fungal, shikimic-derived secondary metabolites^11–15^.

The sorbicillinoids are a class of structurally highly diverse natural products with multiple biological activities^1^. Among the best-known representatives are bisorbicillinol (**1**) and trichodimerol (**2**) derived of Diels-Alder cycloaddition (red bonds) or Michael addition (blue bonds) reactions, respectively, of two sorbicillinol units (Fig. 1a). In terms of biological activity, **1** acts as a DPPH radical scavenger, whereas **2** inhibits the production of the inflammatory mediator tumor necrosis factor (TNF-α) by targeting cyclooxygenase-2^2,3^. Bio-synthetically and total synthetically, all such dimeric sorbicillinoids can be deduced from oxidative dearomatization of sorbicillin to the reactive intermediate sorbicillinol, either by the natural oxidoreductase SorbC^4–7^ or by oxidizing reagents, such as lead tetraacetate or (bis(trifluoroacetoxy)iodo)benzene^8–10^.

In addition to dimeric sorbicillinoids, hybrid derivatives, which consist of one sorbicillin moiety and an additional molecular building block, are also present in nature. Examples are the chloctanspirones A (**3**) and B (**4**) and the spirosorbicillinols A–C (**5**–**7**), all including a spiro-cyclic quaternary carbon at position C-8 (Fig. 1a). The chlorinated sorbicillinoids **3** and **4** possess cytotoxic effects against leukemic (HL-60, **3**: IC_50_ = 9.20 μM, **4**: IC_50_ = 37.8 μM) and human adenocarcinoma cells (A-549, 3: IC_50_ = 39.7 μM)^11^. In analogy to bisorbicillinol (**1**), the spirosorbicillinols A–C (**5**–**7**) show weak DPPH radical scavenging activities^12^. From a retrosynthetic perspective, spirosorbicillinols are assembled from a shikimic acid-derived secondary metabolite acting as a dienophile in the Diels–Alder cycloaddition with sorbicillinol. Fungal shikimate metabolites are a natural product family with diverse functionalities, such as a typical allylic methyl ester and a vicinal triol. Examples of this structural class are the cyathiformines (**8**, **9**)^15^, phyllostin (**10**)^14^, and scytolide (**11**)^14,15^ (Fig. 1b). These compounds have an additional methyl acrylate or propanoate group, respectively, which in some congeners forms a six-membered lactone with the alcohol at position C-1. Scytolide (**11**) is a key building block for the chemo-enzymatic synthesis of spirosorbicillinols A (**5**) and B (**6**), since it represents the dienophile for the Diels–Alder cycloaddition with sorbicillinol^5,6,16,17^. We thus set out to develop chemical routes to access scytolide (**11**) and various isomers to enable their straightforward conversion into the desired products spirosorbicillinols A–C (**5**–**7**).

## Results and discussion

### Synthesis of scytolides

**Fig. 2:**
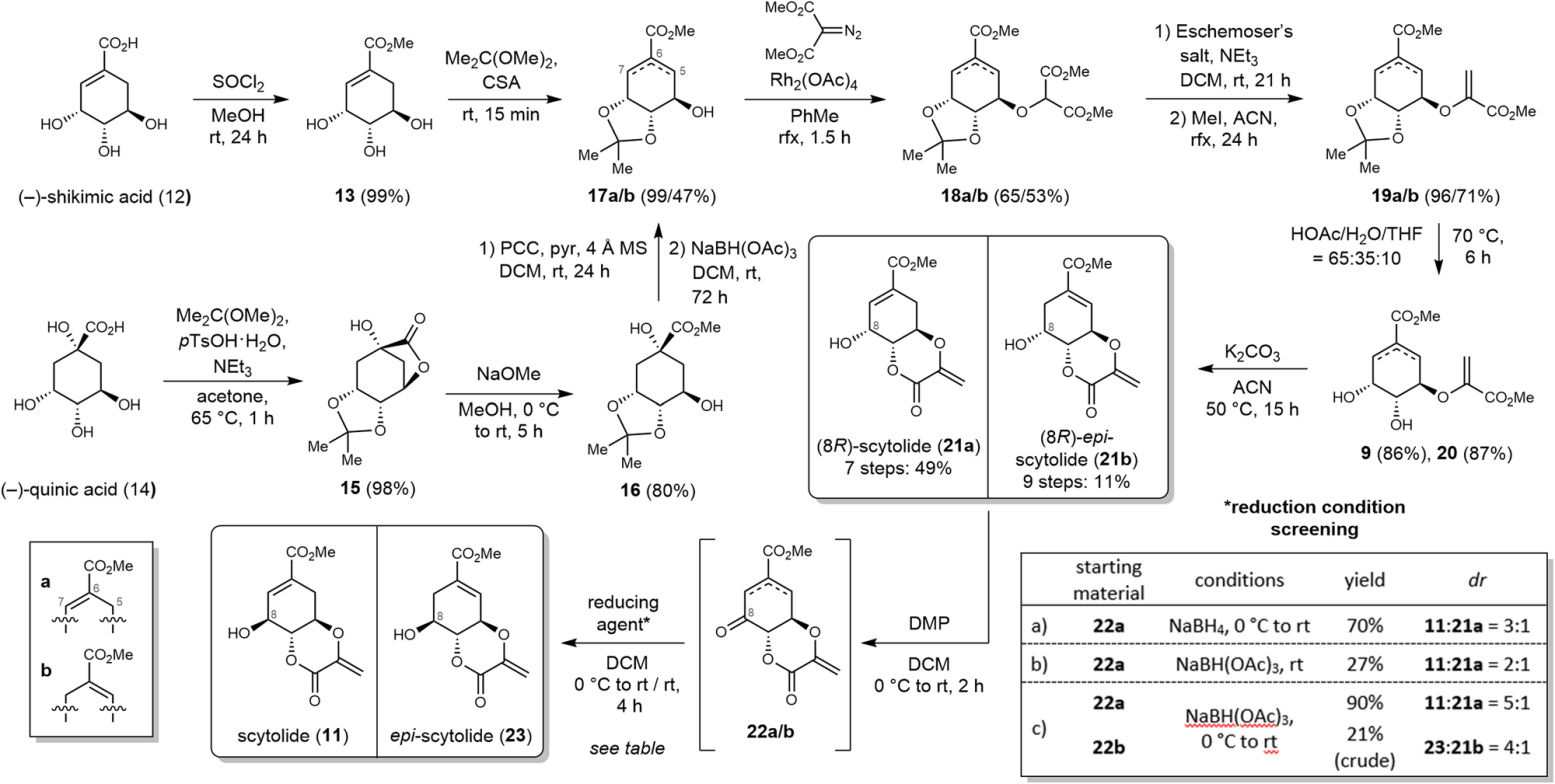
Synthetic routes for the formation of scytolide and multiple isomers. Isolated yields are given. ACN acetonitrile, CSA camphorsulfonic acid, PCC pyridinium chlorochromate, DMP Dess-Martin periodinane^16–18^.

Due to identical stereochemistry, the enantioselective assembly of scytolide (**11**) and further isomers can be established by starting from chiral-pool biogenic precursors (–)-shikimic acid (**12**) or (–)-quinic acid (**14**) (Fig. 2). In the first steps, both natural building blocks were protected by esterification and cyclic acetal formation^18,19^. (–)-Shikimic acid (**12**) was converted into the protected species **17a** (98% yield over 2 steps) with thionyl chloride in methanol and acid-catalyzed cyclization with 2,2-dimethoxypropane. (–)-Quinic acid (**14**) was treated analogously with 2,2-dimethoxypropane leading to the lactonized isopropylidene acetal **15** in 98% yield. Ring opening of the lactone was achieved by the addition of sodium methanolate (80% yield). Subsequent oxidative β-elimination^20^ using pyridinium chlorochromate (PCC) and pyridine led to a keto intermediate, which was selectively reduced with sodium triacetoxyborohydride (47% yield over 2 steps) to compound **17b**, the C-5/C-6-double-bond isomer of **17a**. The attachment and lactonization of the methyl acrylate moiety were performed in analogy to previous work by Chouinard and Bartlett on shikimate metabolite synthesis^21,22^. The condensation of the isopropylidene-protected shikimic acid methyl esters **17a**/**b** with dimethyl diazomalonate was catalyzed by rhodium acetate (yields—**18a**: 65%, **18b**: 53%)^23^. The obtained malonates **18a**/**b** were methenylated using Eschenmoser’s salt, followed by alkylation with iodomethane and Hofmann-type elimination (yields over 2 steps—**19a**: 96%, **19b**: 71%). Before final cyclization, the isopropylidene protecting group of compounds **19a/b** was removed under acidic conditions (acetic acid/water/tetrahydrofuran, yields—**9**: 86%, **20**: 87%). Subsequently, the synthesized cyathiformine D (**9**)^24^ and its double bond regioisomer **20** were lactonized with potassium carbonate (yields—**21a/b**: 93%). Thus, as essential intermediates, two diastereomeric (8*R*)-scytolide isomers were prepared over seven (**21a**) or nine (**21b**) synthetic steps, respectively, in total yields of 49% and 11%. The yield-determining step in both cases was the rhodium-catalyzed condensation reaction (53–65%), in addition to the PCC-promoted β-elimination (60%) for (8*R*)-*epi*-scytolide (**21b**). For the inversion of the stereocenter at C-8, compounds **21a**/**b** were oxidized with Dess–Martin periodinane (DMP) to give the corresponding ketones **22a**/**b**. Analysis by high-performance liquid chromatography indicated a quantitative conversion in each case. Due to the observed instability of the oxidized species **22a**/**b**, the subsequent reductions were carried out directly with the crude intermediates. The use of sodium borohydride^25^ as a reducing agent at 0 °C provided a diastereomeric mixture of **11** and **21a** in a 3:1 ratio with an overall yield of 70% (Fig. 2, Table, entry a). The milder sodium triacetoxyborohydride showed lower conversion (27%, **11**:**21a** = 2:1) at room temperature (b), whereas at 0 °C (c), the yield (90%) and the diastereomeric ratio (**11**:**21a** = 5:1) were improved. Under the same conditions (c), crude *epi*-scytolide (**23**) was prepared via ketone **22b** in 21% yield (**23**:**21b** = 4:1). Due to the small amount isolated and the sensitivity to polymerization, the crude product **23** was used directly without further purification in subsequent reactions (see below). In summary, four different scytolide isomers **11**, **21a**/**b**, **23** were produced over 7–11 synthetic steps in yields between 2 and 49%. To the best of our knowledge, the natural product scytolide (**11**) was synthesized for the first time, in nine steps in a total yield of 35%.

### Synthesis of spirosorbicillinols

**Fig. 3:**
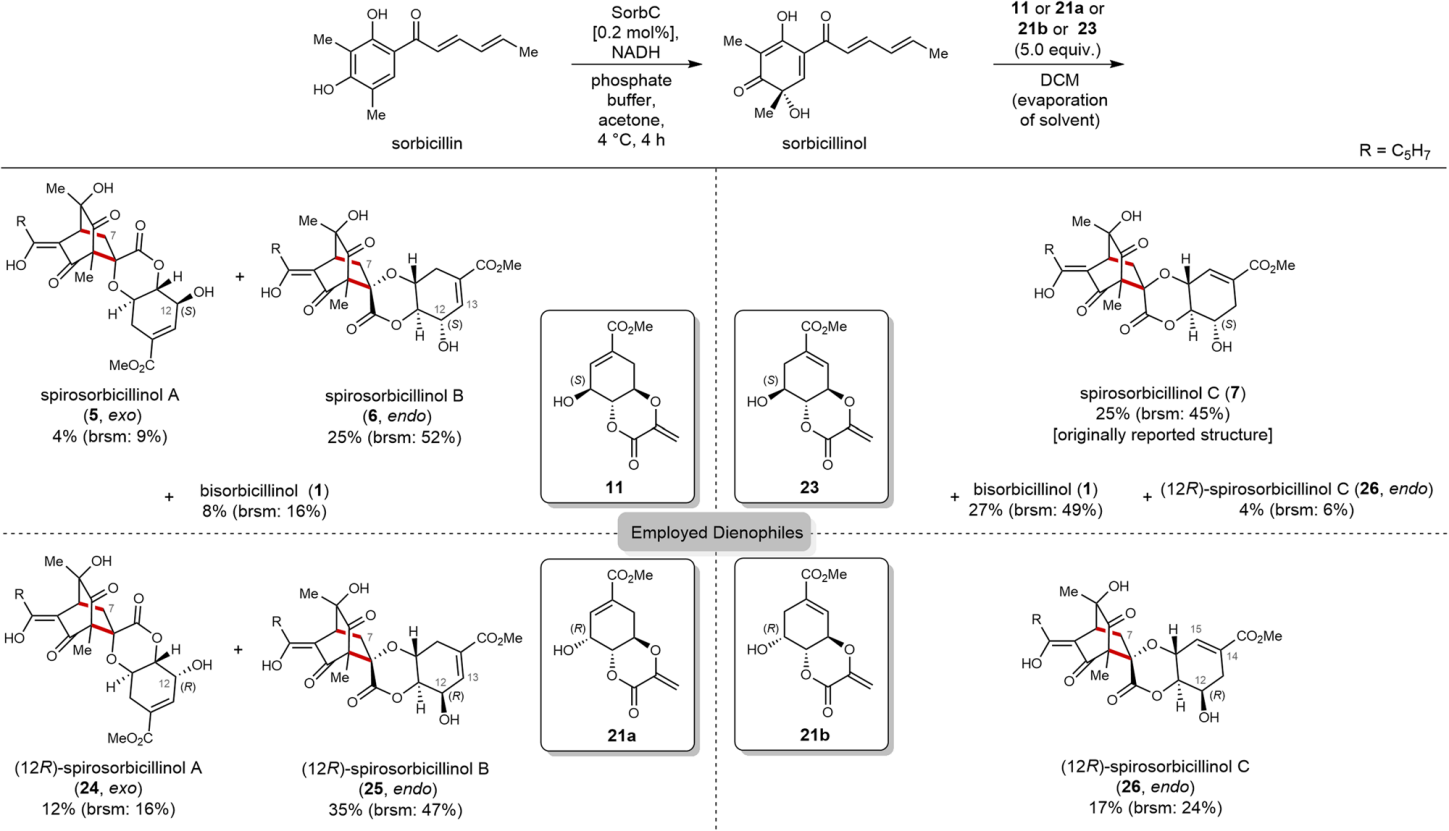
Total synthesis of spirosorbicillinol A–C (5–7) and unnatural 12*R*-analogs 24–26. Isolated yields are given. brsm based on re-isolated starting material.

Having scytolide (**11**) and *epi*-scytolide (**23**) in hands, their use as dienophiles in Diels–Alder reactions with chemo-enzymatically prepared sorbicillinol^5,6^ (enophile) gave direct access to the entire class of spirosorbicillinols (Fig. 3). Spirosorbicillinol A (**5**) and B (**6**) were prepared by a single reaction using the same dienophile **11**. During Diels–Alder cycloaddition, the preferred *endo*-derivative **6** (25%, based on re-isolated starting material (brsm): 52%) was obtained in higher yields compared to the *exo*-product **5** (4%, brsm: 9%). Using the double-bond isomer *epi*-scytolide **23** resulted in the Diels–Alder reaction product similar to spirosorbicillinol C (**7**) in a yield of 25% (brsm: 45%). Interestingly, in all previous chemo-enzymatic syntheses of bisorbicillinoids originating from Diels–Alder reactions, only the *endo* products were observed^5,6,16,17^. Presumably due to the very large dienophiles, *exo*-addition products were produced and isolated for the first time in this work, although in comparatively low yields. Aside from the desired target structures, we also observed formation of bisorbicillinol (**1**) as product, resulting from the not preventable dimerization of sorbicillinol (in the synthesis of **5**/**6**: 8% of compound **1**, **7**: 27% of **1**). Nuclear magnetic resonance (NMR) analysis of the prepared spirosorbicillinols (see Supplementary Figs. S23–S51) showed that compounds **5** and **6** indeed correspond to the natural products isolated from the fungus *Trichoderma* sp. USF-4860^14,15^, hence concluding their first and stereoselective total synthesis. To our surprise, the analytical data of synthesized compound **7** were different from those published for the natural product, spirosorbicillinol C (**7**)^12^. In particular, the optical rotation (reported: [*α*]_D_ = +484.2; measured: [α]_D_ = +109.1) and the indicative ^1^H NMR chemical shifts at position 7 (reported: *δ*_H_ = 2.99, 2.34 ppm, measured: *δ*_H_ = 3.15, 2.22 ppm) showed considerable differences. Having the C-8 epimers **21a/b** of scytolide (**11**) and *epi*-scytolide (**23**) in hands, we therefore next explored if these differences might be explained by a change of configuration at C-12 in the final product. Reaction of **21b** with sorbicillinol delivered the desired (12*R*)-spirosorbicillinol C (**26**) in 17% yield (brsm: 24%). Interestingly, the reported optical rotation of natural **7** corresponded almost perfectly to the synthesized C-12-diastereomer **26** ([*α*]_D_ = +455.6). However, the NMR shifts of the scytolide backbone in compound **26** (see Table S7, positions 12–15) did also not match with the literature values of **7**. The analytical data recorded for **7** strongly indicate that we indeed produced the reported *endo* structure. This is also in agreement with the isolated yield of 25%, which is in the typical range of *endo* product formation in chemo-enzymatic sorbicillinoid syntheses^5,6,16,17^. The nuclear Overhauser effect spectroscopy NMR data of synthetic **7** (Fig. S30) showed similar interactions compared to the reported spirosorbicillinol C (**7**)^12^. Due to the lack of formation of the putative *exo* product analog of **7** in our synthesis, we can unfortunately not unambiguously determine the structure of natural **7**. Analogously to the spirosorbicillinol C derivative **26**, the respective (12*R*)-diastereomers of spirosorbicillinol A and B, **24** and **25**, were synthesized in yields of 12% (brsm: 16%) and 35% (brsm: 47%) using (8*R*)-scytolide (**21a**). Comparison of the analytical data of these two epimers to their natural product counterparts showed no match, hence further corroborating the stereocenter at position 12 to be correct.

## Conclusions

In summary, we herein systematically combined the enzymatic synthesis of sorbicillinol using the heterologously expressed monooxygenase SorbC with the chemical synthesis of various scytolide analogs, providing synthetic access to the natural product family of the spirosorbicillinols and several unnatural diastereomers. Besides the first total syntheses of the spirosorbicillinols and unnatural diastereomers, this work also presents an efficient ten-step synthesis of the shikimic-derived natural product scytolide (**11**) along with a range of different double bond/C-8 stereoisomers. Overall, the developed syntheses of the spirosorbicillinols highlight the great potential of chemo-enzymatic approaches to the streamlined formation of structurally complex sorbicillinoids.

## Methods

### General methods

For instrumentation and material, see Supplementary Information—Experimental procedures, Supplementary Figures, and NMR spectra of new compounds (see Tables S1–S7, Figs. S1–S51).

### Supplementary information

Supplementary Information

## Data Availability

All data generated and analyzed during this study are included in this article, its Supplementary Information, and also available from the authors upon reasonable request.
