# Supplementary material for: Chemo-enzymatic total synthesis of the spirosorbicillinols
Source: Commun Chem. 2023 Sep 6;6:187. doi: 10.1038/s42004-023-00996-1 (PMC10482909; doi:10.1038/s42004-023-00996-1)
Supplement: Supplementary file 1 — Supplementary Information [file 42004_2023_996_MOESM1_ESM.pdf]

# Chemo-Enzymatic Total Synthesis of the Spirosorbicillinols

Tobias M. Milzarek,[1] and Tobias A. M. Gulder[1-3*]

[1] Chair of Technical Biochemistry, Technical University of Dresden, Bergstraße 66, 01062 Dresden, Germany. E-mail: tobias.gulder@tu-dresden.de

[2] Helmholtz Institute for Pharmaceutical Research Saarland (HIPS), Department of Natural Product Biotechnology, Helmholtz Centre for Infection Research (HZI), Saarland University, 66123 Saarbrücken, Germany.

[3] Department of Pharmacy, Saarland University, 66123 Saarbrücken, Germany.

## Supplementary Information

## Contents

**1. General Methods………… S2**

**2. Chemical Procedures………… S4**

2.1 Synthesis of Scytolide (**11**) and (8*R*)-Scytolide (**21a**)………… S4

2.2 Synthesis of *epi*-Scytolide (**23**) and (8*R*)-*epi*-Scytolide (**21b**)………… S9

2.3 Chemo-enzymatic Total Synthesis of the Spirosorbicillinols………… S14

2.4 NMR Comparison with Literature………… S22

**3. NMR Data………… S25**

**4. Supplementary References………… S56**

# Supplementary Methods

## 1. General Methods

**Reagents:** Solvents for HPLC and MS analysis, such as acetonitrile and methanol, were purchased from Fisher Scientific and VWR in a purity of over 99% (HPLC-grade). Water was purified using a TKA GenPure water treatment system and deionized. Dry solvents such as diethyl ether, dichloromethane, methanol, tetrahydrofuran and toluene for procedures under inert atmosphere were prepared by distillation and drying over molecular sieves (3 Å or 4 Å). Commercial materials and other solvents were purchased at the highest commercial quality from the providers Acros Organics, Alfa Aesar, Carbolution, Carl Roth, Merck, Sigma Aldrich, VWR, TCI Chemicals and Thermo Fisher Scientific. Air- and moisture-sensitive reactions were performed under argon atmosphere using a Schlenk line. Before application, the flasks were repeatedly evacuated (external heating) and refilled with argon.

**NMR:** $^{1}H$ and $^{13}C$ Nuclear Magnetic Resonance Spectra (NMR) were recorded on Bruker AV-300 and AV-600 spectrometers at 298 K. The chemical shifts are given in δ-values (ppm) and are calibrated on the residual peak of the deuterated solvent ($CDCl_3$: $\delta_H$ = 7.26 ppm, $\delta_C$ = 77.0 ppm; acetone-$d_6$: $\delta_H$ = 2.05 ppm, $\delta_C$ = 29.8 ppm). The coupling constants *J* are given in Hertz [Hz]. Following abbreviations were used for the allocation of signal multiplicities: bs – broad signal, s – singlet, d – doublet, dd – doublet of doublets, ddd – doublet of doublet of doublets, ddt – doublet of doublets of triplets, t – triplet, td – triplet of doublets, m – multiplet.

**MS:** Elektrospray-Ionisation Mass spectra (ESI-MS) were recorded on an Advion expressionL CMS system using a single-quadrupole mass analyzer, a Peak Scientific N118LA nitrogen generator, an Edwards RV12 high vacuum pump and a Jasco PU-1580 Intelligent HPLC Pump, or a LCQ Fleet ion trap system (Thermo Scientific), which was combined with an UltiMate 300 HPLC system. For high resolution mass spectrometry (HRMS) an Agilent mass spectrometer 6538 with atmospheric pressure chemical ionization (APCI), high resolution Q-TOF mass analyzer and microchannel plate detector was used.

**Chromatography:** Thin-layer chromatography (TLC) was performed on precoated plates of silica gel F254 (Merck) with UV detection at 254 and 365 nm. Column chromatography was performed on silica gel 60 Geduran® Si 60 (40–60 µm) (Merck). High Performance Liquid Chromatograms (HPLC) were recorded on a computer-controlled Jasco system including a UV-1575 Intelligent UV/VIS Detector, DG-2080-53 3-Line Degaser, two PU-1580 Intelligent HPLC Pumps, AS-1550 Intelligent Sampler, HG-1580-32 Dynamic Mixer. A Eurospher II 100-3 C18 A (150 × 4.6 mm) column with integrated precolumn manufactured by Knauer was used. The eluent system consisted of A = $H_2O$ + 0.05% TFA, B = MeCN + 0.05% TFA. The analytical method used the following elution gradient: 0–1 min 5% B, 1–15 min to 95% B, 15–18 min 95% B, 18–18.5 min to 5% B, 18.5–20 min 5% B with a flowrate of 1 mL/min. For medium pressure liquid chromatography (MPLC) the Reveleris® X2 MPLC system (Grace) was used together with

Reverleris® Reverse Phase (RP) C18 columns (Grace) using UV-detection at 220 nm, 254 nm, and 280 nm. Isolation of the chemo-enzymatically produced compounds was carried out by semi-preparative HPLC controlled by a Jasco HPLC system consisting of an UV-1575 Intelligent UV/VIS Detector, two PU-2068 Intelligent prep. Pumps, a MIKA 1000 Dynamic Mixing Chamber (1000 µL Portmann Instruments AG Biel-Benken), a LC-NetII/ ADC, and a Rheodyne injection valve. The system was controlled by the Galaxie-Software and the eluent system consisted of: A = $H_2O$ + 0.05% TFA and B = ACN + 0.05% TFA. A Eurosphere II 100-5 C18 A (250 x 16 mm) column with precolumn (30 x 16 mm) provided by Knauer was used as the stationary phase. General HPLC condition: gradient: 0–1 min 95% $H_2O$ + 0.05% TFA (A) / 5% acetonitrile + 0.05% TFA (B), 1–55 min 5% A / 95% B, 55–56 min 5% A / 95% B, 56–58 min 95% A / 5% B, 58–60 min 95% A / 5% B, flow rate: 10 mL/min, running time: 60 min.

**Optical rotataion:** The specific rotation was measured with a PerkinElmer Model 341 LLC Polarimeter at 20 °C. The concentration for the specific rotation measurements is given in 10 mg/mL.

**Recombinant protein production and purification:** The bacterial strain *Escherichia coli* Δmtn was used as host to produce the monooxygenase SorbC. *E. coli* were grown in 2xYT media using 3 L Fernbach flasks. Glycerol stocks were used to inoculate starting cultures for production. The bacteria were grown at 37 °C in LB medium containing 50 µg/mL kanamycin sulfate overnight as preculture. The starting culture was further used to inoculate 2xYT (1:100) medium supplemented with the same concentration of antibiotic. When an $OD_{600}$ = 0.5 was reached, the medium was cooled down and protein production was induced by adding 0.1 mM Isopropyl β-D-thiogalactoside (IPTG), followed by an overnight incubation at 16 °C with shaking (180 rpm). The cells were harvested by centrifugation and re-suspended in lysis buffer (50 mM $NaH_2PO_4$, 300 mM NaCl, 15 mM imidazole, 10% glycerol, pH 7.5). After sonification on ice and subsequent centrifugation (12000 rpm, 4 °C, 30 min), the supernatant was incubated with PureCube Ni-NTA Agarose (Cube Biotech) with shaking (100 rpm) for at least one hour. The suspension was applied to an empty 50 mL BioRad column, which was washed with one bed volume of lysis buffer. The protein was eluted with a higher concentrated imidazole buffer (50 mM $NaH_2PO_4$, 300 mM NaCl, 250 mM imidazole, 10% glycerol, pH 7.5). PD-10 columns (GE Healthcare) were used to exchange the buffer to the storage solution (50 mM $NaH_2PO_4$, 300 mM NaCl, 10% glycerol, pH 7.5). The protein concentration was determined photometrically using the Nanophotometer 330 (Implen) at 280 nm using the extinction coefficient of SorbC ε(280 nm) = 52420 $M^{-1}$ $cm^{-1}$ and a molecular weight of 61582.52 Da. The enzymatic assays were performed in phosphate buffer (50 mM, pH 8.0).

## 2. Chemical Procedures

## 2.1 Synthesis of Scytolide (11) and (8*R*)-Scytolide (21a)

### Methyl (–)-shikimate (13)

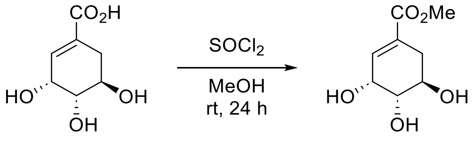


(–)-Shikimic acid (**12**, 2.00 g, 11.5 mmol, 1.0 eq.) was dissolved in dry methanol (34.5 mL, 3.0 mL/mmol). Thionyl chloride (1.50 mL, 2.46 g, 20.7 mmol, 1.8 eq.) was added dropwise and the reaction mixture was stirred for 24 h at room temperature. The reaction mixture was concentrated in vacuo to give the desired methyl ester **13** (2.16 g, 11.5 mmol, 99%) as a white solid. Analytical data were in agreement the literature values.[1]

**ORD**: $[\alpha]_D$ = –95.3 (c = 0.36 in MeOH). **TLC**: $R_f$ (*n*-pentane/EtOAc = 1:1) = 0.36. **$^1$H-NMR** (300 MHz, acetone-$d_6$): δ = 6.77–6.74 (m, 1 H, *H*C=C), 4.76–4.70 (m, 1 H, C*H*OH), 4.14 (t, *J* = 6.1 Hz, 1 H, C*H*OH), 3.97 (td, *J* = 6.1, 4.4 Hz, 1 H, C*H*OH), 3.73 (s, 3 H, $CO_2CH_3$), 2.55 (ddt, *J* = 17.4, 4.2, 1.5 Hz, 1 H, HC=C$CH_2$), 2.30 (ddt, *J* = 17.4, 6.2, 1.5 Hz, 1 H, HC=C$CH_2$). **$^1$H-NMR** (300 MHz, DMSO-$d_6$): δ = 6.61 (d, *J* = 2.5 Hz, 1 H, *H*C=C), 4.46 (bs, 3 H, O*H*), 4.22 (bs, 1 H, C*H*OH), 3.86 (dt, *J* = 6.0, 4.1 Hz, 1 H, C*H*OH), 3.66 (s, 3 H, $CO_2CH_3$), 3.57 (dd, *J* = 5.8, 4.2 Hz, 1 H, C*H*OH), 2.42 (ddt, *J* = 18.2, 4.7, 2.4 Hz, 1 H, HC=C$CH_2$), 2.05 (ddt, *J* = 18.0, 3.6, 1.7 Hz, 1 H, HC=C$CH_2$). **MS** (ESI+): *m/z* = 189.1 $[M+H]^+$.

### Methyl 5-*O*-Acetyl-3,4-*O*-isopropylidene-(–)-shikimat (17a)

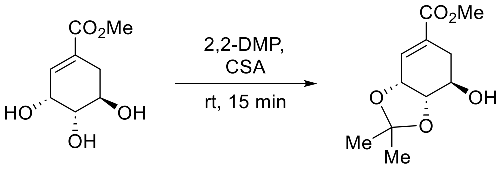


The protection was conducted according to a published procedure.[1] Methyl (–)-shikimate (**13**, 4.30 g, 22.9 mmol, 1.0 eq.) and camphorsulfonic acid (160 mg, cat.) were dissolved in 2,2-dimethoxypropane. After 15 min of stirring at room temperature, the reaction mixture was diluted with saturated $NaHCO_3$ solution, followed by extraction with diethyl ether. The combined organic layers were washed with brine, dried, filtered and concentrated in vacuo. The obtained residue was purified by column chromatography (*n*-pentane/EtOAc = 1:1) to give the desired product **17a** as a colorless oil (5.18 g, 22.7 mmol, 99%). Analytical data were in agreement with literature values.[1]

**ORD**: $[\alpha]_D$ = –80.8 (c = 1.04 in $CHCl_3$). **TLC**: $R_f$ (*n*-pentane/EtOAc = 1:1) = 0.46. **$^1$H-NMR** (300 MHz, $CDCl_3$): δ = 6.94–6.90 (m, 1 H, *H*C=C), 4.80–4.69 (m, 1 H, C*H*O), 4.10 (dt, *J* = 7.5, 5.9 Hz, 1 H, C*H*O), 3.90 (td, *J* = 8.0, 4.6 Hz, 1 H, C*H*O), 3.77 (s, 3 H, $CO_2CH_3$), 2.80 (ddt, *J* = 17.3, 4.8, 1.1 Hz, 1 H, HC=C$CH_2$), 2.24 (ddt, *J* = 17.5, 8.2, 1.9 Hz, 1 H, HC=C$CH_2$), 1.45 (s, 3 H, C$CH_3$), 1.40 (s, 3 H, C$CH_3$). **MS** (ESI+): *m/z* = 251.5 $[M+Na]^+$.

**Dimethyl 2-(((3a*S*,4*R*,7a*R*)-6-(methoxycarbonyl)-2,2-dimethyl-3a,4,5,7a-tetrahydro-benzo[*d*][1,3]dioxol-4-yl)oxy)malonate (18a)**

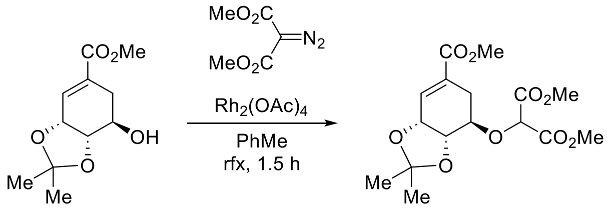


The reaction was conducted according to a published procedure.[2] The protected shikimate **17a** (4.70 g, 20.6 mmol, 1.0 eq.) and freshly prepared dimethyl diazomalonate (3.91 g, 24.7 mmol, 1.2 eq.) were dissolved in dry toluene (125 mL, 6.0 mL/mmol). After addition of rhodium(II) acetate (91.0 mg, 0.21 mmol, 1.0 mol%), the reaction mixture was stirred for 1.5 h under refluxing conditions. The mixture was filtered and concentrated under reduced pressure. The obtained residue was purified by MPLC (gradient, 5–95% ACN in 25 min) to give the desired product **18a** as a colorless oil (4.82 g, 13.5 mmol, 65%). Analytical data were in agreement with literature values.[2]

**ORD**: $[\alpha]_D$ = –47.9 (c = 0.97 in $CHCl_3$).**$^1$H-NMR** (300 MHz, $CDCl_3$): δ = 6.94–6.90 (m, 1 H, *H*C=C), 4.87 (s, 1 H, C*H*($CO_2Me)_2$), 4.73–4.64 (m, 1 H, C*H*O), 4.21 (dd, *J* = 7.4, 6.5 Hz, 1 H, C*H*O), 3.75 (s, 3 H, $CO_2CH_3$), 3.73 (s, 3 H, $CO_2CH_3$), 3.70 (s, 3 H, $CO_2CH_3$), 3.69–3.61 (m, 1 H, C*H*O), 2.81 (dd, *J* = 17.5, 4.7 Hz, 1 H, HC=C$CH_2$), 2.31 (ddt, *J* = 17.5, 8.5, 1.9 Hz, 1 H, HC=C$CH_2$), 1.36 (s, 3 H, $CH_3$), 1.31 (s, 3 H, C$CH_3$). **MS** (ESI+): *m/z* = 739.4 $[2M+Na]^+$.

**Dimethyl 2-((dimethylamino)methyl)-2-(((3a*S*,4*R*,7a*R*)-6-(methoxycarbonyl)-2,2-dimethyl-3a,4,5,7a-tetrahydrobenzo[*d*][1,3]dioxol-4-yl)oxy)malonate**

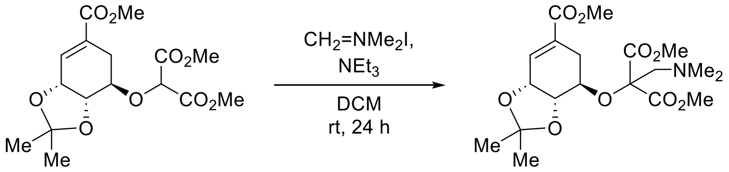


The reaction was conducted according to a published procedure.[2] The malonate **18a** (4.80 g, 13.4 mmol, 1.0 eq.) was dissolved in dry DCM (87.0 mL, 6.5 mL/mmol). After addition of triethylamine (1.87 mL, 1.36 g, 13.4 mmol, 1.0 eq.) and Eschenmoser's salt (2.97 g, 16.1 mmol, 1.2 eq.), the reaction mixture was stirred over night at room temperature. After completion, the reaction mixture was treated with water and extracted with DCM (3x). The combined organic layers were washed with 10% $Na_2CO_3$ solution, brine, dried, filtered and concentrated in vacuo to give the Eschenmoser adduct (5.56 g, 13.4 mmol, 99%) as colorless oil. Analytical data were in agreement with literature values.[2]

**ORD**: $[\alpha]_D$ = +5.0 (c = 0.71 in $CHCl_3$). **$^1$H-NMR** (300 MHz, $CDCl_3$): δ = 6.84–6.76 (m, 1 H, *H*C=C), 4.74–4.65 (m, 1 H, C*H*O), 4.54–4.44 (m, 1 H, C*H*O), 3.794 (s, 3 H, $CO_2CH_3$), 3.789 (s, 3 H, $CO_2CH_3$), 3.75 (s, 3 H, $CO_2CH_3$), 2.96–2.78 (m, 1 H, C*H*O), 2.64–2.41 (m, 2 H, HC=C$CH_2$), 2.23 (s, 6 H, $N(CH_3)_2$), 1.37 (s, 3 H, C$CH_3$), 1.32 (s, 3 H, C$CH_3$). **MS** (ESI+): *m/z* = 416.2 $[M+H]^+$.

**Methyl (3a*R*,7*R*,7a*S*)-7-((3-methoxy-3-oxoprop-1-en-2-yl)oxy)-2,2-dimethyl-3a,6,7,7a-tetrahydrobenzo[*d*][1,3]dioxole-5-carboxylate (19a)**

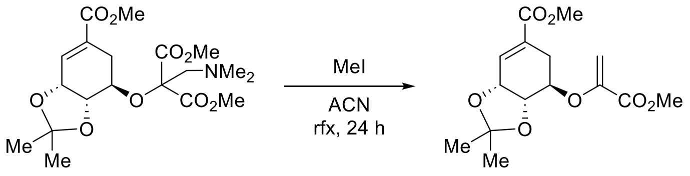


**19a**

The reaction was conducted according to a published procedure.[2] The Mannich base (260 mg, 0.63 mmol, 1.0 eq.) was dissolved in acetonitrile (2.20 mL, 3.5 mL/mmol) and iodomethane (390 µL, 889 mg, 6.26 mmol, 10.0 eq.) was added. The reaction mixture was stirred overnight under reflux before being diluted with $Et_2O$. The organic layers were dried, filtered and concentrated in vacuo to give the elimination product **19a** (189 mg, 0.61 mmol, 97%) as yellow oil. Analytical data were in agreement the literature values.[2]

**ORD**: $[\alpha]_D$ = –71.9 (c = 0.74 in $CHCl_3$). **$^1$H-NMR** (300 MHz, $CDCl_3$): δ = 6.94–6.90 (m, 1 H, *H*C=C), 5.51 (d, *J* = 2.7 Hz, 1 H, C=C*H*$_2$), 4.83 –4.78 (m, 2 H, C=C*H*$_2$, C*H*O), 4.43–4.35 (m, 2 H, C*H*O), 3.77 (s, 3 H, $CO_2CH_3$), 3.76 (s, 3 H, $CO_2CH_3$), 2.85–2.70 (m, 1 H, HC=CC*H*$_2$), 2.56–2.39 (m, 1 H, HC=CC*H*$_2$), 1.40 (s, 3 H, CC*H*$_3$), 1.39 (s, 3 H, CC*H*$_3$). **MS** (ESI+): *m/z* = 335.0 $[M+Na]^+$.

**Methyl (3*R*,4*R*,5*R*)-3,4-dihydroxy-5-((3-methoxy-3-oxoprop-1-en-2-yl)oxy)cyclohex-1-ene-1-carboxylate (20)**

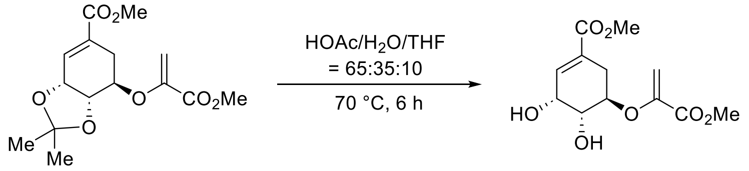


**19a** **20**

The reaction was conducted according to a published procedure.[2] The Hofmann elimination product **19a** (875 mg, 2.80 mmol, 1.0 eq.) was dissolved in mixture of 65:35:10 acetic acid/water/tetrahydrofuran (6.0 mL). The reaction mixture was stirred for 6 h at 70 °C before being diluted with DCM. The organic layer was washed with saturated $Na_2CO_3$ solution, brine, dried, filtered and concentrated in vacuo to give the desired product **20** (667 mg, 2.45 mmol, 87%) as a slightly yellowish oil. Analytical data were in agreement with literature values.[2]

**ORD**: $[\alpha]_D$ = –129.7 (c = 0.91 in $CHCl_3$). **$^1$H-NMR** (600 MHz, $CDCl_3$): δ = 6.91–6.87 (m, 1 H, *H*C=C), 5.55 (d, *J* = 2.5 Hz, 1 H, C=C*H*$_2$), 4.93 (d, *J* = 2.6 Hz, 1 H, C=C*H*$_2$), 4.55 (t, *J* = 4.3 Hz, 1 H, C*H*O), 4.37–4.30 (m, 1 H, C*H*O), 3.96 (dd, *J* = 8.6, 4.2 Hz, 1 H, C*H*O), 3.80 (s, 3 H, $CO_2CH_3$), 3.75 (s, 3 H, $CO_2CH_3$), 2.99 (dd, *J* = 18.1, 5.3 Hz, 1 H, HC=CC*H*$_2$), 2.33 (dd, *J* = 18.1, 7.7 Hz, 1 H, HC=CC*H*$_2$). **MS** (ESI+): *m/z* = 295.2 $[M+Na]^+$.

**(8*R*)-Scytolide – Methyl (4a*R*,8*R*,8a*R*)-8-hydroxy-3-methylene-2-oxo-2,3,4a,5,8,8a-hexahydrobenzo[*b*][1,4]dioxine-6-carboxylate (21a)**

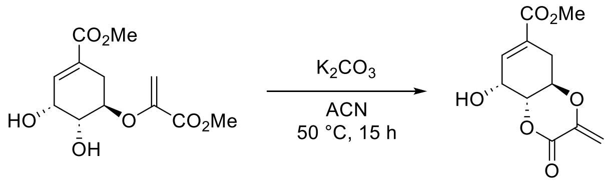


The reaction was conducted according to a published procedure.[2] The unprotected species **20** (1.00 g, 3.67 mmol, 1.0 eq.) and $K_2CO_3$ (25.4 mg, 0.18 mmol, 5 mol%) was dissolved in acetonitrile (55.0 mL, 15.0 mL/mmol) and the obtained reaction mixture was stirred for 15 h at 50 °C. After dilution with saturated $NH_4Cl$ solution, the reaction mixture was extracted with DCM (3x). The combined organic layers were washed with brine, dried, filtered and concentrated in vacuo to give the desired product **21a** (819 mg, 3.41 mmol, 93%) as a white solid. Analytical data were in agreement with literature values.[2]

**ORD**: $[\alpha]_D$ = –112.0 (c = 0.20 in $CHCl_3$). **TLC**: $R_f$ (DCM/$Et_2O$ = 4:1) = 0.38. **$^1$H-NMR** (300 MHz, $CDCl_3$): δ = 6.94 (ddd, *J* = 5.6, 2.9, 1.1 Hz, 1 H, *H*C=C), 5.71 (d, *J* = 1.5 Hz, 1 H, C=C*H*$_2$), 5.13 (d, *J* = 1.5 Hz, 1 H, C=C*H*$_2$), 4.68–4.59 (m, 1 H, C*H*OH), 4.48–4.28 (m, 2 H, C*H*OC), 3.80 (s, 3 H, $CO_2CH_3$), 3.18 (ddd, *J* = 17.3, 5.9, 0.8 Hz, 1 H, HC=CC*H*$_2$), 2.63 (bs, 1 H, O*H*), 2.37 (ddd, *J* = 17.3, 8.5, 2.8 Hz, 1 H, HC=CC*H*$_2$). **MS** (ESI+): *m/z* = 240.8 $[M+H]^+$.

**Scytolide (11)**

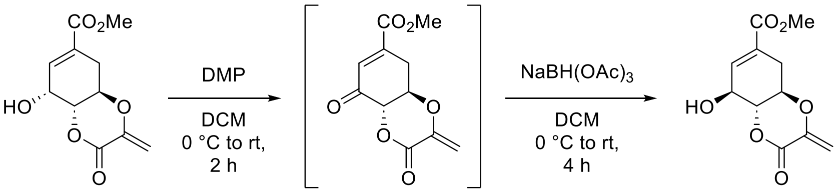


(8*R*)-Scytolide (**21a**, 100 mg, 0.42 mmol, 1.0 eq.) was dissolved in dry DCM (12.0 mL, 15.0 mL/mmol) and cooled down to 0 °C. Dess-Martin periodinane (212mg, 0.50 mmol, 1.2 eq.) was added to the reaction mixture. The reaction suspension was allowed to warm up to room temperature and was stirred for 2 h at room temperature before being cooled down to 0 °C. After addition of sodium triacetoxyborohydride (352 mg, 1.66 mmol, 4.0 eq.), the reaction mixture was stirred for 4 h at room temperature before being treated with saturated $NaHCO_3$ solution (see Table in Scheme 1, entry c). The organic layer was separated, washed with brine, dried, filtered and concentrated in vacuo. The crude material was purified by MPLC (gradient, 5–70% ACN in 30 min) to give a mixture (5:1) of scytolide (**11,** 75.0 mg, 0.31 mmol, 75%) and (8*R*)-scytolide (**21a**, 15.0 mg, 0.06 mmol, 15%) as white solid. The same reaction was performed with sodium borohydride (4.0 eq.) leading to 70% conversion and a product ratio of **11**:**21a** = 3:1 (see Table in Scheme 1, entry a). The usage of sodium triacetoxyborohydride (4.0 eq.) at room temperature reached a yield of 27% with a product ratio of **11**:**21a** = 2:1 (see Table in Scheme 1, entry b). Due to the instability of the compounds (prone to spontaneous

polymerization), the complete fractions after MPLC purification were used for further chemo-enzymatic reactions. For the analysis of the oxidized species **22a** and of scytolide (**11**), in each case one aliquot was purified by MPLC (gradient, 5–70% ACN in 25 min), or preparative HPLC (gradient, 5–95% ACN in 60 min). Analytical data of **11** were in agreement with literature values.[3,4]

Oxoscytolide (**22a**)
**ORD**: $[\alpha]_D$ =–46.8 (c = 0.10 in $CHCl_3$). **TLC**: $R_f$ (DCM/$Et_2O$ = 4:1) = 0.38. **$^1$H-NMR** (300 MHz, DMSO-$d_6$): δ = 6.68 (d, *J* = 3.0 Hz, 1 H, *H*C=C), 5.51 (d, *J* = 1.5 Hz, 1 H, C=C$H_2$), 5.50 (d, *J* = 11.1 Hz, 1 H, C(O)C*H*OC), 5.15 (d, *J* = 1.6 Hz, 1 H, C=C$H_2$), 4.68 (ddd, *J* = 11.2, 10.0, 5.8 Hz, 1 H, C*H*OC), 3.81 (s, 3 H, $CO_2CH_3$), 3.22 (dd, *J* = 17.9, 5.8 Hz, 1 H, HC=CC$H_2$), 2.79 (ddd, *J* = 17.9, 10.0, 3.2 Hz, 1 H, HC=CC$H_2$). **$^1$H-NMR** (300 MHz, $CDCl_3$): δ = 6.92 (d, *J* = 3.2 Hz, 1 H), 5.76 (d, *J* = 1.7 Hz, 1 H), 5.18 (d, *J* = 1.7 Hz, 1 H), 4.94 (d, *J* = 11.1 Hz, 1 H), 4.33 (ddd, *J* = 11.2, 10.1, 5.8 Hz, 1 H), 3.89 (s, 3 H), 3.44 (dd, *J* = 18.7, 5.8 Hz, 1 H), 2.76 (ddd, *J* = 18.7, 10.1, 3.2 Hz, 1 H). **$^{13}$C-NMR** (75 MHz, DMSO-$d_6$): δ = 190.2, 165.2, 158.2, 146.0, 145.0, 130.9, 103.7, 80.0, 70.8, 53.0, 29.6. **$^{13}$C-NMR** (75 MHz, $CDCl_3$): δ = 189.1, 165.4, 157.5, 145.6, 144.4, 129.2, 206.1, 80.6, 71.6, 53.5, 30.2. **HRMS** (ESI+) *m/z* calcd for $C_{11}H_{10}NaO_6$ $[M+Na]^+$: 261.0370; found: 261.0374.

Scytolide (**11**)
**ORD**: $[\alpha]_D$ = –30.6 (c = 0.11 in $CHCl_3$). **$^1$H-NMR** (300 MHz, $CDCl_3$): δ = 6.78 (t, *J* = 2.2 Hz, 1 H, *H*C=C), 5.71 (d, *J* = 1.7 Hz, 1 H, C=C$H_2$), 5.13 (d, *J* = 1.5 Hz, 1 H, C=C$H_2$), 4.64–4.57 (m, 1 H, C*H*OH), 4.41 (dd, *J* = 10.4, 8.0 Hz, 1 H, C*H*OC), 4.11 (td, *J* = 10.1, 6.5 Hz, 1 H, C*H*OC), 3.80 (s, 3 H, $CO_2CH_3$), 3.08 (dd, *J* = 17.7, 7.0 Hz, 1 H, HC=CC$H_2$), 2.52–2.43 (m, 1 H, HC=CC$H_2$). **MS** (ESI+): *m/z* = 240.8 $[M+H]^+$.

## 2.2 Synthesis of (8*R*)-*epi*-Scytolide (21b) and *epi*-Scytolide (23)

### 3,4-*O*-Isopropylidenequinic acid-1,5-lactone (15)

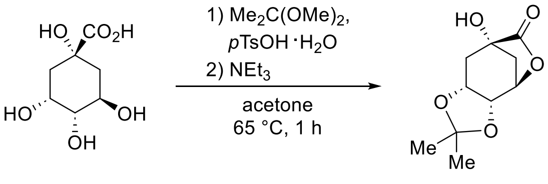


**14** **15**

The reaction was conducted according to a published procedure.[5] (–)-Quinic acid (**14**, 40.5 g, 211 mmol, 1.0 eq.), 2.2-dimethoxypropane (92.7 mL, 78.8 g, 757 mmol, 3.6 eq.), *p*-toluenesulfonic acid monohydrate (4.01 g, 21.1 mmol, 0.1 eq.) were dissolved in acetone (1.40 L, 6.0 mL/mmol). The reaction mixture was stirred for 1 h at 65 °C before being treated with triethylamine (8.09 mL, 6.31 g, 62.4 mmol, 0.3 eq.). The mixture was filtered and concentrated under reduced pressure. The obtained residue was purified by column chromatography (*n*-pentane/EtOAc = 3:1) to give the desired product **15** (44.3 g, 207 mmol, 98%) as a white solid. Analytical data were in agreement with literature values.[5]

**ORD**: $[\alpha]_D$ = –29.1 (c = 0.39 in MeOH). **TLC**: $R_f$ (*n*-pentane/EtOAc = 3:1) = 0.73. **$^1$H-NMR** (300 MHz, $CDCl_3$): δ = 4.72 (dd, *J* = 6.1, 2.5 Hz, 1 H, C*H*OC), 4.49 (td, *J* = 7.0, 2.6 Hz, 1 H, C*H*OC), 4.30 (ddd, *J* = 6.5, 2.4, 1.3 Hz, 1 H, C*H*OC), 2.64 (d, *J* = 11.8 Hz, 1 H, $CH_2$), 2.43–2.24 (m, 2 H, $CH_2$), 2.17 (dd, *J* = 14.7, 3.0 Hz, 1 H, $CH_2$), 1.52 (s, 3 H, $CCH_3$), 1.32 (s, 3 H, $CCH_3$). **$^1$H-NMR** (300 MHz, DMSO-$d_6$): δ = 6.06 (s, 1 H, O*H*), 4.67 (dd, *J* = 5.9, 2.6 Hz, 1 H, C*H*OC), 4.52–4.40 (m, 1 H, C*H*OC), 4.24 (ddd, *J* = 6.5, 2.6, 1.2 Hz, 1 H, C*H*OC), 2.37–2.15 (m, 3 H, $CH_2$), 1.86 (dd, *J* = 14.5, 3.1 Hz, 1 H, $CH_2$), 1.43 (s, 3 H, $CCH_3$), 1.25 (s, 3 H, $CCH_3$). **MS** (ESI+): *m/z* = 237.2 $[M+Na]^+$.

### Methyl 3,4-*O*-Isopropylidenequinate (16)

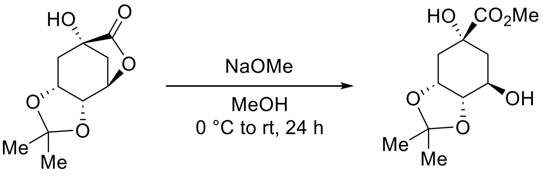


**15** **16**

The reaction was conducted according to a published procedure.[6] The lactone **15** (5.00 g, 23.3 mmol, 1.0 eq.) was dissolved in dry methanol (105 mL, 4.5 mL/mmol) and cooled to 0 °C. Sodium methanolate (1.64 g, 30.3 mmol, 1.3 eq.) was added and the reaction mixture was allowed to warm up to room temperature. The reaction mixture was stirred for 4 h at room temperature before being treated with acetic acid (1.50 mL) and DCM. The separated organic layer was washed with saturated $NaHCO_3$ solution, brine, dried, filtered and concentrated in vacuo. The obtained residue was purified by column chromatography (*n*-pentane/EtOAc = 1:1) to give the desired product **16** as a colorless oil (3.99 g, 18.6 mmol, 80%). Analytical data were in agreement with literature values.[6]

**ORD**: $[\alpha]_D$ = –42.2 (c = 1.54 in $CHCl_3$). **TLC**: $R_f$ (EtOAc) = 0.47. **$^1$H-NMR** (300 MHz, $CDCl_3$): δ = 4.52–4.42 (m, 1 H, C*H*O), 4.20–4.03 (m, 1 H, C*H*O), 3.98 (t, *J* = 6.3 Hz, 1 H, C*H*O), 3.81 (s, 3 H,

$CO_2CH_3$), 2.25 (d, *J* = 3.4 Hz, 2 H, $CH_2$), 2.12–2.05 (m, 1 H, $CH_2$), 1.87 (dd, *J* = 13.6, 10.6 Hz, 1 H, $CH_2$), 1.54 (s, 3 H, $CCH_3$), 1.36 (s, 3 H, $CCH_3$). **$^1$H-NMR** (300 MHz, DMSO-d$_6$): δ = 5.36 (s, 1 H, CO*H*), 4.92 (d, *J* = 4.7 Hz, 1 H, CHO*H*), 4.27 (q, *J* = 5.8 Hz, 1 H, C*H*O), 4.03 (q, *J* = 7.1 Hz, 1 H, C*H*O), 3.93–3.75 (m, 1 H, C*H*O), 3.62 (s, 3 H, $CO_2CH_3$), 2.11 (dd, *J* = 14.4, 5.6 Hz, 1 H, $CH_2$), 1.81–1.59 (m, 3 H, $CH_2$), 1.38 (s, 3 H, $CCH_3$), 1.24 (s, 3 H, $CCH_3$). **MS** (ESI+): *m/z* = 268.9 $[M+Na]^+$.

**Methyl 4,5-*O*-Isopropylidene-3-oxo-4-epi-shikimate**

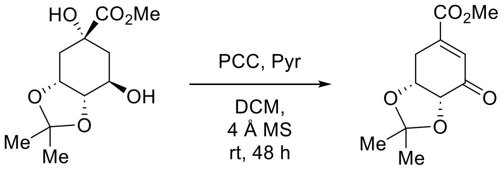


**16**

The reaction was conducted according to a published procedure.[6,7] Methyl 3,4-*O*-isopropylidenequinate (**16**, 3.55 g, 14.4 mmol, 1.0 eq.), pyridine (3.62 mL, 3.55 g, 44.9 mmol, ζ = 1), pyridinium chlorochromate (12.4 g, 57.6 mmol, 4.0 eq.), silica (10.0 g) and powdered 4 Å molecular sieves (7.10 g, ζ = 2) was suspended in dry DCM (86.4 mL, 6.0 mL/mmol). The reaction solution was stirred for 48 h at room temperature before being filtered through a celite pad. The filtrate was concentrated in vacuo and the obtained residue was purified by column chromatography (*n*-pentane/EtOAc = 1:1) to give the desired product as a slightly yellowish solid (1.42 g, 6.28 mmol, 44%). The uneliminated derivative was also obtained in 20% yield (0.70 g, 2.88 mmol). Direct transformation into the eliminated species using phosphoryl chloride and pyridine[2] increased the total yield by 16% (0.56 g, 2.30 mmol) to an overall yield of 60%. Analytical data were in agreement with the literature values.[6,7]
**ORD**: $[\alpha]_D$ = –50.2 (c = 0.59 in $CHCl_3$). **TLC**: $R_f$ (EtOAc) = 0.77. **$^1$H-NMR** (300 MHz, $CDCl_3$): δ = 6.85 (dd, *J* = 2.8, 1.0 Hz, 1 H, C=C*H*), 4.70 (td, *J* = 5.0, 1.5 Hz, 1 H, C*H*O), 4.31 (d, *J* = 5.0 Hz, 1 H, C*H*O), 3.86 (s, 3 H, $CO_2CH_3$), 3.22 (d, *J* = 20.4 Hz, 1 H, $CH_2$), 2.88 (ddd, *J* = 20.2, 5.0, 2.8 Hz, 1 H, $CH_2$), 1.41 (s, 3 H, $CCH_3$), 1.33 (s, 3 H, $CCH_3$). **MS** (ESI+): *m/z* = 248.9 $[M+Na]^+$.

**Methyl 4,5-*O*-Isopropylidene-4-epi-shikimate (17b)**

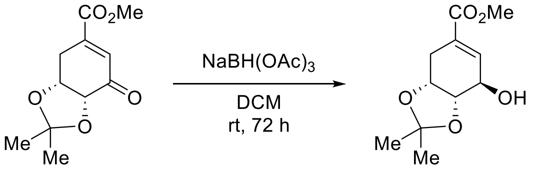


**17b**

The reaction was prepared according to a published procedure.[6] Methyl 4,5-*O*-Isopropylidene-3-oxo-4-epi-shikimate (1.40 g, 6.19 mmol, 1.0 eq.) was dissolved in dry DCM (80.5 mL, 13.0 mL/mmol). After addition of sodium triacetoxyborohydride (2.62 g, 12.4 mmol, 2.0 eq.), the reaction solution was stirred for 72 h at room temperature before being treated with saturated $NaHCO_3$ solution. The organic layer was separated, washed with brine, dried,

filtered and concentrated in vacuo to give the desired product **17b** as a yellow oil (1.10 g, 4.82 mmol, 78%).

**ORD**: $[\alpha]_D$ = +49.8 (c = 3.27 in $CHCl_3$). **TLC**: $R_f$ (EtOAc) = 0.75. **$^1$H-NMR** (300 MHz, $CDCl_3$): δ = 6.96–6.93 (m, 1 H, C=C*H*), 4.65 (ddd, *J* = 7.4, 3.9, 2.2 Hz, 1 H, C*H*OH), 4.56 (ddd, *J* = 7.4, 4.8, 1.5 Hz, 1 H, C*H*O), 4.13–4.02 (m, 1 H, C*H*O), 3.77 (s, 3 H, $CO_2CH_3$), 3.04 (dd, *J* = 16.4, 2.4 Hz, 1 H, $CH_2$), 2.67 (d, *J* = 10.3 Hz, 1 H, O*H*), 1.95 (ddd, *J* = 16.4, 6.2, 2.9 Hz, 1 H, $CH_2$), 1.33 (s, 3 H, $CCH_3$), 1.32 (s, 3 H, $CCH_3$). **$^{13}$C-NMR** (75 MHz, $CDCl_3$): δ = 166.3, 142.6, 128.5, 109.2, 76.2, 72.6, 68.1, 52.1, 26.6, 26.0, 24.5. **HRMS** (ESI+) *m/z* calcd for $C_{11}H_{16}NaO_5$ $[M+Na]^+$: 251.0890; found: 251.0886.

**Dimethyl 2-(((3a*R*,4*R*,7a*R*)-6-(methoxycarbonyl)-2,2-dimethyl-3a,4,7,7a-tetrahydrobenzo [*d*][1,3]dioxol-4-yl)oxy)malonate (18b)**

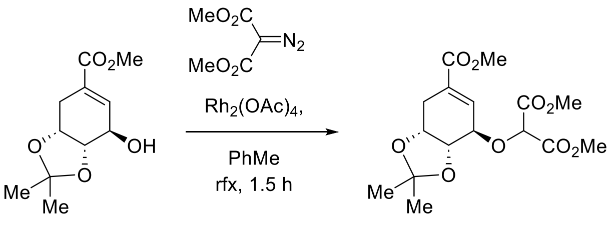


**17b** **18b**

The reaction was performed as described for compound **18a**. Purification was performed by MPLC (gradient, 5–95% ACN in 25 min). The product (2.41 g, 6.73 mmol, 53%) was obtained as colorless oil.

**ORD**: $[\alpha]_D$ = +24.8 (c = 1.33 in $CHCl_3$). **$^1$H-NMR** (300 MHz, $CDCl_3$): δ = 7.03 (s, 1 H, C=C*H*), 4.87 (s, 1 H, C*H*($CO_2Me)_2$), 4.72 (ddd, *J* = 7.5, 3.8, 1.7 Hz, 1 H, C*H*O), 4.59 (ddd, *J* = 7.4, 4.2, 1.9 Hz, 1 H, C*H*O), 4.17 (dt, *J* = 3.9, 2.4 Hz, 1 H, C*H*O), 3.812 (s, 3 H, $CO_2CH_3$), 3.808 (s, 3 H, $CO_2CH_3$), 3.75 (s, 3 H, $CO_2CH_3$), 3.01 (dd, *J* = 16.5, 1.9 Hz, 1 H, $CH_2$), 1.93–1.82 (m, 1 H, $CH_2$), 1.29 (s, 3 H, $CCH_3$), 1.28 (s, 3 H, $CCH_3$). **$^{13}$C-NMR** (75 MHz, $CDCl_3$): δ = 167.1, 167.0, 165.9, 139.1, 129.7, 109.4, 77.4, 76.5, 75.5, 72.8, 53.2, 53.1, 52.1, 27.4, 25.9, 24.3. **HRMS** (ESI+) *m/z* calcd for $C_{16}H_{22}NaO_9$ $[M+Na]^+$: 381.1156; found: 381.1154.

**Dimethyl 2-((dimethylamino)methyl)-2-(((3a*R*,4*R*,7a*R*)-6-(methoxycarbonyl)-2,2-dimethyl-3a,4,7,7a-tetrahydrobenzo[*d*][1,3]dioxol-4-yl)oxy)malonate**

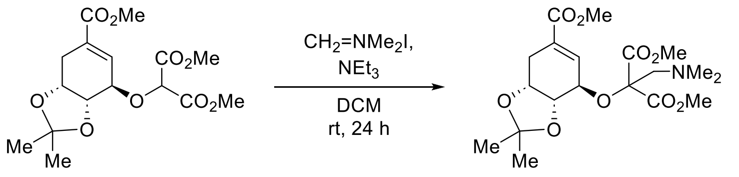


**18b**

The reaction was performed as described for the isomeric compound (dimethyl 2-((dimethylamino)methyl)-2-(((3a*S*,4*R*,7a*R*)-6-(methoxycarbonyl)-2,2-dimethyl-3a,4,5,7a-tetrahydrobenzo[*d*][1,3]dioxol-4-yl)oxy)malonate). The desired product was obtained as a yellowish oil (678 mg, 1.63 mmol, 97%) after extraction.

**ORD**: $[\alpha]_D$ = +33.5 (c = 1.76 in $CHCl_3$). **$^1$H-NMR** (300 MHz, $CDCl_3$): δ = 6.93–6.86 (m, 1 H, C=C*H*), 4.81 (ddd, *J* = 7.4, 3.8, 1.6 Hz, 1 H, C*H*O), 4.55 (ddd, *J* = 7.4, 4.3, 1.9 Hz, 1 H, C*H*O), 4.45–4.36 (m, 1 H, C*H*O), 3.78 (s, 3 H, $CO_2CH_3$), 3.74 (s, 3 H, $CO_2CH_3$), 3.72 (s, 3 H, $CO_2CH_3$), 2.98–2.90 (m, 1 H, $CH_2$), 2.34 (s, 6 H, $N(CH_3)_2$), 1.94–1.79 (m, 1 H, $CH_2$), 1.28 (s, 3 H, $CCH_3$), 1.27 (s, 3 H, $CCH_3$). **$^{13}$C-NMR** (75 MHz, $CDCl_3$): δ = 169.1, 168.4, 166.2, 142.1, 127.9, 108.8, 87.3, 77.0, 74.5, 72.9, 63.5, 52.7, 52.6, 51.9, 47.6, 27.4, 26.0, 24.4. **HRMS** (ESI+) *m/z* calcd for $C_{19}H_{30}NO_9$ $[M+H]^+$: 416.1915; found: 416.1907.

**Methyl (3a*R*,7*R*,7a*R*)-7-((3-methoxy-3-oxoprop-1-en-2-yl)oxy)-2,2-dimethyl-3a,4,7,7a-tetrahydrobenzo[*d*][1,3]dioxole-5-carboxylate (19b)**

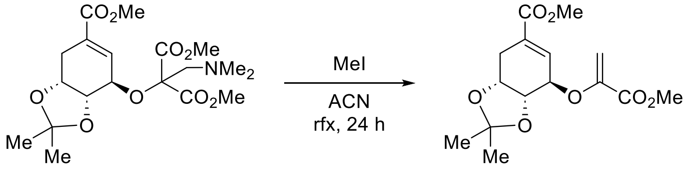


**19b**

The reaction was performed as described for compound **19a**. The desired product **19b** was obtained as a yellow oil (356 mg, 1.14 mmol, 73%) after extraction.

**ORD**: $[\alpha]_D$ = +60.4 (c = 2.93 in $CHCl_3$). **$^1$H-NMR** (300 MHz, $CDCl_3$): δ = 6.98–6.94 (m, 1 H, C=C*H*), 5.54 (d, *J* = 2.8 Hz, 1 H, C=$CH_2$), 4.76 (ddd, *J* = 7.4, 3.6, 1.7 Hz, 1 H, C*H*O), 4.71 (d, *J* = 2.8 Hz, 1 H, C=$CH_2$), 4.63 (ddd, *J* = 7.4, 4.2, 1.8 Hz, 1 H, C*H*O), 4.34 (dt, *J* = 4.0, 2.2 Hz, 1 H, C*H*O), 3.81 (s, 3 H, $CO_2CH_3$), 3.75 (s, 3 H, $CO_2CH_3$), 3.07 (d, *J* = 16.4 Hz, 1 H, $CH_2$), 1.98–1.91 (m, 1 H, $CH_2$), 1.32 (s, 3 H, $CCH_3$), 1.31 (s, 3 H, $CCH_3$). **$^{13}$C-NMR** (75 MHz, $CDCl_3$): δ = 165.8, 163.4, 150.2, 138.3, 129.7, 109.6, 98.8, 75.3, 75.1, 72.6, 52.6, 52.1, 27.6, 25.9, 24.4. **HRMS** (ESI+) *m/z* calcd for **HRMS** (ESI+) *m/z* calcd for $C_{15}H_{20}NaO_7$ $[M+Na]^+$: 335.1101; found: 335.1096.

**Cyathiformine D – Methyl (3*R*,4*R*,5*R*)-4,5-dihydroxy-3-((3-methoxy-3-oxoprop-1-en-2-yl)oxy)cyclohex-1-ene-1-carboxylate (9)**

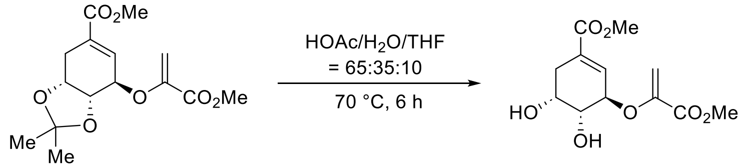


**19b** **9**

The reaction was performed as described for compound **20a**. The desired product **9** was obtained as a white solid (261 mg, 0.96 mmol, 86%) after extraction. Analytical data were in agreement with literature values.[8]

**ORD**: $[\alpha]_D$ = +69.5 (c = 0.14 in acetonitrile), +97.2° (c = 0.16 in $CHCl_3$). **$^1$H-NMR** (300 MHz, acetonitrile-$d_3$): δ = 6.61–6.57 (m, 1 H, C=C*H*), 5.48 (d, *J* = 3.0 Hz, 1 H, C=$CH_2$), 4.92 (d, *J* = 3.0 Hz, 1 H, C=$CH_2$), 4.82–4.77 (m, 1 H, C*H*O), 4.14 (bs, 1 H, C*H*O), 3.87–3.79 (m, 1 H, C*H*O), 3.76 (s, 3 H, $CO_2CH_3$), 3.71 (s, 3 H, $CO_2CH_3$), 3.18 (bs, 1 H, O*H*), 3.09 (bs, 1 H, O*H*), 2.55 (ddt, *J* = 17.4, 6.0, 1.4 Hz, 1 H, $CH_2$), 2.29 (ddt, *J* = 17.4, 9.6, 3.2 Hz, 1 H, $CH_2$). **$^{13}$C-NMR** (75 MHz,

acetonitrile-d3): δ = 166.9, 164.5, 150.5, 134.4, 131.9, 98.2, 76.2, 68.8, 68.4, 53.0, 52.5, 30.0. **MS** (ESI+): *m/z* = 295.6 $[M+Na]^+$.

**(8*R*)-*epi*-Scytolide – Methyl (4a*R*,8*R*,8a*R*)-8-hydroxy-3-methylene-2-oxo-2,3,4a,7,8,8a-hexahydrobenzo[*b*][1,4]dioxine-6-carboxylate (21b)**

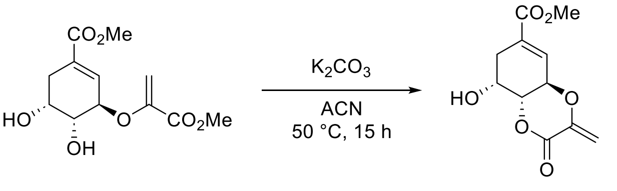


The reaction was performed as described for compound **21a**. The desired product **21b** was obtained as a yellow oil (206 mg, 0.86 mmol, 93%) after extraction.

**ORD**: $[\alpha]_D$ = +103.7 (c = 0.26 in acetonitrile). **TLC**: $R_f$ (DCM/$Et_2O$ = 4:1) = 0.26. **$^1$H-NMR** (300 MHz, acetonitrile-d3): δ = 6.61–6.56 (m, 1 H, C=C*H*), 5.54 (d, *J* = 1.4 Hz, 1 H, C=C$H_2$), 5.06 (d, *J* = 1.4 Hz, 1 H, C=C$H_2$), 4.96–4.91 (m, 2 H, C*H*O), 4.05–3.96 (m, 1 H, C*H*O), 3.72 (s, 3 H, $CO_2CH_3$), 2.74–2.64 (m, 1 H, C$H_2$), 2.36–2.23 (m, 1 H, C$H_2$). **$^1$H-NMR** (300 MHz, $CDCl_3$): δ = 6.70 (dt, *J* = 3.0, 1.7 Hz, 1 H, C=C*H*), 5.72 (d, *J* = 1.5 Hz, 1 H, C=C$H_2$), 5.15 (d, *J* = 1.5 Hz, 1 H, C=C$H_2$), 5.01–4.97 (m, 1 H, C*H*O), 4.89–4.82 (m, 1 H, C*H*O), 4.02 (ddd, *J* = 9.6, 5.0, 1.7 Hz, 1 H, C*H*O), 3.72 (s, 3 H, $CO_2CH_3$), 2.88 (ddd, *J* = 17.9, 4.8, 3.1 Hz, 1 H, C$H_2$), 2.44 (ddt, *J* = 18.0, 9.7, 3.2 Hz, 1 H, C$H_2$). **$^{13}$C-NMR** (75 MHz, acetonitrile-d3): δ = 166.5, 159.9, 146.4, 133.9, 133.4, 105.0, 78.1, 70.1, 67.0, 52.8, 29.7. **$^{13}$C-NMR** (75 MHz, $CDCl_3$): δ = 165.4, 158.8, 144.6, 132.9, 132.6, 106.7, 77.0, 69.3, 67.4, 52.5, 29.5. **HRMS** (ESI+) *m/z* calcd for $C_{11}H_{12}NaO_6$ $[M+Na]^+$: 263.0526; found: 263.0524.

***epi*-Scytolide (23)**

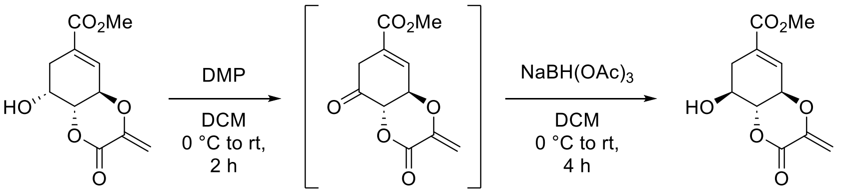


The reaction was performed as described for compound **11** (method c). Purification was performed by MPLC (gradient, 5–70% ACN in 30 min) to give a crude mixture (4:1) of *epi*-scytolide (**23,** 50.3 mg, 0.21 mmol, 17%) and (8*R*)-*epi*-scytolide (**21b**, 12.7 mg, 52.9 µmol, 4%) as white solid.

*epi*-Scytolide (**23**)

**$^1$H-NMR** (300 MHz, MeOD): δ = 6.65 (dt, *J* = 2.7, 1.5 Hz, 1 H, C=C*H*), 5.59 (d, *J* = 1.4 Hz, 1 H, C=C$H_2$), 5.10 (d, *J* = 1.4 Hz, 1 H, C=C$H_2$), 5.05–4.94 (m, 2 H, C*H*O), 4.04 (ddd, *J* = 9.8, 6.1, 1.7 Hz, 1 H, C*H*O), 3.76 (s, 3 H, $CO_2CH_3$), 2.74 (dd, *J* = 17.9, 6.0 Hz, 1 H, C$H_2$), 2.38 (ddt, *J* = 17.9, 9.7, 3.2 Hz, 1 H, C$H_2$). **MS** (ESI+): *m/z* = 503.0 $[2M+Na]^+$, 240.9 $[M+H]^+$.

## 2.3 Chemo-enzymatic Total Synthesis of the Spirosorbicillinols

### Spirosorbicillinol A (5) and Spirosorbicillinol B (6)

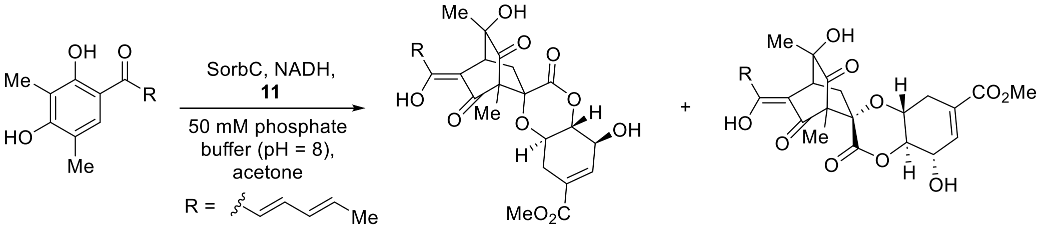


The chemo-enzymatic reaction was performed with sorbicillin as substrate (25.1 mg, 108 µmol, 1.0 eq.), 230 mg NADH-$Na_2$ (324 µmol, 3.0 eq.), 10.0 mg SorbC (11.1 mg/mL stock solution, 0.91 mL, 0.21 µmol, 0.2 mol%) being dissolved in acetone (5.4 mL) and phosphate buffer (48.6 mL). The reaction was incubated at 4 °C and 150 rpm (GFL 3005) for 4 h before being extracted with DCM (3x200 mL). During workup, scytolide (**11**, excess, around 5.0 eq.) was added and the solvent was evaporated under reduced pressure, to increase the concentration of dienophile slowly over time. The crude material was purified by semi-preparative HPLC (gradient, 5–95% ACN in 60 min) to give spirosorbicllinol A (**5**, 2.30 mg, 4.71 µmol, 4%) and spirosorbicillinol B (**6**, 13.4 mg, 27.4 µmol, 25%) as slightly yellow oils. The substrate was re-isolated in 51% yield (12.8 mg, 55.1 µmol), based on which the desired products were obtained in 9% (**5**) and 52% (**6**) yield (brsm). Bisorbicillinol (**1**, 2.10 mg, 4.23 µmol, 8%, brsm: 16%) was obtained as a side product. Analytical data were in agreement the literature values.[9]

Spirosorbicillinol A (**5**)

**ORD**: $[\alpha]_D$ = +184.2 (c = 0.15 in MeOH). **$^1$H-NMR** (300 MHz, MeOD): δ = 7.33 (dd, *J* = 14.9, 10.7 Hz, 1 H, sorbyl-C*H*), 6.65 (dd, *J* = 2.4, 2.1 Hz, 1 H, C=C*H*), 6.48–6.35 (m, 2 H, sorbyl-C*H*), 6.30–6.16 (m, 1 H, sorbyl-C*H*), 4.50–4.40 (m, 1 H, C*H*OH), 4.27 (dd, *J* = 10.0, 8.1 Hz, 1 H, C*H*O), 4.08 (td, *J* = 9.9, 6.2 Hz, 1 H, C*H*O), 3.74 (s, 3 H, $CO_2CH_3$), 3.32–3.30 (m, 1 H, C*H*, overlap with MeOD signal), 3.05 (dd, *J* = 14.1, 2.4 Hz, 1 H, $CH_2$), 2.80 (dd, *J* = 17.2, 6.3 Hz, 1 H, $CH_2$), 2.31–2.22 (m, 1 H, $CH_2$), 2.18 (dd, *J* = 14.1, 3.6 Hz, 1 H, $CH_2$), 1.89 (d, *J* = 6.7 Hz, 3 H, sorbyl-$CH_3$), 1.23 (s, 3 H, $CCH_3$), 1.20 (s, 3 H, $CCH_3$). **HRMS** (ESI+) *m/z* calcd for $C_{25}H_{28}NaO_{10}$ $[M+Na]^+$: 511.1575; found: 511.1580. **MS** (ESI+): *m/z* = 510.9 $[M+Na]^+$, 489.2 $[M+H]^+$.

Spirosorbicillinol B (**6**)

**ORD**: $[\alpha]_D$ = +308.6 (c = 0.59 in MeOH). **$^1$H-NMR** (300 MHz, MeOD): δ = 7.30 (dd, *J* = 14.9, 10.9 Hz, 1 H, sorbyl-C*H*), 6.65 (dd, *J* = 2.9, 2.2 Hz, 1 H, C=C*H*), 6.46–6.32 (m, 2 H, sorbyl-C*H*), 6.28–6.12 (m, 1 H, sorbyl-C*H*), 4.51–4.41 (m, 1 H, C*H*OH), 4.37 (dd, *J* = 10.0, 7.8 Hz, 1 H, C*H*O), 4.01 (td, *J* = 9.8, 6.3 Hz, 1 H, C*H*O), 3.75 (s, 3 H, $CO_2CH_3$), 3.32–3.30 (m, 1H, C*H*, overlap with MeOD signal), 2.98 (dd, *J* = 14.0, 3.0 Hz, 1 H, $CH_2$), 2.89 (dd, *J* = 17.6, 5.9 Hz, 1 H, $CH_2$), 2.38–2.23 (m, 2 H, $CH_2$), 1.88 (dd, *J* = 6.8, 1.4 Hz, 3 H, sorbyl-$CH_3$), 1.25 (s, 3 H, $CCH_3$), 1.19 (s, 3 H, $CCH_3$). **HRMS** (ESI+) *m/z* calcd for $C_{25}H_{28}NaO_{10}$ $[M+Na]^+$: 511.1575; found: 511.1583. **MS** (ESI+): *m/z* = 489.1 $[M+H]^+$.

## Synthesized Spirosorbicillinol C (7)

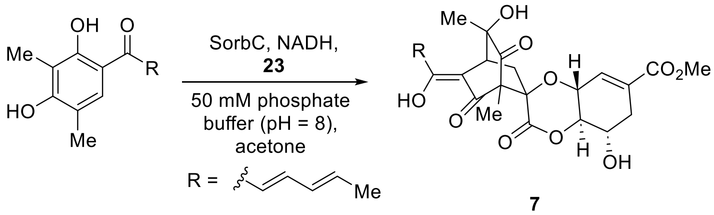


The chemo-enzymatic reaction was performed with sorbicillin as substrate (25.1 mg, 108 µmol, 1.0 eq.), 230 mg NADH-$Na_2$ (324 µmol, 3.0 eq.), 10.0 mg SorbC (11.1 mg/mL stock solution, 0.91 mL, 0.21 µmol, 0.2 mol%) being dissolved in acetone (5.4 mL) and phosphate buffer (48.6 mL). The reaction was incubated at 4 °C and 150 rpm (GFL 3005) for 4 h before being extracted with DCM (3x200 mL). During workup, *epi*-scytolide (**23**, excess, around 5.0 eq.)) was added and the solvent was evaporated under reduced pressure, to increase the concentration of dienophile slowly over time. The crude material was purified by semi-preparative HPLC (gradient, 5–95% ACN in 60 min) to give spirosorbicllinol C (**7**, 14.2 mg, 26.9 µmol, 25%) as slightly yellow oil. The substrate was re-isolated in 45% yield (11.3 mg, 48.6 µmol), based on which the desired product was obtained in 45% yield (brsm). Bisorbicillinol (**1**, 7.30 mg, 14.7 µmol, 27%, brsm: 49%) and (12*R*)-*endo*-spirosorbicillinol C (**26**, 2.10 mg, 4.30 µmol, 4%, brsm: 6%) were obtained as side products.

Synthesized Spirosorbicillinol C (**7**)

**ORD**: $[\alpha]_D$ = +109.1 (c = 0.17 in MeOH). **$^1$H-NMR** (600 MHz, MeOD): δ = 7.33 (dd, *J* = 15.1, 10.9 Hz, 1 H, sorbyl-C*H*), 6.57–6.56 (m, 1 H, C=C*H*), 6.40 (ddd, *J* = 14.8, 10.9, 1.6 Hz, 2 H, sorbyl-C*H*), 6.27–6.18 (m, 1 H, sorbyl-C*H*), 4.51–4.45 (m, 1 H, C*H*O), 4.32 (dd, *J* = 10.2, 8.4 Hz, 1 H, C*H*O), 4.02 (td, *J* = 9.7, 6.7 Hz, 1 H, C*H*O), 3.74 (s, 3 H, $CO_2CH_3$), 3.27 (dd, *J* = 3.5, 2.5 Hz, 1 H, C*H*), 3.15 (dd, *J* = 14.2, 2.5 Hz, 1 H, $CH_2$), 2.89 (ddt, *J* = 18.1, 6.7, 1.4 Hz, 1 H, $CH_2$), 2.27 (ddt, *J* = 18.2, 9.6, 3.4 Hz, 1 H, $CH_2$), 2.22 (dd, *J* = 14.1, 3.5 Hz, 1 H, $CH_2$), 1.89 (dd, *J* = 6.6, 1.4 Hz, 3 H, sorbyl-$CH_3$), 1.27 (s, 3 H, $CCH_3$), 1.19 (s, 3 H, $CCH_3$). **$^1$H-NMR** (300 MHz, MeOD): δ = 7.33 (dd, *J* = 14.9, 10.7 Hz, 1 H, sorbyl-C*H*), 6.60–6.54 (m, 1 H, C=C*H*), 6.45–6.32 (m, 2 H, sorbyl-C*H*), 6.30–6.16 (m, 1 H, sorbyl-C*H*), 4.53–4.42 (m, 1 H, C*H*O), 4.32 (dd, *J* = 10.1, 8.4 Hz, 1 H, C*H*O), 4.02 (td, *J* = 9.6, 6.6 Hz, 1 H, C*H*O), 3.74 (s, 3 H, $CO_2CH_3$), 3.27 (t, *J* = 2.7 Hz, 1 H, C*H*, overlap with MeOD signal), 3.15 (dd, *J* = 14.1, 2.5 Hz, 1 H, $CH_2$), 2.89 (dd, *J* = 17.9, 6.6 Hz, 1 H, $CH_2$), 2.33–2.19 (m, 2 H, $CH_2$), 1.89 (d, *J* = 6.6 Hz, 3 H, sorbyl-$CH_3$), 1.27 (s, 3 H, $CCH_3$), 1.19 (s, 3 H, $CCH_3$). **$^{13}$C-NMR** (150 MHz, MeOD): δ = 205.9, 195.7, 171.0, 168.3, 167.0, 143.8, 140.5, 133.8, 132.2, 130.8, 119.2, 111.0, 83.2, 82.9, 74.9, 73.5, 70.4, 67.1, 52.5, 40.7, 39.8, 33.9, 24.8, 18.8, 8.5. **HRMS** (ESI+) *m/z* calcd for $C_{25}H_{28}NaO_{10}$ $[M+Na]^+$: 511.1575; found: 511.1580.

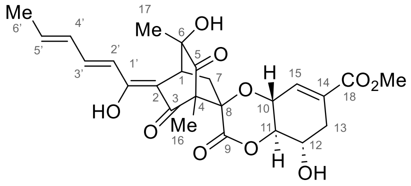

**Tab. S1** NMR data of synthesized spirosorbicillinol C (**7**).
$^{13}$C-shifts based on HSQC and HMBC signals. (MeOD)

| | δC | δH | COSY | HMBC (H→C) |
|---|---|---|---|---|
| 1 | 40.7 | 3.27 (dd, 3.5, 2.5 Hz) | 7 | 2, 3, 5, 6, 7, 8 |
| 2 | 111.0 | | | |
| 3 | 195.7 | | | |
| 4 | 70.4 | | | |
| 5 | 205.9 | | | |
| 6 | 73.5 | | | |
| 7 | 39.8 | 3.15 (dd, 14.2, 2.5 Hz),<br>2.22 (dd, 14.1, 3.5 Hz) | 1 | 1, 2, 9 |
| 8 | 83.2 | | | |
| 9 | 171.0 | | | |
| 10 | 74.9 | 4.51–4.45 (m) | 11 | 11 |
| 11 | 82.9 | 4.32 (dd, 10.2, 8.4 H) | 10, 12 | 10, 12, 13 |
| 12 | 67.1 | 4.02 (td, 9.7, 6.7 Hz) | 11, 13 | 10, 11, 13 |
| 13 | 33.9 | 2.89 (ddt, 18.1, 6.7, 1.4 Hz),<br>2.27 (ddt, 18.2, 9.6, 3.4 Hz) | 12, 15 | 11, 12, 14, 25 |
| 14 | 130.8 | | | |
| 15 | 133.8 | 6.57–6.56 (m) | 13 | 11, 13 |
| 16 | 8.5 | 1.27 (s) | | 3, 4, 5, 8 |
| 17 | 24.8 | 1.19 (s) | | 5, 6 |
| 18 | 167.0 | | | |
| 19 | 52.5 | 3.74 (s) | | 18 |
| 1’ | 168.3 | | | |
| 2’ | 119.2 | 6.40 (ddd, 14.8, 10.9, 1.6 Hz) | 3’ | 3’, 4’ |
| 3’ | 143.8 | 7.33 (dd, *J* = 15.1, 10.9 Hz) | 2’, 4’ | 1’, 2’, 4’, 5’ |
| 4’ | 132.2 | 6.40 (ddd, 14.8, 10.9, 1.6 Hz) | 3’, 5’ | 1’, 3’ |
| 5’ | 140.5 | 6.27–6.18 (m) | 4’ | 3’, 6’ |
| 6’ | 18.8 | 1.89 (dd, 6.6, 1.4 Hz) | 5’ | 2’, 3’, 4’, 5’ |

## (12*R*)-Spirosorbicillinol A (24) and (12*R*)-Spirosorbicillinol B (25)

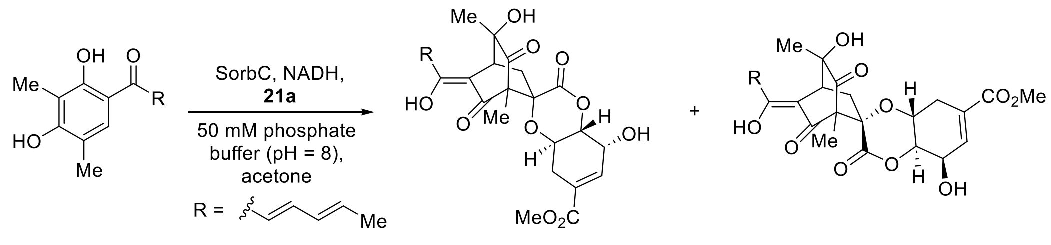

**24** **25**

The chemo-enzymatic reaction was performed with sorbicillin as substrate (50.2 mg, 216 µmol, 1.0 eq.), 460 mg NADH-$Na_2$ (648 µmol, 3.0 eq.), 20.0 mg SorbC (11.1 mg/mL stock solution, 1.80 mL, 0.42 µmol, 0.2 mol%) being dissolved in acetone (10.8 mL) and phosphate buffer (97.2 mL). The reaction was incubated at 4 °C and 150 rpm (GFL 3005) for 4 h before being extracted with DCM (3x300 mL). During workup, (8*R*)-scytolide (**21a**, 51.9 mg, 216 µmol, 1.0 eq.) was added and the solvent was evaporated under reduced pressure, to increase the concentration of dienophile slowly over time. The crude material was purified by semi-preparative HPLC (gradient, 5–95% ACN in 60 min) to give (12*R*)-spirosorbicllinol A (**24**, 12.6 mg, 25.8 µmol, 12%) and (12*R*)-spirosorbicillinol B (**25**, 36.8 mg, 75.3 µmol, 35%) as slightly yellow solids. The substrates were re-isolated in 26% yield (sorbicillin, 13.3 mg, 57.1 µmol) and 25% yield (**21a**, 13.0 mg, 54.1 µmol), based on which the desired products were obtained in 16% (**24**) and 47% (**25**) yield brsm.

(12*R*)-Spirosorbicillinol A (**24**)

**ORD**: $[\alpha]_D$ = +76.6 (c = 0.84 in MeOH). **$^1$H-NMR** (600 MHz, MeOD): δ = 7.33 (dd, *J* = 14.9, 11.0 Hz, 1 H, sorbyl-C*H*), 6.87–6.85 (m, 1 H, C=C*H*), 6.43 (d, *J* = 14.9 Hz, 1 H, sorbyl-C*H*), 6.41 (ddd, *J* = 14.9, 11.0, 1.7 Hz, 1 H, sorbyl-C*H*), 6.27–6.20 (m, 1 H, sorbyl-C*H*), 4.49–4.46 (m, 1 H, C*H*O), 4.44 (dd, *J* = 10.3, 4.1 Hz, 1 H, C*H*O), 4.19 (td, *J* = 9.8, 6.2 Hz, 1 H, C*H*O), 3.75 (s, 3 H, $CO_2CH_3$), 3.33 (dd, *J* = 3.6, 2.5 Hz, 1 H, C*H*), 3.04 (dd, *J* = 14.1, 2.5 Hz, 1 H, $CH_2$), 2.88 (ddd, *J* = 17.6, 6.2, 1.0 Hz, 1 H, $CH_2$), 2.24 (ddd, *J* = 9.6, 2.7, 1.0 Hz, 1 H, $CH_2$), 2.21 (dd, *J* = 14.2, 3.3 Hz, 1 H, $CH_2$), 1.89 (dd, *J* = 6.9, 1.6 Hz, 3 H, sorbyl-$CH_3$), 1.23 (s, 3 H, $CCH_3$), 1.21 (s, 3 H, $CCH_3$). **$^{13}$C-NMR** (150 MHz, MeOD): δ = 206.8, 195.7, 172.4, 168.3, 167.7, 143.8, 140.6, 136.2, 132.3, 131.6, 119.3, 111.2, 84.2, 82.7, 74.6, 69.4, 64.8, 64.5, 52.7, 41.4, 37.0, 31.2, 24.8, 18.9, 8.0. **HRMS** (ESI–) *m/z* calcd for $C_{25}H_{27}O_{10}$ $[M-H]^-$: 487.1610; found: 487.1609.

(12*R*)-Spirosorbicillinol B (**25**)

**ORD**: $[\alpha]_D$ = +276.8 (c = 0.43 in MeOH). **$^1$H-NMR** (600 MHz, MeOD): δ = 7.29 (dd, *J* = 14.9, 11.0 Hz, 1 H, sorbyl-C*H*), 6.89–6.82 (m, 1 H, C=C*H*), 6.50–6.29 (m, 2 H, sorbyl-C*H*), 6.25–6.16 (m, 1 H, sorbyl-C*H*), 4.52–4.47 (m, 2 H, C*H*O), 4.21 (ddt, *J* = 12.3, 9.6, 5.3 Hz, 1 H, C*H*O), 3.75 (s, 3 H, $CO_2CH_3$), 3.32–3.30 (m, 1H, C*H*, overlap with MeOD signal), 2.99–2.93 (m, 2 H, $CH_2$), 2.39–2.32 (m, 1 H, $CH_2$), 2.26 (ddd, *J* = 18.2, 9.6, 3.1 Hz, 1 H, $CH_2$), 1.88 (dd, *J* = 6.9, 3.1 Hz, 3 H, sorbyl-$CH_3$), 1.27 (s, 3 H, $CCH_3$), 1.20 (s, 3 H, $CCH_3$). **$^{13}$C-NMR** (150 MHz, MeOD): δ = 207.2, 196.3, 171.4, 167.8, 167.7, 143.1, 140.0, 135.9, 132.3, 131.5, 119.5, 111.5, 82.8, 81.1, 74.9, 70.9, 68.3, 64.8, 52.7, 41.2, 40.7, 32.3, 24.8, 18.9, 9.1. **HRMS** (ESI+) *m/z* calcd for $C_{25}H_{28}NaO_{10}$ $[M+Na]^+$: 511.1575; found: 511.1564.

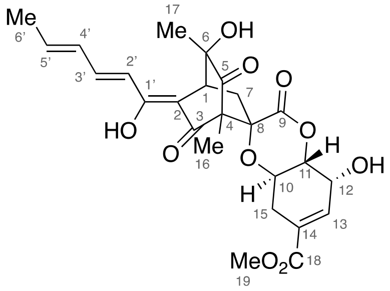


**Tab. S2** NMR data of (12*R*)-spirosorbicillinol A (**24**). (MeOD)

| | $\delta_C$ | $\delta_H$ | COSY | HMBC (H→C) | NOESY |
|---|---|---|---|---|---|
| 1 | 41.4 | 3.33 (dd, 3.6, 2.5 Hz) | 7 | 3, 5, 6, 8, 1′ | 17, 2′ |
| 2 | 111.2 | | | | |
| 3 | 195.7 | | | | |
| 4 | 69.4 | | | | |
| 5 | 206.8 | | | | |
| 6 | 74.6 | | | | |
| 7 | 37.0 | 3.04 (dd, 14.1, 2.5 Hz),<br>2.21 (dd, 14.2, 3.3 Hz) | 1 | 1, 2, 4, 6, 8, 9 | 10 |
| 8 | 84.2 | | | | |
| 9 | 172.4 | | | | |
| 10 | 64.5 | 4.19 (td, 9.8, 6.2 Hz) | 11, 15 | 11, 15 | 7 |
| 11 | 82.7 | 4.44 (dd, 10.3, 4.1 Hz) | 10 | 12, 15 | 13 |
| 12 | 64.8 | 4.49–4.46 (m) | 13 | 10, 11, 13, 14 | |
| 13 | 136.2 | 6.87–6.85 (m) | 12 | 11, 15, 18 | 11 |
| 14 | 131.6 | | | | |
| 15 | 31.2 | 2.88 (ddd, 17.6, 6.2, 1.0 Hz),<br>2.24 (ddd, 17.6, 2.7, 1.0 Hz) | 10 | 11, 12, 13, 14, 18 | |
| 16 | 8.0 | 1.23 (s) | | 3, 4, 5, 8 | |
| 17 | 24.8 | 1.21 (s) | | 1, 5, 6 | 1 |
| 18 | 167.7 | | | | |
| 19 | 52.7 | 3.75 (s) | | 18 | |
| 1′ | 168.3 | | | | |
| 2′ | 119.3 | 6.43 (d, 14.9 Hz) | 3′ | 3′, 4′ | 1 |
| 3′ | 143.8 | 7.33 (dd, 14.9, 11.0 Hz) | 2′, 4′ | 1′, 2′, 4′, 5′ | |
| 4′ | 132.3 | 6.41 (ddd, 14.9, 11.0, 1.7 Hz) | 3′, 5′ | 1′, 3′ | |
| 5′ | 140.6 | 6.27–6.20 (m) | 4′ | 3′, 6′ | |
| 6′ | 18.9 | 1.89 (dd, 6.9, 1.6 Hz) | 5′ | 2′, 3′, 4′, 5′ | |

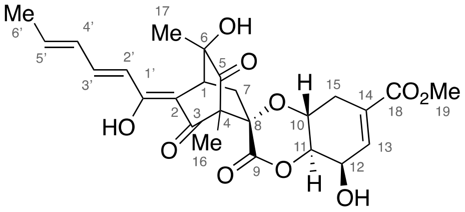


**Tab. S3** NMR data of (12*R*)-spirosorbicillinol B (**25**). (MeOD)

| | $\delta_C$ | $\delta_H$ | COSY | HMBC (H→C) | NOESY |
|---|---|---|---|---|---|
| 1 | 41.2 | 3.32–3.30 (m) | 7 | 2, 3, 5, 6, 8, 1′ | 7, 17 |
| 2 | 111.5 | | | | |
| 3 | 196.3 | | | | |
| 4 | 70.9 | | | | |
| 5 | 207.2 | | | | |
| 6 | 74.9 | | | | |
| 7 | 40.7 | 2.99–2.93 (m),<br>2.39–2.32 (m) | 1 | 1, 2, 4, 6, 8, 9, 1′ | 1, 10, 15 |
| 8 | 82.8 | | | | |
| 9 | 171.4 | | | | |
| 10 | 68.3 | 4.21 (ddt, 12.3, 9.6, 5.3 Hz) | 15 | 11 | 7, 16 |
| 11 | 81.1 | 4.52–4.47 (m) | 12 | 13, 15 | 13 |
| 12 | 64.8 | 4.52–4.47 (m) | 11, 13 | 10, 11, 14 | |
| 13 | 135.9 | 6.89–6.82 (m) | 12, 15 | 11, 12, 15, 18 | 11 |
| 14 | 131.5 | | | | |
| 15 | 32.3 | 2.99–2.93 (m),<br>2.26 (ddd, 18.2, 9.6, 3.1 Hz) | 10, 13 | 10, 11, 13, 14, 18 | 7 |
| 16 | 9.1 | 1.27 (s) | | 3, 4, 5, 8 | 10 |
| 17 | 24.8 | 1.20 (s) | | 1, 5, 6 | 1 |
| 18 | 167.7 | | | | |
| 19 | 52.7 | 3.75 (s) | | 14, 18 | |
| 1′ | 167.8 | | | | |
| 2′ | 119.3 | 6.50–6.29 (m) | 3′ | 3′, 4′ | |
| 3′ | 143.1 | 7.29 (dd, 14.9, 11.0 Hz) | 2′, 4′ | 1′, 4′, 5′ | 5′ |
| 4′ | 132.3 | 6.50–6.29 (m) | 3′, 5′ | 1′, 3′ | 6′ |
| 5′ | 140.0 | 6.25–6.16 (m) | 4′ | 3′, 6′ | 3′, 6′ |
| 6′ | 18.9 | 1.88 (dd, 6.9, 3.1 Hz) | 5′ | 3′, 4′, 5′ | 4′, 5′ |

**(12*R*)-Spirosorbicillinol C (26)**

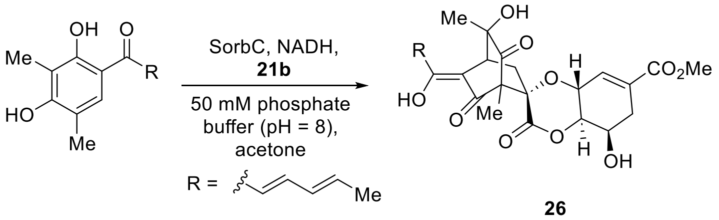


The chemo-enzymatic reaction was performed with sorbicillin as substrate (25.1 mg, 108 µmol, 1.0 eq.), 230 mg NADH-$Na_2$ (324 µmol, 3.0 eq.), 10.0 mg SorbC (11.1 mg/mL stock solution, 0.91 mL, 0.21 µmol, 0.2 mol%) being dissolved in acetone (5.4 mL) and phosphate buffer (48.6 mL). The reaction was incubated at 4 °C and 150 rpm (GFL 3005) for 4 h before being extracted with DCM (3x200 mL). During workup, (8*R*)-*epi*-scytolide (**21b**, 26.0 mg, 108 µmol, 1.0 eq.) was added and the solvent was evaporated under reduced pressure, to increase the concentration of dienophile slowly over time. The crude material was purified by semi-preparative HPLC (gradient, 5–95% ACN in 60 min) to give (12*R*)-spirosorbicllinol C (9.00 mg, 18.4 µmol, 17%) slightly yellow oil. The substrate was re-isolated in 28% yield (7.00 mg, 30.1 µmol), based on which the desired product was obtained in 24% (**26**) yield brsm.

**ORD**: $[\alpha]_D$ = +455.6 (c = 0.46 in MeOH). **$^1$H-NMR** (600 MHz, MeOD): δ = 7.34 (dd, *J* = 14.9, 11.0 Hz, 1 H, sorbyl-C*H*), 6.85 (dd, *J* = 2.6, 1.1 Hz, 1 H, C=C*H*), 6.44 (d, *J* = 15.0 Hz, 1 H, sorbyl-C*H*), 6.43 (ddd, *J* = 15.0, 11.0, 1.1 Hz, 1 H, sorbyl-C*H*), 6.29–6.20 (m, 1 H, sorbyl-C*H*), 4.93–4.90 (m, 1 H, C*H*O), 4.88–4.85 (m, 1 H, C*H*O, overlap with MeOD signal), 3.94 (ddd, *J* = 10.2, 6.0, 1.6 Hz, 1 H, C*H*O), 3.77 (s, 3 H, $CO_2CH_3$), 3.28 (t, *J* = 3.0 Hz, 1 H, C*H*), 2.98 (dd, *J* = 14.3, 2.4 Hz, 1 H, $CH_2$), 2.73 (ddt, *J* = 17.5, 5.9, 0.8 Hz, 1 H, $CH_2$), 2.30 (ddt, *J* = 17.3, 10.0, 3.2 Hz, 1 H, $CH_2$), 2.11 (dd, *J* = 14.3, 3.6 Hz, 1 H, $CH_2$), 1.90 (dd, *J* = 7.0, 1.6 Hz, 3 H, sorbyl-$CH_3$), 1.24 (s, 3 H, $CCH_3$), 1.17 (s, 3 H, $CCH_3$). **$^{13}$C-NMR** (150 MHz, MeOD): δ = 207.0, 195.3, 172.2, 168.4, 167.1, 143.9, 140.7, 134.7, 133.6, 132.3, 119.3, 111.4, 82.2, 81.0, 74.5, 70.2, 69.2, 67.6, 52.8, 41.6, 40.7, 29.2, 24.6, 18.9, 8.1. **HRMS** (ESI+) *m/z* calcd for $C_{25}H_{28}NaO_{10}$ $[M+Na]^+$: 511.1575; found: 511.1579.

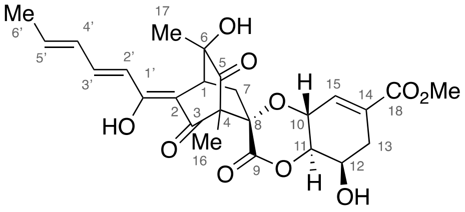


**Tab. S4** NMR data of (12*R*)-spirosorbicillinol C (**26**). (MeOD)

| | $\delta_C$ | $\delta_H$ | COSY | HMBC (H→C) | NOESY |
|---|---|---|---|---|---|
| 1 | 41.6 | 3.28 (t, 3.0 Hz) | 7 | 2, 3, 5, 6, 8, 17, 1′ | 17, 2′ |
| 2 | 111.4 | | | | |
| 3 | 195.3 | | | | |
| 4 | 70.2 | | | | |
| 5 | 207.0 | | | | |
| 6 | 74.5 | | | | |
| 7 | 40.7 | 2.98 (dd, 14.3, 2.4 Hz),<br>2.11 (dd, 14.3, 3.6 Hz) | 1 | 1, 2, 4, 6, 9 | 15 |
| 8 | 82.2 | | | | |
| 9 | 172.2 | | | | |
| 10 | 69.2 | 4.88–4.85 (m) | 11, 15 | | |
| 11 | 81.0 | 4.93–4.90 (m) | 10, 12 | 13, 15 | 12, 16 |
| 12 | 67.6 | 3.94 (ddd, 10.2, 6.0, 1.6 Hz) | 11, 13 | 10 | 11, 13 |
| 13 | 29.2 | 2.73 (ddt, 17.5, 5.9, 0.8 Hz),<br>2.30 (ddt, 17.3, 10.0, 3.2 Hz) | 12, 15 | 11, 15, 18 | 12 |
| 14 | 133.6 | | | | |
| 15 | 134.7 | 6.85 (dd, 2.6, 1.1 Hz) | 10, 13 | 11, 13 | 7 |
| 16 | 8.1 | 1.24 (s) | | 3, 4, 5, 8 | 11 |
| 17 | 24.6 | 1.17 (s) | | 1, 5, 6 | 1 |
| 18 | 167.1 | | | | |
| 19 | 52.8 | 3.77 (s) | | 18 | |
| 1′ | 168.4 | | | | |
| 2′ | 119.3 | 6.44 (d, 15.0 Hz) | 3′ | 3′, 4′ | 1 |
| 3′ | 143.9 | 7.34 (dd, 14.9, 11.0 Hz) | 2′, 4′ | 1′, 4′, 5′ | |
| 4′ | 132.3 | 6.43 (ddd, 15.0, 11.0, 1.1 Hz) | 3′, 5′ | 1′, 3′ | |
| 5′ | 140.7 | 6.29–6.20 (m) | 4′ | 3′, 6′ | |
| 6′ | 18.9 | 1.88 (dd, 6.9, 3.1 Hz) | 5′ | 3′, 4′, 5′ | |

## 2.4 NMR Comparison with Literature

### Spirosorbicillinol A (5) and (12*R*)-Spirosorbicillinol A (24)

**Tab. S5** NMR comparison of spirosorbicillinol A with literature and (12*R*)-isomer.

| | literature of **5**[9] | | synthesized **5** | (12*R*)-isomer (**24**) | |
|---|---|---|---|---|---|
| | $\delta_H$ | $\delta_C$ | $\delta_H$ | $\delta_H$ | $\delta_C$ |
| 1 | 3.31 (overlapped) | 41.5 | 3.32–3.30 (overlapped) | 3.33 (dd, 3.6, 2.5 Hz) | 41.4 |
| 2 | | 111.2 | | | 111.2 |
| 3 | | 195.6 | | | 195.7 |
| 4 | | 69.4 | | | 69.4 |
| 5 | | 206.8 | | | 206.8 |
| 6 | | 74.7 | | | 74.6 |
| 7 | 3.05 (dd, 14.8, 3.0 Hz),<br>2.18 (dd, 14.8, 3.0 Hz) | 37.3 | 3.05 (dd, 14.1, 2.4 Hz),<br>2.18 (dd, 14.1, 3.6 Hz) | 3.04 (dd, 14.1, 2.5 Hz),<br>2.21 (dd, 14.2, 3.3 Hz) | 37.0 |
| 8 | | 84.4 | | | 84.2 |
| 9 | | 172.1 | | | 172.4 |
| 10 | 4.07 (dt, 10.0, 6.0 Hz) | 70.7 | 4.08 (td, 9.9, 6.2 Hz) | 4.19 (td, 9.8, 6.2 Hz) | 64.5 |
| 11 | 4.27 (dd, 10.0, 8.4 Hz) | 86.0 | 4.27 (dd, 10.0, 8.1 Hz) | 4.44 (dd, 10.3, 4.1 Hz) | 82.7 |
| 12 | 4.45 (m) | 67.2 | 4.50–4.40 (m) | 4.49–4.46 (m) | 64.8 |
| 13 | 6.65 (dd, 3.2, 1.6 Hz) | 139.6 | 6.65 (dd, 2.4, 2.1 Hz) | 6.87–6.85 (m) | 136.2 |
| 14 | | 129.0 | | | 131.6 |
| 15 | 2.80 (dd, 17.6, 6.0 Hz),<br>2.25 (m) | 30.6 | 2.80 (dd, 17.2, 6.3 Hz),<br>2.31–2.22 (m) | 2.88 (ddd, 17.6, 6.2, 1.0 Hz),<br>2.24 (ddd, 17.6, 2.7, 1.0 Hz) | 31.2 |
| 16 | 1.23 (s) | 8.1 | 1.23 (s) | 1.23 (s) | 8.0 |
| 17 | 1.20 (s) | 24.9 | 1.20 (s) | 1.21 (s) | 24.8 |
| 18 | | 167.5 | | | 167.7 |
| 19 | 3.74 (s) | 52.7 | 3.74 (s) | 3.75 (s) | 52.7 |
| 1’ | | 168.4 | | | 168.3 |
| 2’ | 6.41 (d, 14.4 Hz) | 119.3 | 6.48–6.35 (m) | 6.43 (d, 14.9 Hz) | 119.3 |
| 3’ | 7.33 (dd, 14.4, 10.8 Hz) | 143.8 | 7.33 (dd, 14.9, 10.7 Hz) | 7.33 (dd, 14.9, 11.0 Hz) | 143.8 |
| 4’ | 6.40 (m) | 132.3 | 6.48–6.35 (m) | 6.41 (ddd, 14.9, 11.0, 1.7 Hz) | 132.3 |
| 5’ | 6.23 (m) | 140.6 | 6.30–6.16 (m) | 6.27–6.20 (m) | 140.6 |
| 6’ | 1.89 (d, 1.89 Hz) | 18.9 | 1.89 (d, 6.7 Hz) | 1.89 (dd, 6.9, 1.6 Hz) | 18.9 |

## Spirosorbicillinol B (6) and (12*R*)-Spirosorbicillinol B (25)

**Tab. S6** NMR comparison of spirosorbicillinol B with literature and (12*R*)-isomer.

| | literature of **6**[9] | | synthesized **6** | (12*R*)-isomer (**25**) | |
|---|---|---|---|---|---|
| | $\delta_H$ | $\delta_C$ | $\delta_H$ | $\delta_H$ | $\delta_C$ |
| 1 | 3.30 (overlapped) | 41.3 | 3.32–3.30 (overlapped) | 3.32–3.30 (overlapped) | 41.2 |
| 2 | | 111.5 | | | 111.5 |
| 3 | | 196.3 | | | 196.3 |
| 4 | | 71.0 | | | 70.9 |
| 5 | | 207.2 | | | 207.2 |
| 6 | | 75.0 | | | 74.9 |
| 7 | 2.98 (dd, 14.0, 2.8 Hz),<br>2.33 (dd, 14.0, 2.8 Hz) | 40.5 | 2.98 (dd, 14.0, 3.0 Hz),<br>2.38–2.23 (m) | 2.99–2.93 (m),<br>2.39–2.32 (m) | 40.7 |
| 8 | | 82.7 | | | 82.8 |
| 9 | | 171.1 | | | 171.4 |
| 10 | 4.01 (dt, 10.0, 6.8 Hz) | 70.7 | 4.01 (td, 9.8, 6.3 Hz) | 4.21 (ddt, 12.3, 9.6, 5.3 Hz) | 68.3 |
| 11 | 4.36 (dd, 10.0, 7.6 Hz) | 83.8 | 4.37 (dd, 10.0, 7.6 Hz) | 4.52–4.47 (m) | 81.1 |
| 12 | 4.45 (m) | 71.4 | 4.51–4.41 (m) | 4.52–4.47 (m) | 64.8 |
| 13 | 6.64 (dd, 3.2, 2.0 Hz) | 139.3 | 6.65 (dd, 2.9, 2.2 Hz) | 6.89–6.82 (m) | 135.9 |
| 14 | | 128.1 | | | 131.5 |
| 15 | 2.90 (dd, 17.6, 6.8 Hz),<br>2.29 (m) | 31.5 | 2.89 (dd, 17.6, 5.9 Hz),<br>2.38–2.23 (m) | 2.99–2.93 (m),<br>2.26 (ddd, 18.2, 9.6, 3.1 Hz) | 32.3 |
| 16 | 1.25 (s) | 8.8 | 1.25 (s) | 1.27 (s) | 9.1 |
| 17 | 1.19 (s) | 24.9 | 1.19 (s) | 1.20 (s) | 24.8 |
| 18 | | 167.5 | | | 167.7 |
| 19 | 3.75 (s) | 52.7 | 3.75 (s) | 3.75 (s) | 52.7 |
| 1’ | | 168.0 | | | 167.8 |
| 2’ | 6.36 (d, 15.2 Hz) | 119.6 | 6.46–6.32 (m) | 6.50–6.29 (m) | 119.3 |
| 3’ | 7.29 (dd, 15.2, 10.8 Hz) | 143.3 | 7.30 (dd, 14.9, 10.9 Hz) | 7.29 (dd, 14.9, 11.0 Hz) | 143.1 |
| 4’ | 6.38 (m) | 132.4 | 6.46–6.32 (m) | 6.50–6.29 (m) | 132.3 |
| 5’ | 6.20 (m) | 140.1 | 6.28–6.12 (m) | 6.25–6.16 (m) | 140.0 |
| 6’ | 1.88 (d, 6.8 Hz) | 18.9 | 1.88 (6.8, 1.4 Hz) | 1.88 (dd, 6.9, 3.1 Hz) | 18.9 |

## Spirosorbicillinol C (7), synthesized Spirosorbicillinol C (7) and (12*R*)-Spirosorbicillinol C (26)

**Tab. S7** NMR comparison of synthesized spirosorbicillinol C with literature and (12*R*)-isomer.

| | literature of **7**[9] | | synthesized **7** | | (12*R*)-isomer (**26**) | |
|---|---|---|---|---|---|---|
| | $\delta_H$ | $\delta_C$ | $\delta_H$ | $\delta_C$ | $\delta_H$ | $\delta_C$ |
| 1 | 3.31 (overlapped) | 41.4 | 3.27 (dd, 3.5, 2.5 Hz) | 40.7 | 3.28 (t, 3.0 Hz) | 41.6 |
| 2 | | 111.4 | | 111.0 | | 111.4 |
| 3 | | 196.2 | | 195.7 | | 195.3 |
| 4 | | 71.0 | | 70.4 | | 70.2 |
| 5 | | 207.2 | | 205.9 | | 207.0 |
| 6 | | 75.1 | | 73.5 | | 74.5 |
| 7 | 2.99 (dd, 14.8, 3.2 Hz),<br>2.34 (dd, 14.8, 3.2 Hz) | 40.4 | 3.15 (dd, 14.2, 2.5 Hz),<br>2.22 (dd, 14.1, 3.5 Hz) | 39.8 | 2.98 (dd, 14.3, 2.4 Hz),<br>2.11 (dd, 14.3, 3.6 Hz) | 40.7 |
| 8 | | 83.9 | | 83.2 | | 82.2 |
| 9 | | 171.4 | | 171.0 | | 172.2 |
| 10 | 4.47 (m) | 75.1 | 4.51–4.45 (m) | 74.9 | 4.88–4.85 (m) | 69.2 |
| 11 | 4.31 (dd, 10.8, 9.6 Hz) | 83.0 | 4.32 (dd, 10.2, 8.4 H) | 82.9 | 4.93–4.90 (m) | 81.0 |
| 12 | 4.03 (dt, 9.6, 6.8 Hz) | 67.3 | 4.02 (td, 9.7, 6.7 Hz) | 67.1 | 3.94 (ddd, 10.2, 6.0, 1.6 Hz) | 67.6 |
| 13 | 2.90 (dd, 17.6, 6.8 Hz),<br>2.27 (m) | 34.2 | 2.89 (ddt, 18.1, 6.7, 1.4 Hz),<br>2.27 (ddt, 18.2, 9.6, 3.4 Hz) | 33.9 | 2.73 (ddt, 17.5, 5.9, 0.8 Hz),<br>2.30 (ddt, 17.3, 10.0, 3.2 Hz) | 29.2 |
| 14 | | 131.0 | | 130.8 | | 133.6 |
| 15 | 6.63 (s) | 134.3 | 6.57–6.56 (m) | 133.8 | 6.85 (dd, 2.6, 1.1 Hz) | 134.7 |
| 16 | 1.26 (s) | 8.6 | 1.27 (s) | 8.5 | 1.24 (s) | 8.1 |
| 17 | 1.20 (s) | 24.9 | 1.19 (s) | 24.8 | 1.17 (s) | 24.6 |
| 18 | | 167.5 | | 167.0 | | 167.1 |
| 19 | 3.75 (s) | 52.7 | 3.74 (s) | 52.5 | 3.77 (s) | 52.8 |
| 1′ | | 168.1 | | 168.3 | | 168.4 |
| 2′ | 6.36 (d, 14.8 Hz) | 119.5 | 6.40 (ddd, 14.8, 10.9, 1.6 Hz) | 119.2 | 6.44 (d, 15.0 Hz) | 119.3 |
| 3′ | 7.29 (dd, 14.8, 10.8 Hz) | 143.4 | 7.33 (dd, *J* = 15.1, 10.9 Hz) | 143.8 | 7.34 (dd, 14.9, 11.0 Hz) | 143.9 |
| 4′ | 6.37 (m) | 132.4 | 6.40 (ddd, 14.8, 10.9, 1.6 Hz) | 132.2 | 6.43 (ddd, 15.0, 11.0, 1.1 Hz) | 132.3 |
| 5′ | 6.20 (m) | 140.2 | 6.27–6.18 (m) | 140.5 | 6.29–6.20 (m) | 140.7 |
| 6′ | 1.88 (6.8 Hz) | 18.9 | 1.89 (dd, 6.6, 1.4 Hz) | 18.8 | 1.88 (dd, 6.9, 3.1 Hz) | 18.9 |

# 3. NMR Data

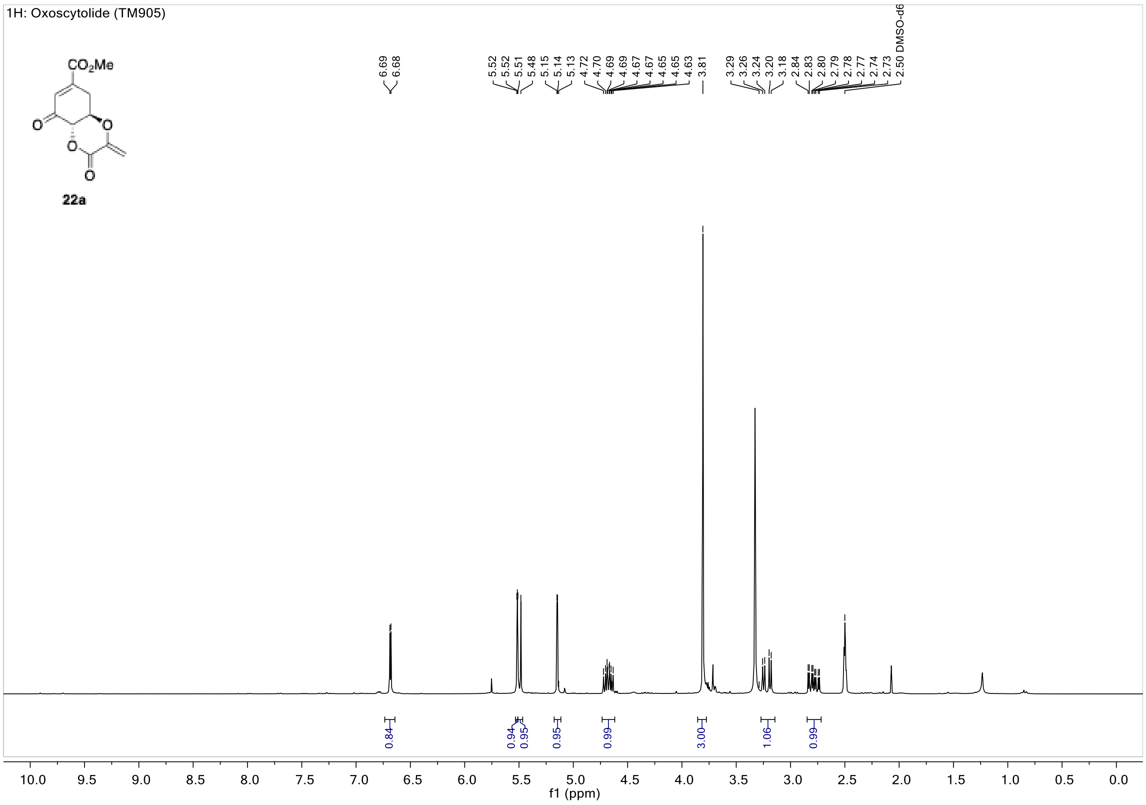

**Fig. S1** $^1$H-NMR spectrum of oxoscytolide (**22a**), measured in DMSO-$d_6$ at 300 MHz.

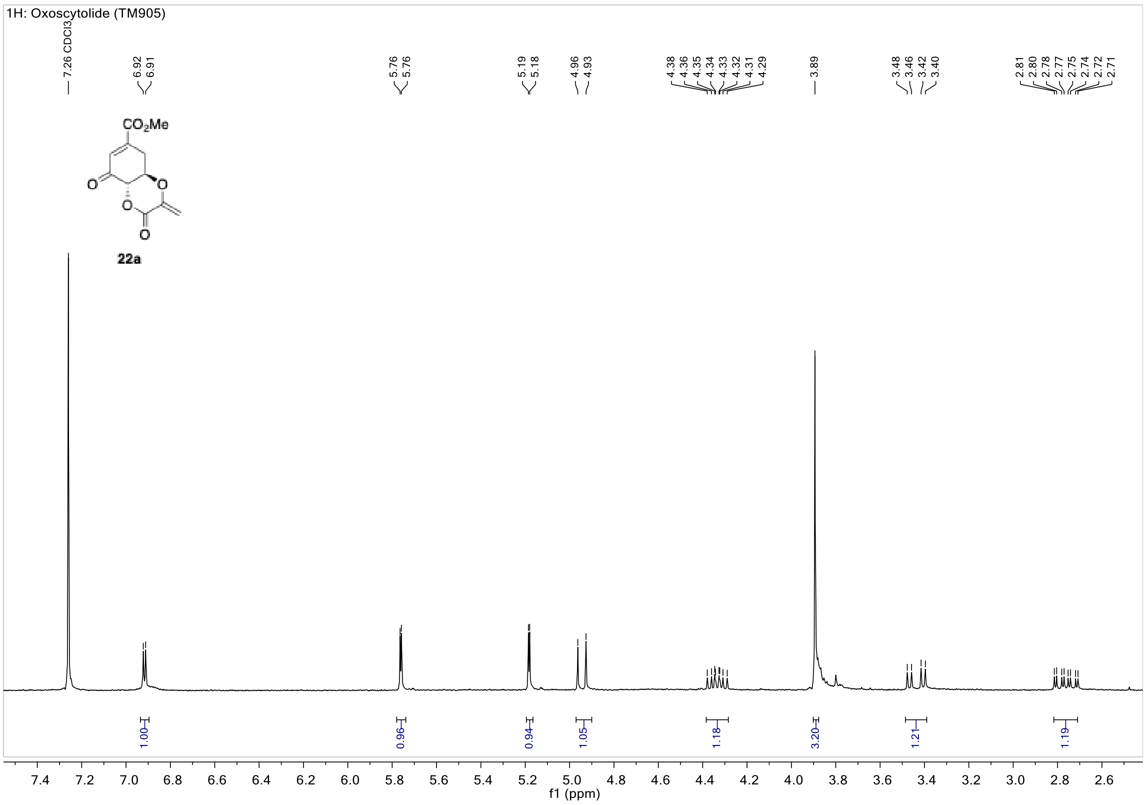

**Fig. S2** $^1$H-NMR spectrum of oxoscytolide (**22a**), measured in $CDCl_3$ at 300 MHz.

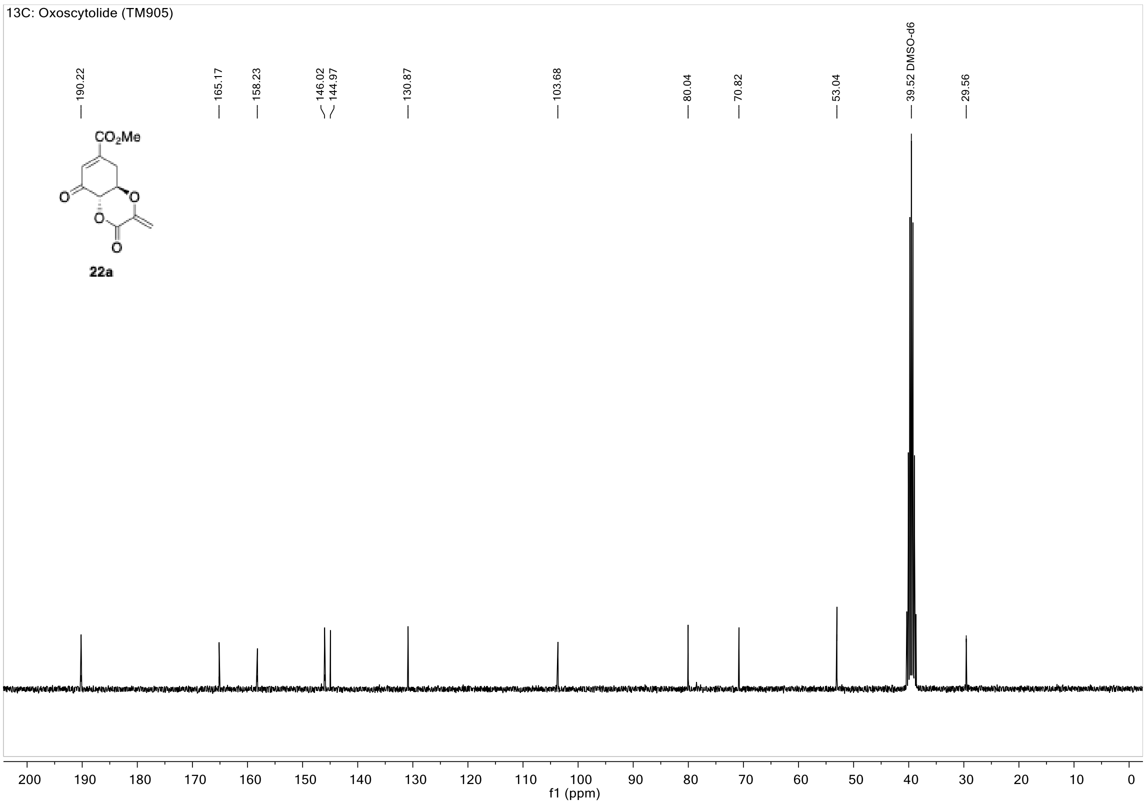


**Fig. S3** $^{13}$C-NMR spectrum of oxoscytolide (**22a**), measured in DMSO-d$_6$ at 75 MHz.

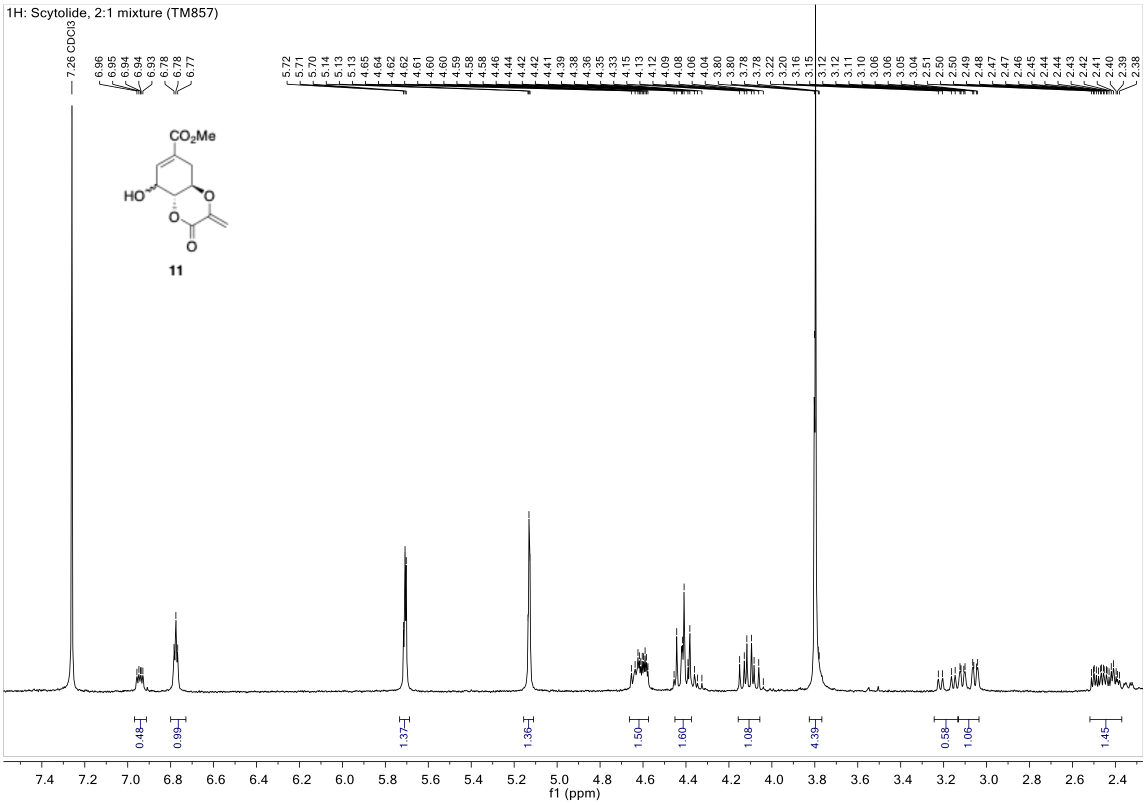

**Fig. S4** $^1$H-NMR spectrum of scytolide (**11**) and (8R)-scytolide (**21a**) (2:1 mixture), measured in $CDCl_3$ at 300 MHz.

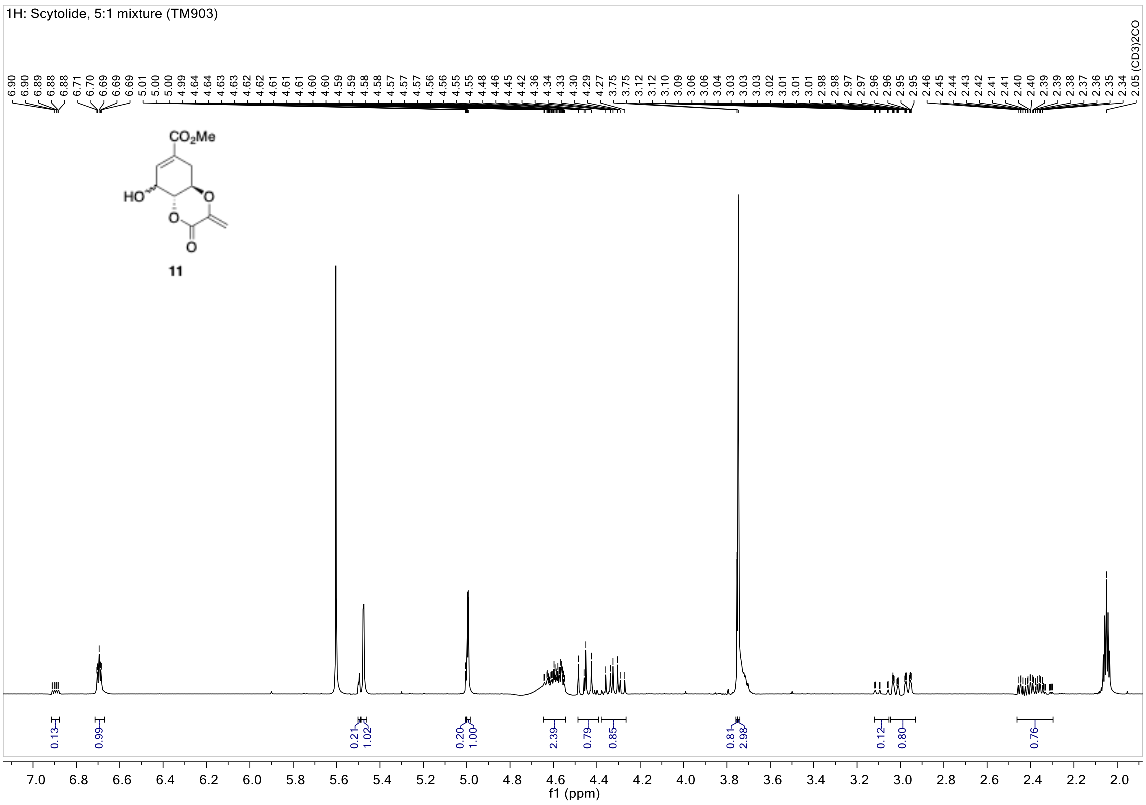

**Fig. S5** $^1$H-NMR spectrum of scytolide (**11**) and (8R)-scytolide (**21a**) (5:1 mixture), measured in acetone-$d_6$ at 300 MHz.

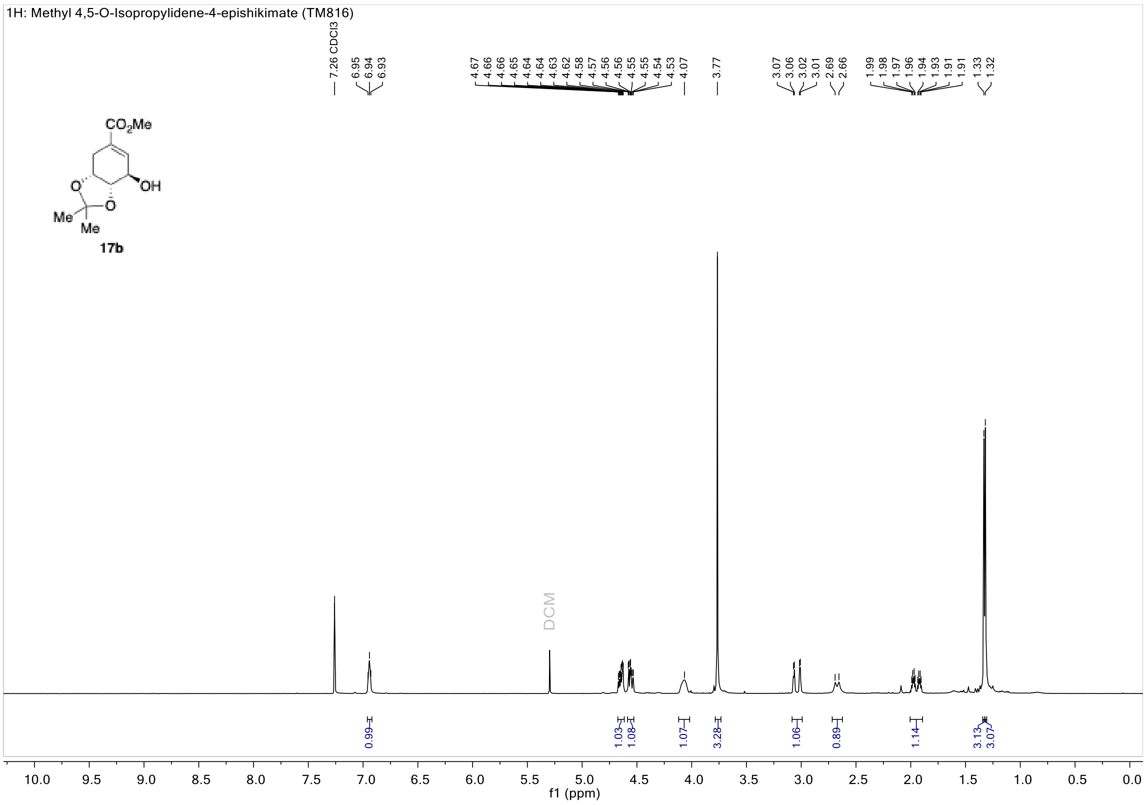


**Fig. S6** $^{1}$H-NMR spectrum of methyl 4,5-isopropylidene-4-epi-shikimate (**17b**) measured in $CDCl_3$ at 300 MHz.

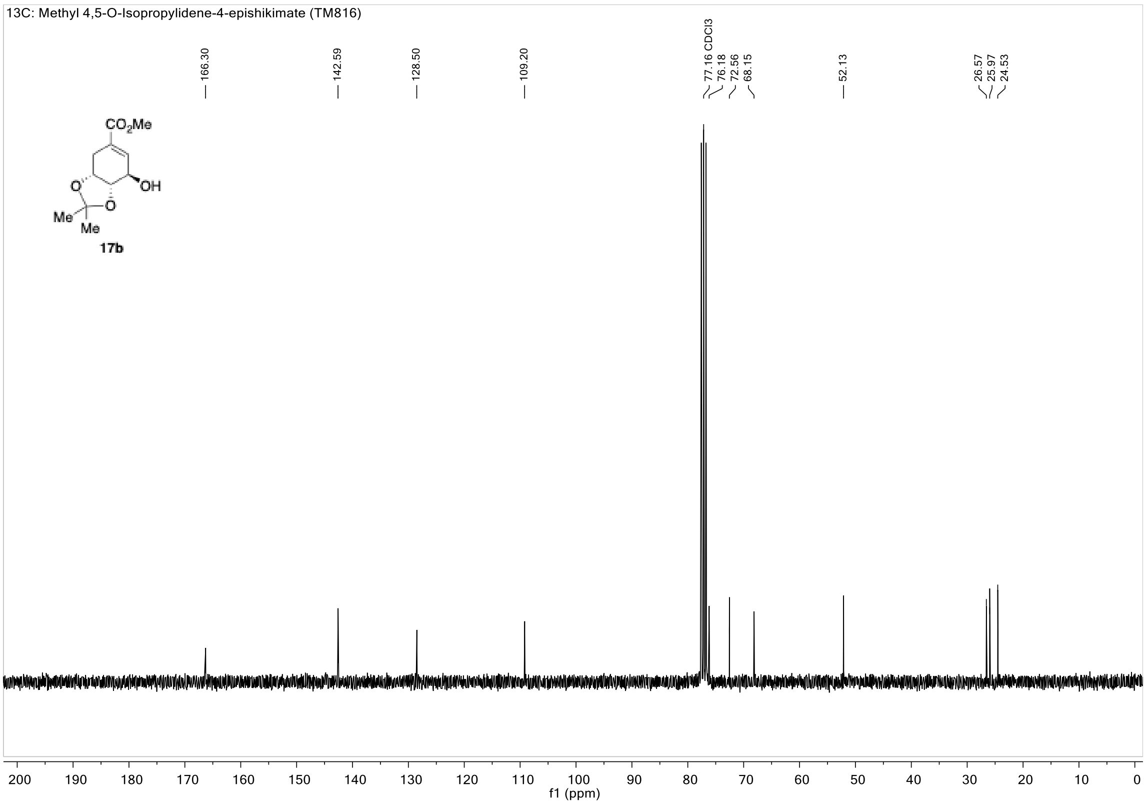


**Fig. S7** $^{13}$C-NMR spectrum of methyl 4,5-isopropylidene-4-epi-shikimate (**17b**) measured in $CDCl_3$ at 75 MHz.

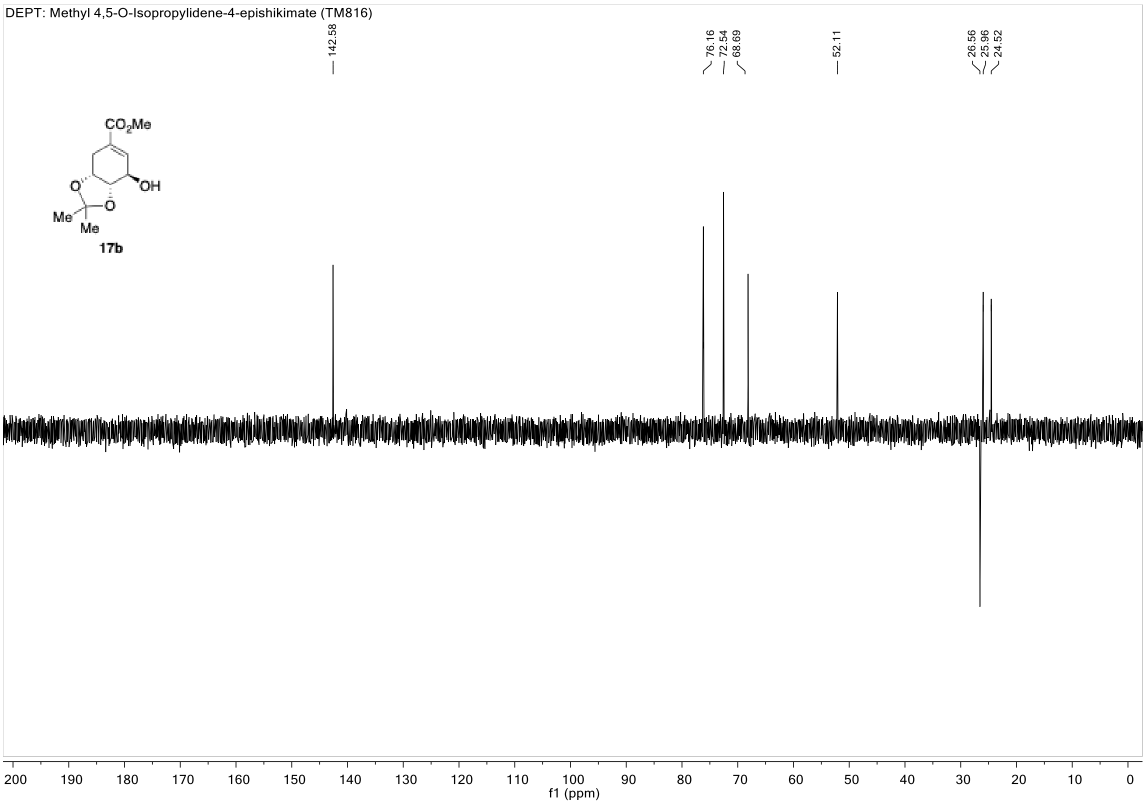


**Fig. S8** DEPT spectrum of methyl 4,5-isopropylidene-4-epi-shikimate (**17b**) measured in $CDCl_3$ at 75 MHz.

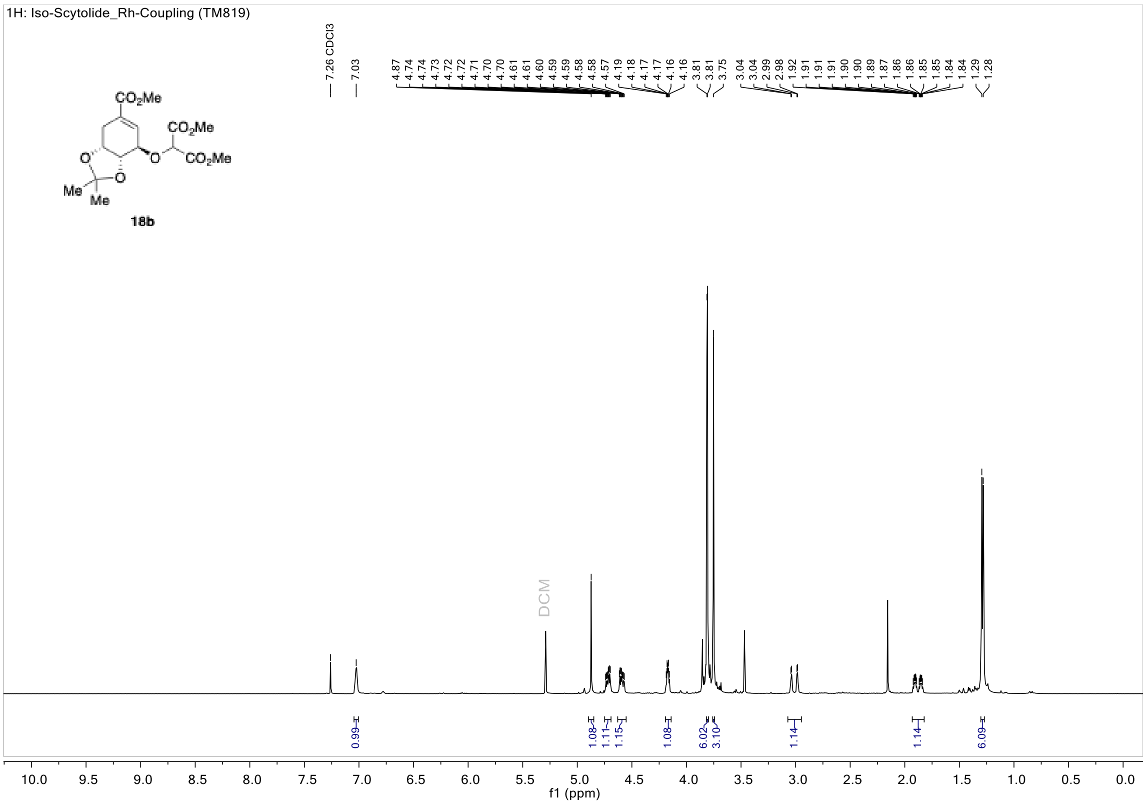


**Fig. S9** $^{1}$H-NMR spectrum of dimethyl 2-(((3a*R*,4*R*,7a*R*)-6-(methoxycarbonyl)-2,2-dimethyl-3a,4,7,7a-tetrahydrobenzo [*d*][1,3]dioxol-4-yl)oxy)malonate (**18b**) measured in $CDCl_3$ at 300 MHz.

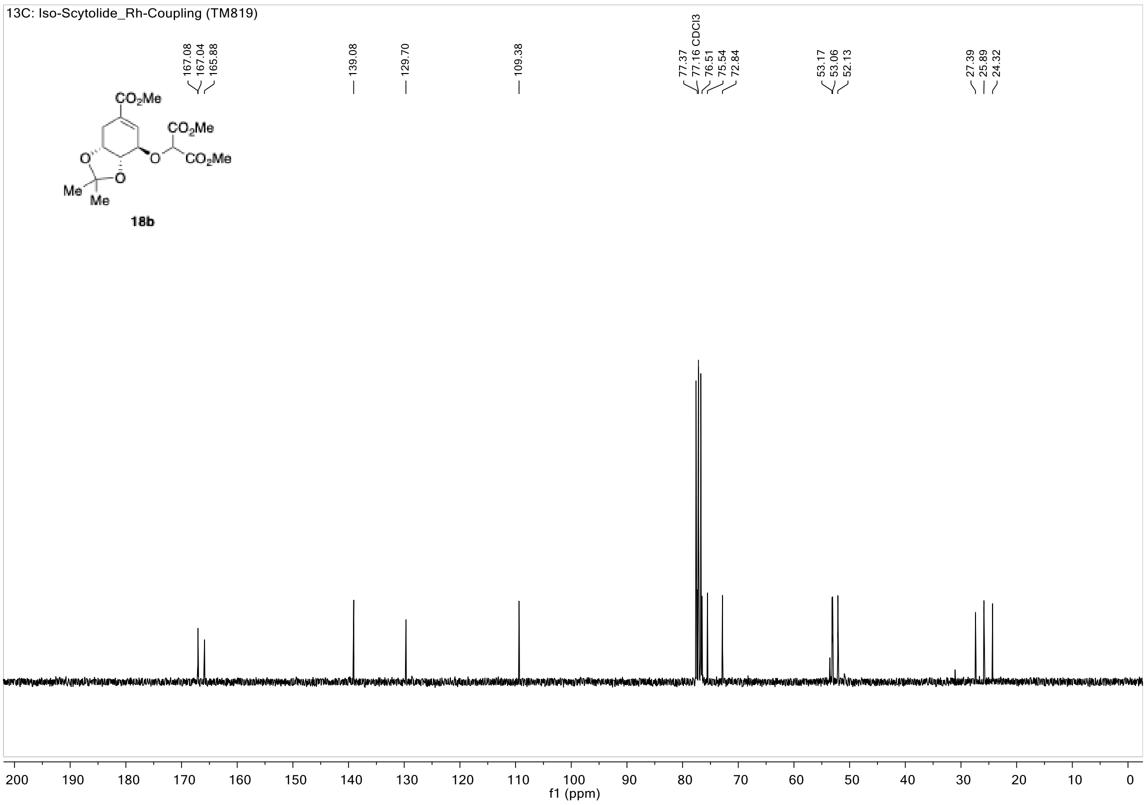


**Fig. S10** $^{13}$C-NMR spectrum of dimethyl 2-(((3a*R*,4*R*,7a*R*)-6-(methoxycarbonyl)-2,2-dimethyl-3a,4,7,7a-tetrahydrobenzo [*d*][1,3]dioxol-4-yl)oxy)malonate (**18b**) measured in $CDCl_3$ at 75 MHz.

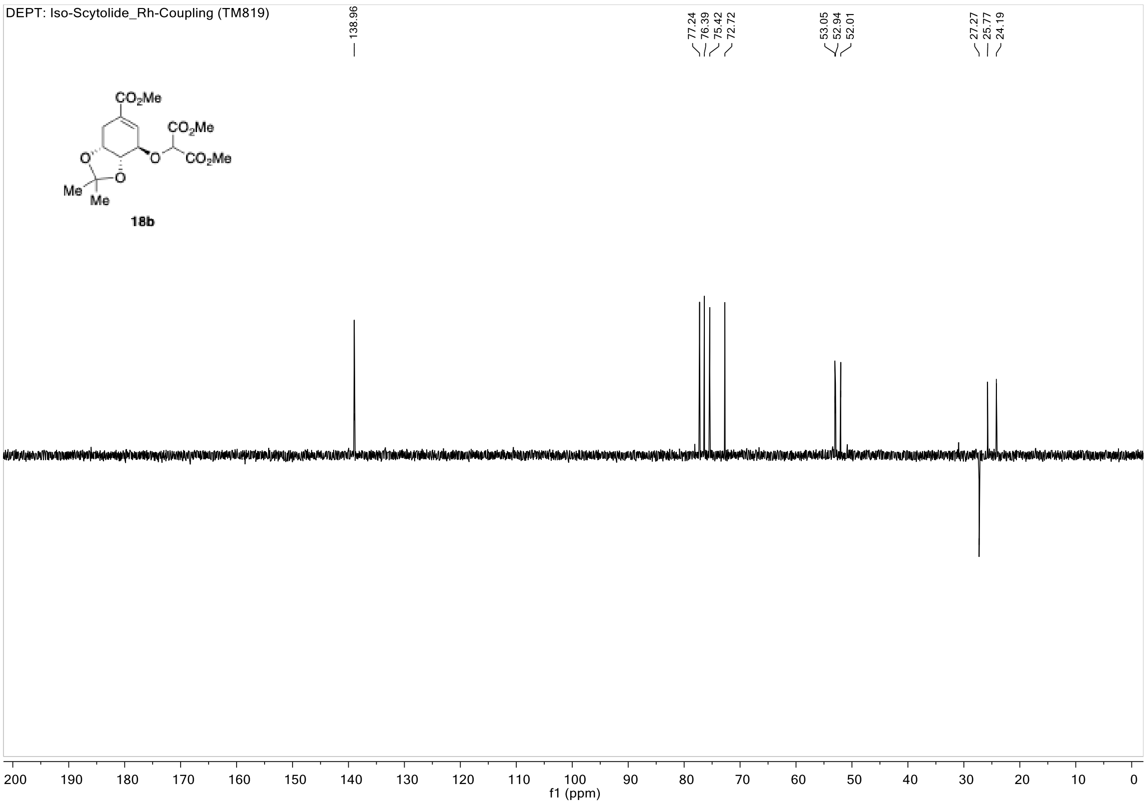


**Fig. S11** DEPT spectrum of dimethyl 2-(((3a*R*,4*R*,7a*R*)-6-(methoxycarbonyl)-2,2-dimethyl-3a,4,7,7a-tetrahydrobenzo [*d*][1,3]dioxol-4-yl)oxy)malonate (**18b**) measured in $CDCl_3$ at 75 MHz.

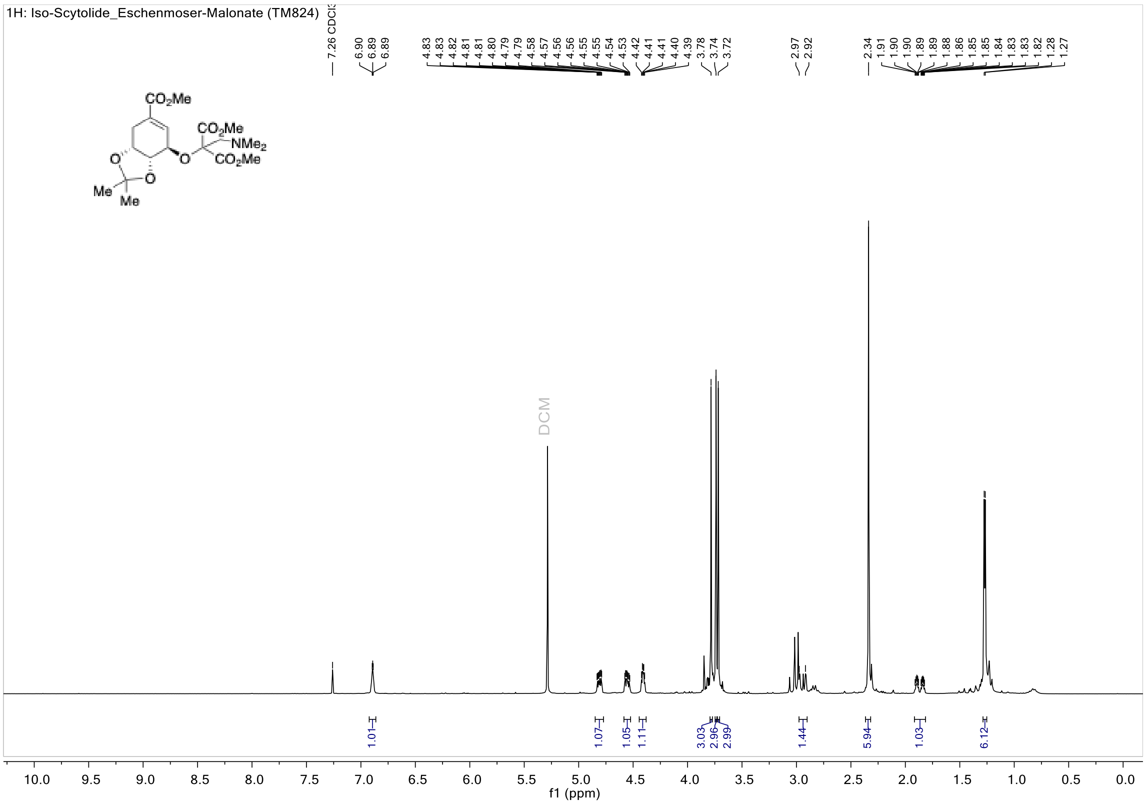


**Fig. S12** $^{1}$H-NMR spectrum of dimethyl 2-((dimethylamino)methyl)-2-(((3a*R*,4*R*,7a*R*)-6-(methoxycarbonyl)-2,2-dimethyl-3a,4,7,7a-tetrahydrobenzo[*d*][1,3]dioxol-4-yl)oxy)malonate measured in $CDCl_3$ at 300 MHz.

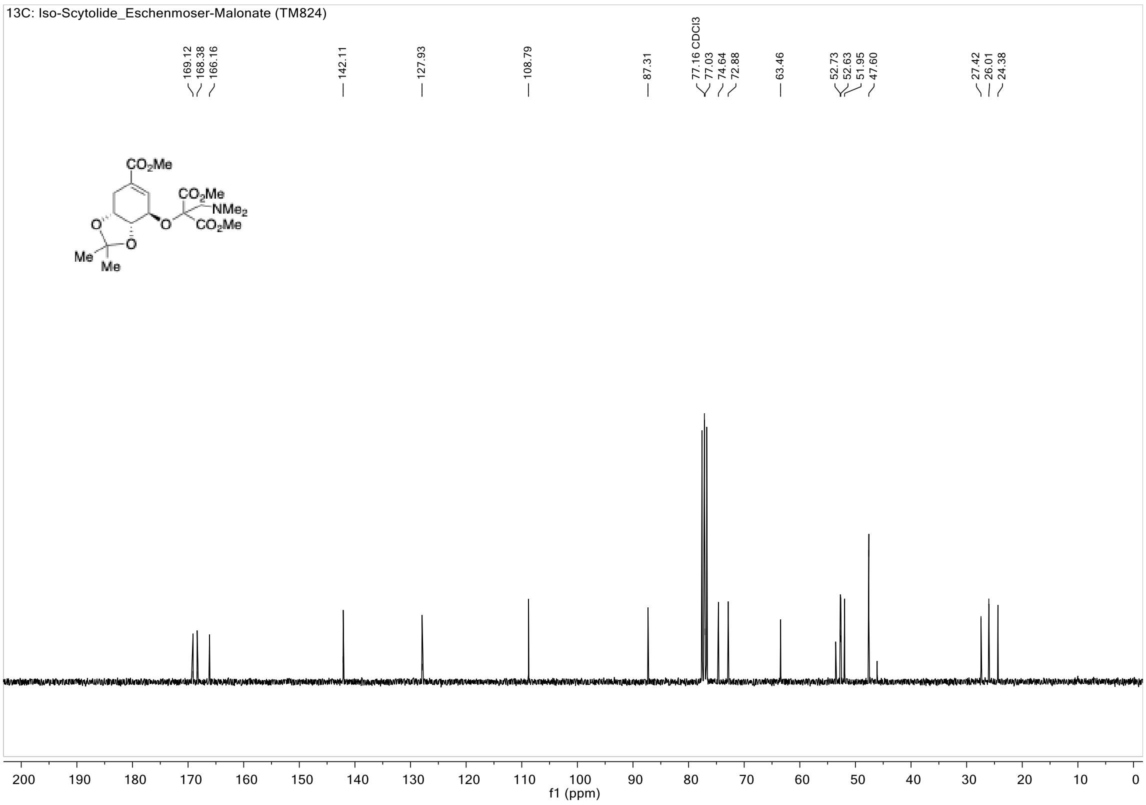


**Fig. S13** $^{13}$C-NMR spectrum of dimethyl 2-((dimethylamino)methyl)-2-(((3a*R*,4*R*,7a*R*)-6-(methoxycarbonyl)-2,2-dimethyl-3a,4,7,7a-tetrahydrobenzo[*d*][1,3]dioxol-4-yl)oxy)malonate measured in $CDCl_3$ at 75 MHz.

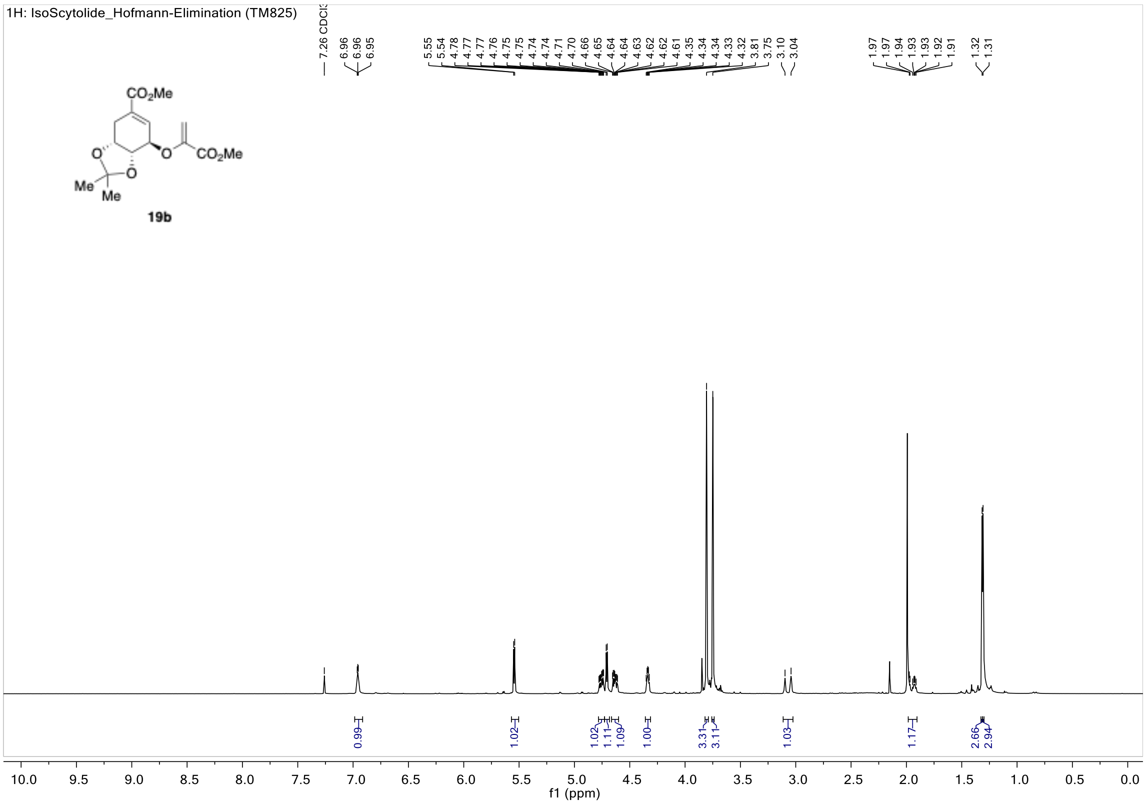


**Fig. S14** $^{1}$H-NMR spectrum of methyl (3a*R*,7*R*,7a*R*)-7-((3-methoxy-3-oxoprop-1-en-2-yl)oxy)-2,2-dimethyl-3a,4,7,7a-tetra-hydrobenzo[*d*][1,3]dioxole-5-carboxylate (**19b**) measured in $CDCl_3$ at 300 MHz.

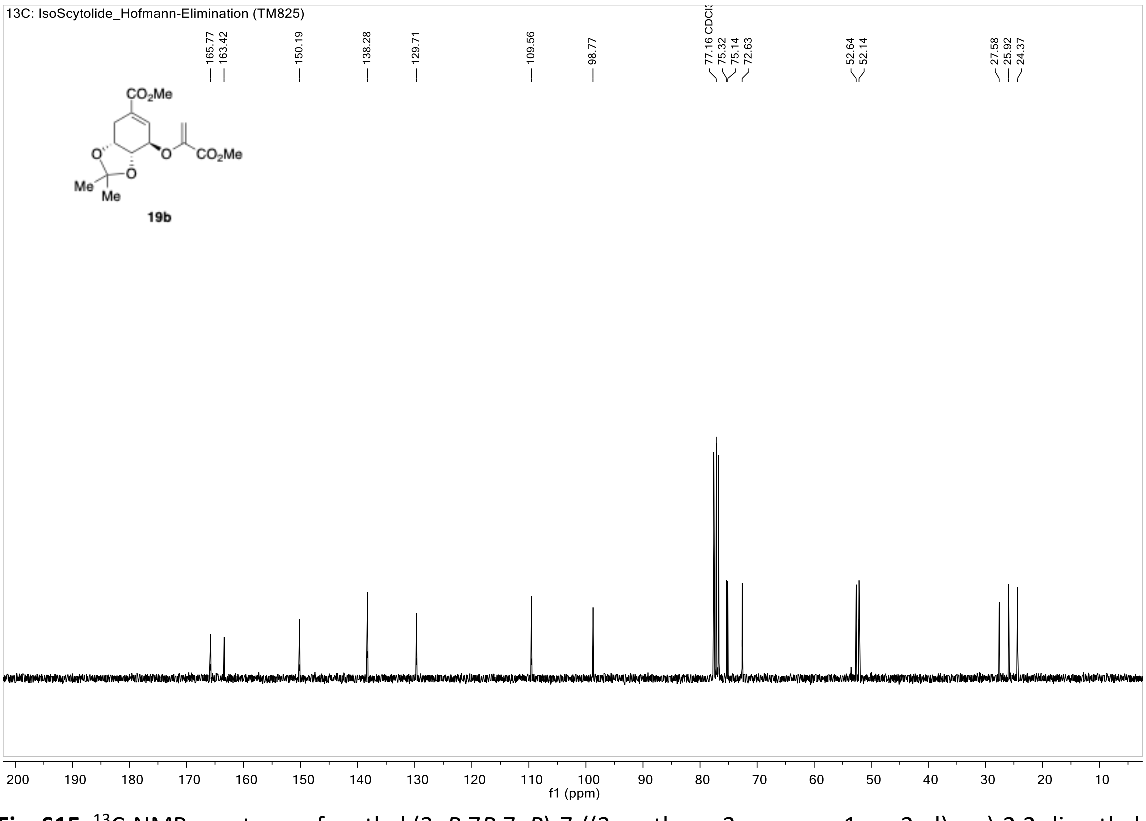


**Fig. S15** $^{13}$C-NMR spectrum of methyl (3a*R*,7*R*,7a*R*)-7-((3-methoxy-3-oxoprop-1-en-2-yl)oxy)-2,2-dimethyl-3a,4,7,7a-tetra-hydrobenzo[*d*][1,3]dioxole-5-carboxylate (**19b**) measured in $CDCl_3$ at 75 MHz.

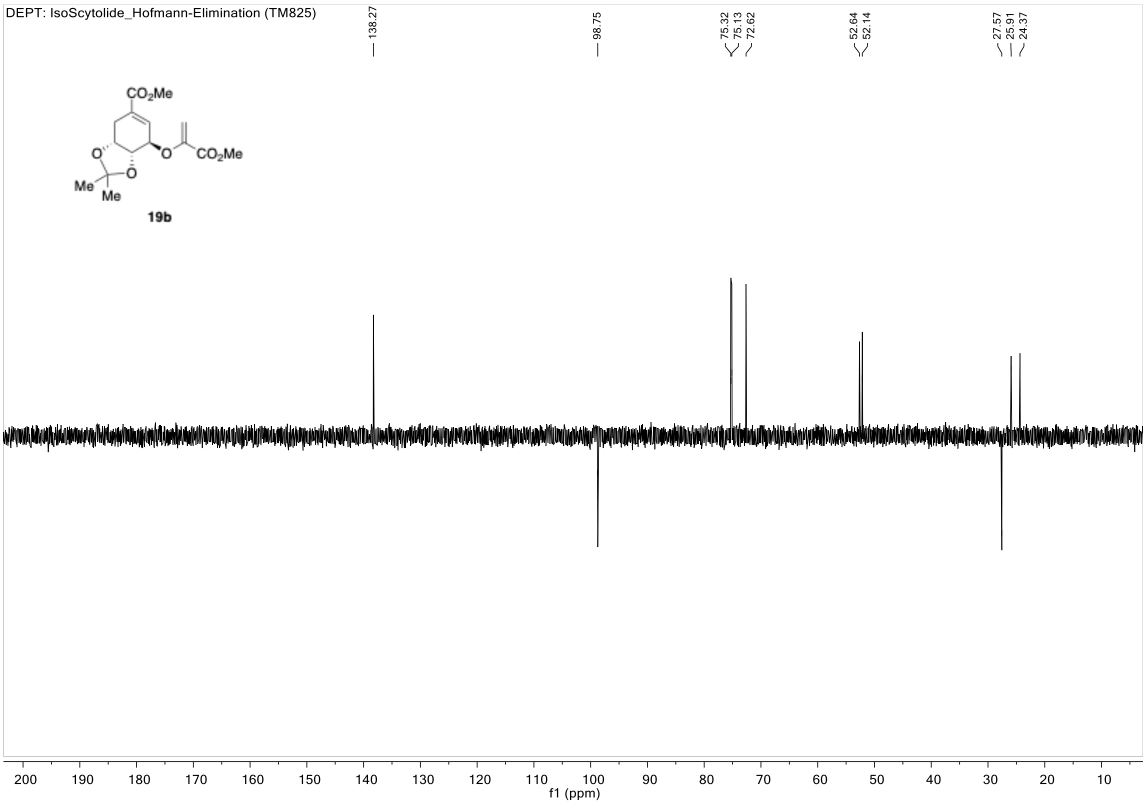


**Fig. S16** DEPT spectrum of methyl (3a*R*,7*R*,7a*R*)-7-((3-methoxy-3-oxoprop-1-en-2-yl)oxy)-2,2-dimethyl-3a,4,7,7a-tetra-hydrobenzo[*d*][1,3]dioxole-5-carboxylate (**19b**) measured in $CDCl_3$ at 75 MHz.

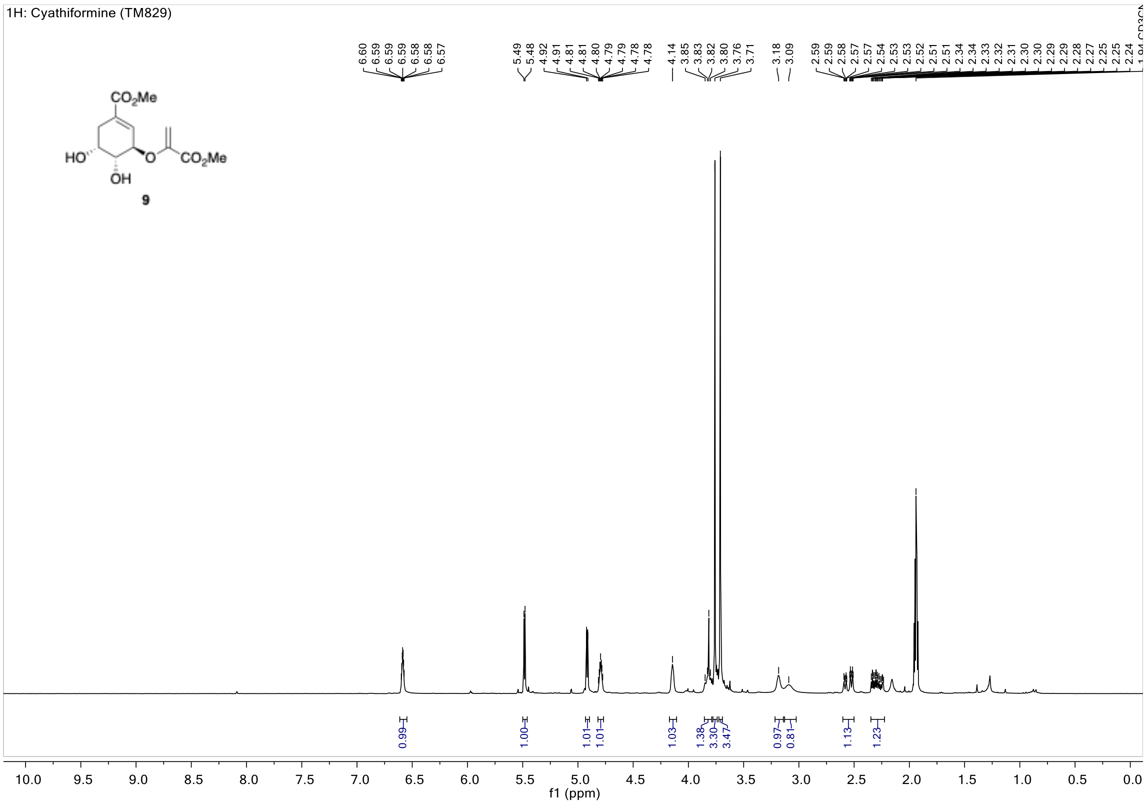


**Fig. S15** $^{1}$H-NMR spectrum of cyathiformine (**9**) measured in $CD_3CN$ at 300 MHz.

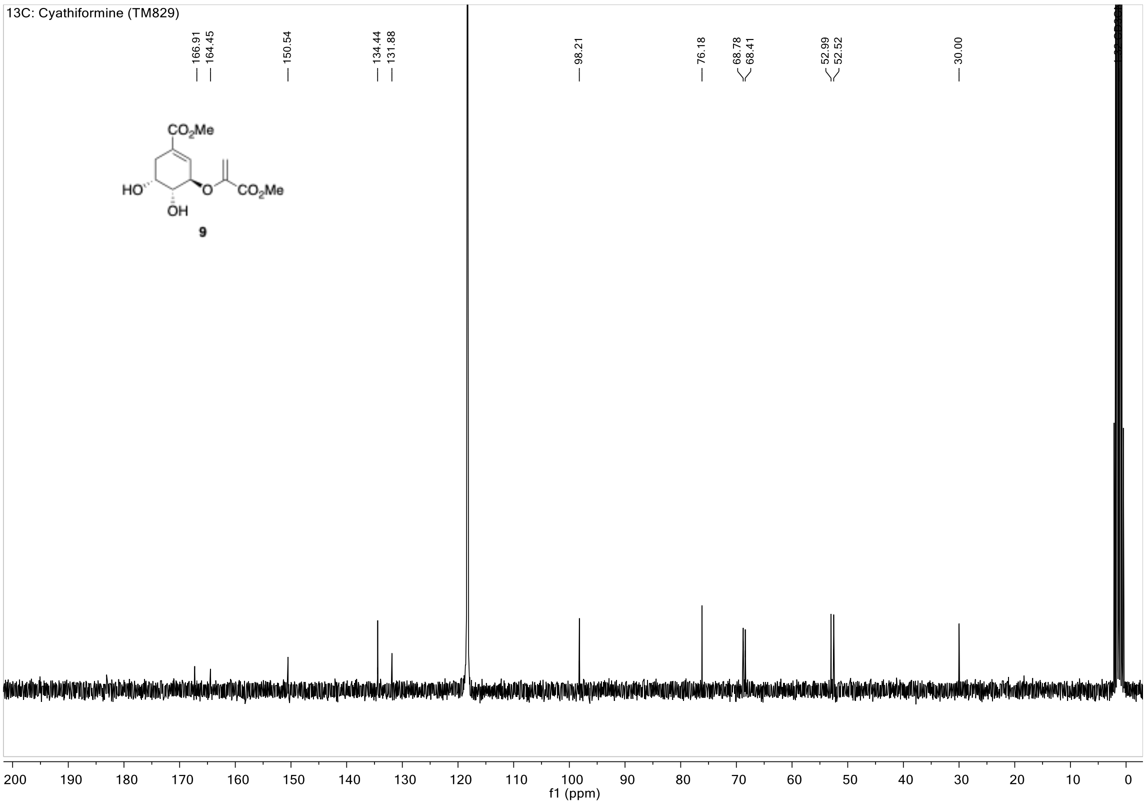


**Fig. S16** $^{13}$C-NMR spectrum of cyathiformine (**9**) measured in $CD_3CN$ at 75 MHz.

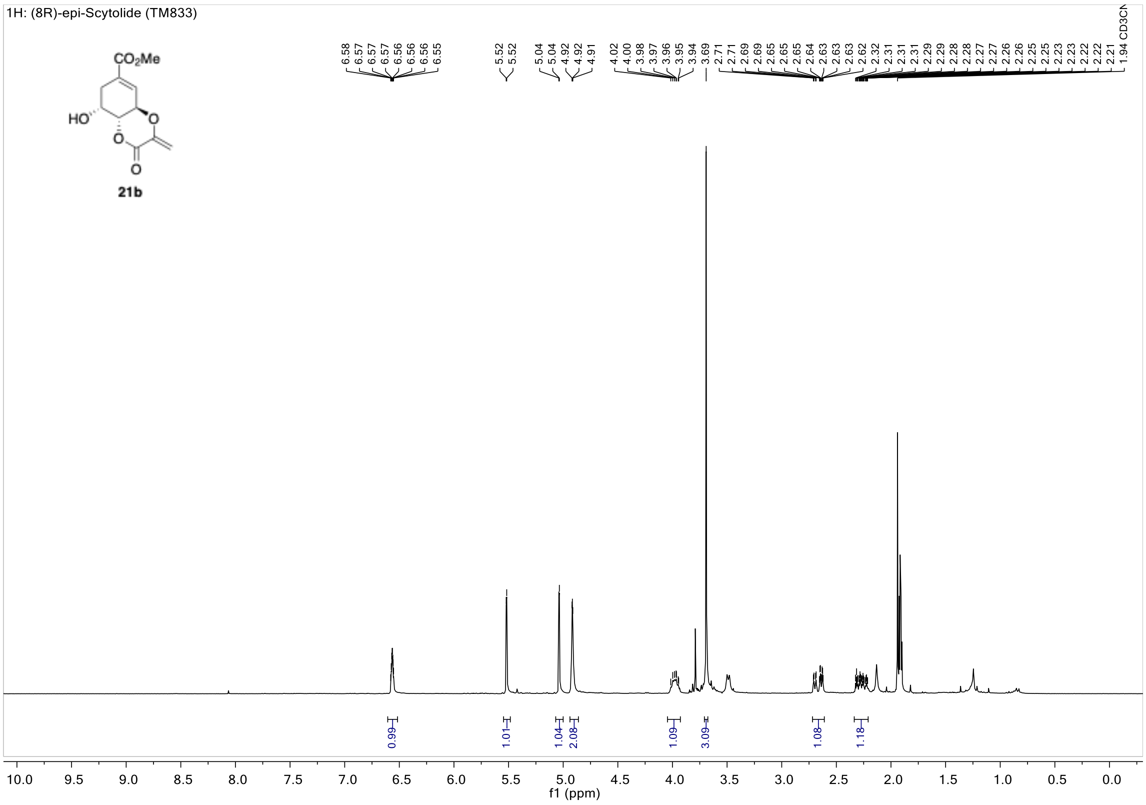


**Fig. S17** $^1$H-NMR spectrum of (8*R*)-*epi*-scytolide (**21b**) measured in $CD_3CN$ at 300 MHz.

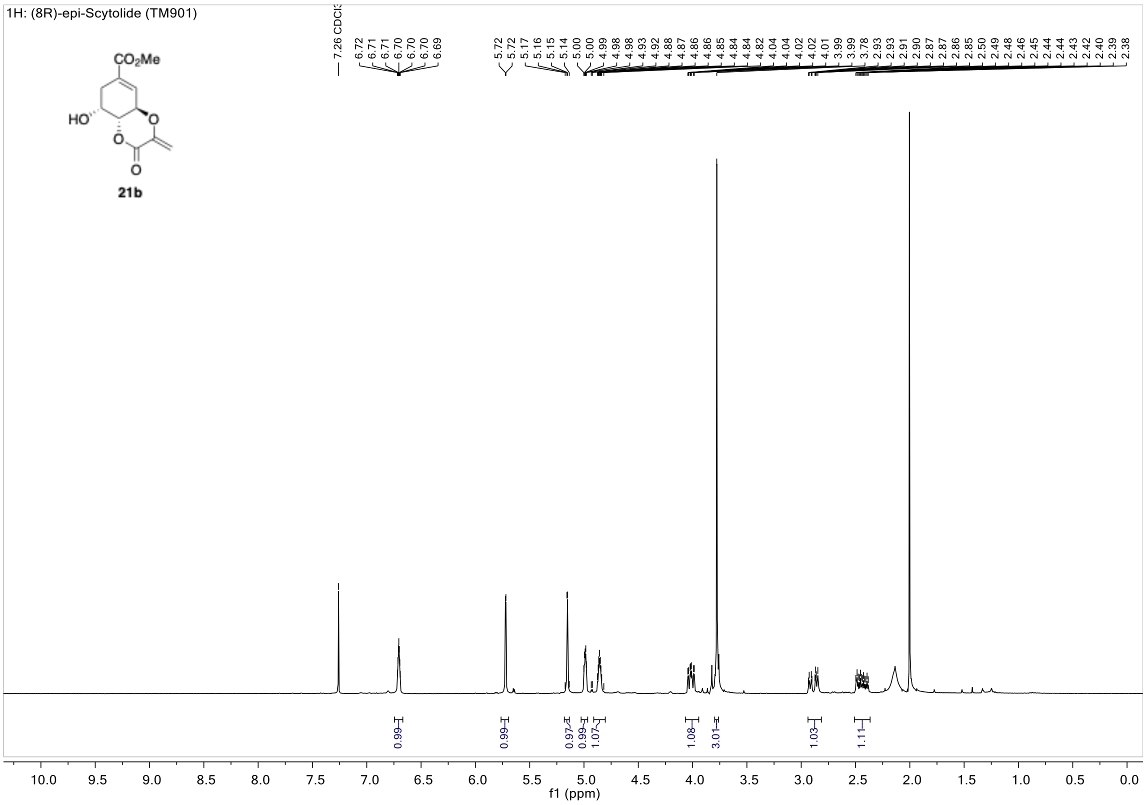


**Fig. S18** $^1$H-NMR spectrum of (8*R*)-*epi*-scytolide (**21b**) measured in $CDCl_3$ at 300 MHz.

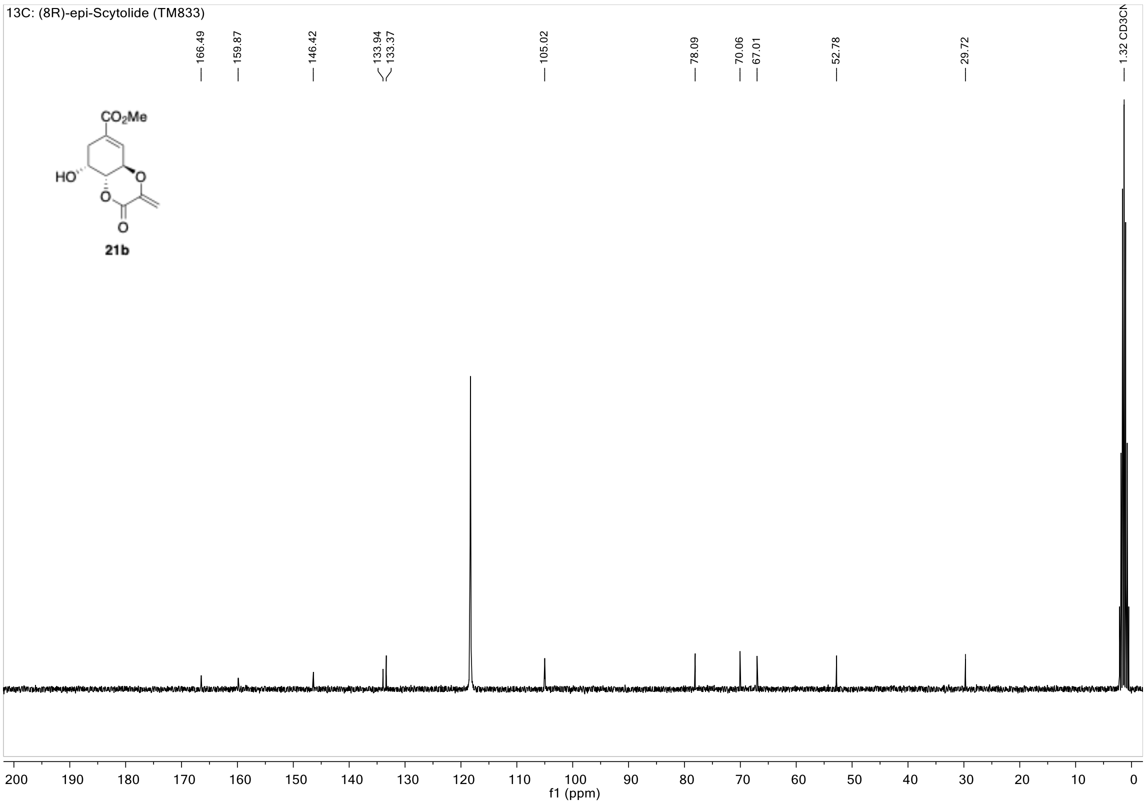


**Fig. S19** $^{13}$C-NMR spectrum of (8*R*)-*epi*-scytolide (**21b**) measured in $CD_3CN$ at 75 MHz.

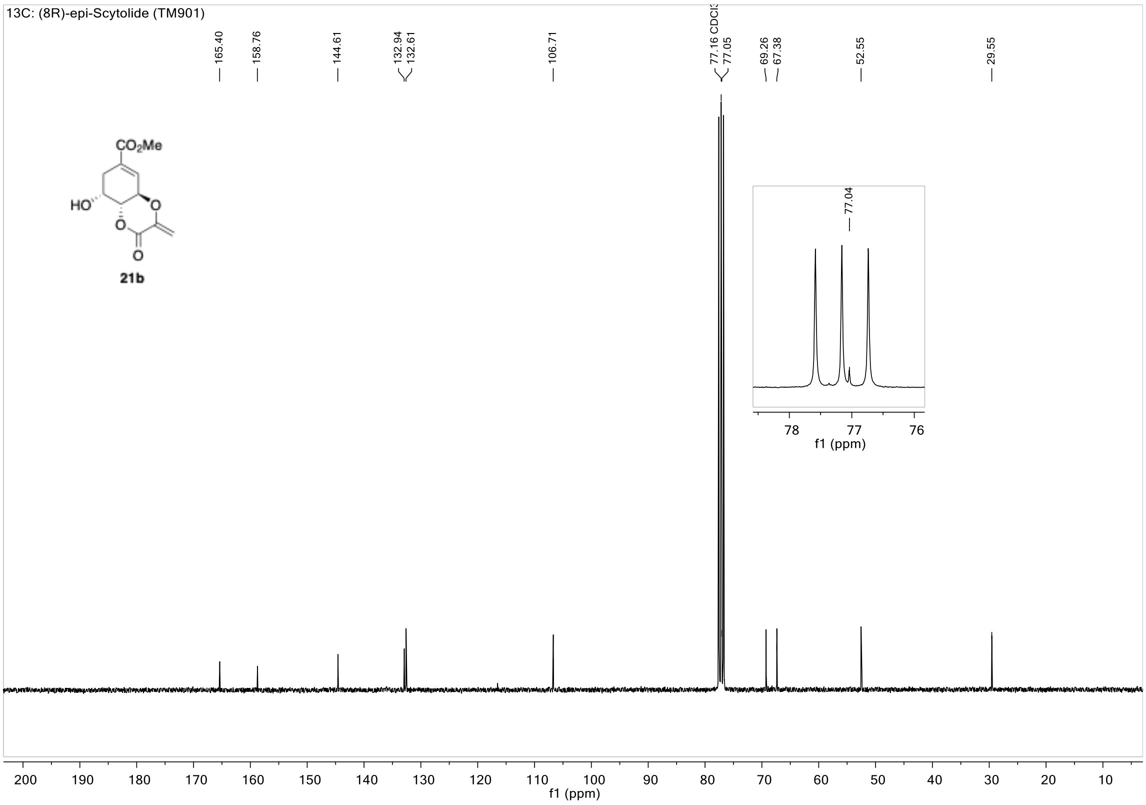


**Fig. S20** $^{13}$C-NMR spectrum of (8*R*)-*epi*-scytolide (**21b**) measured in $CDCl_3$ at 75 MHz.

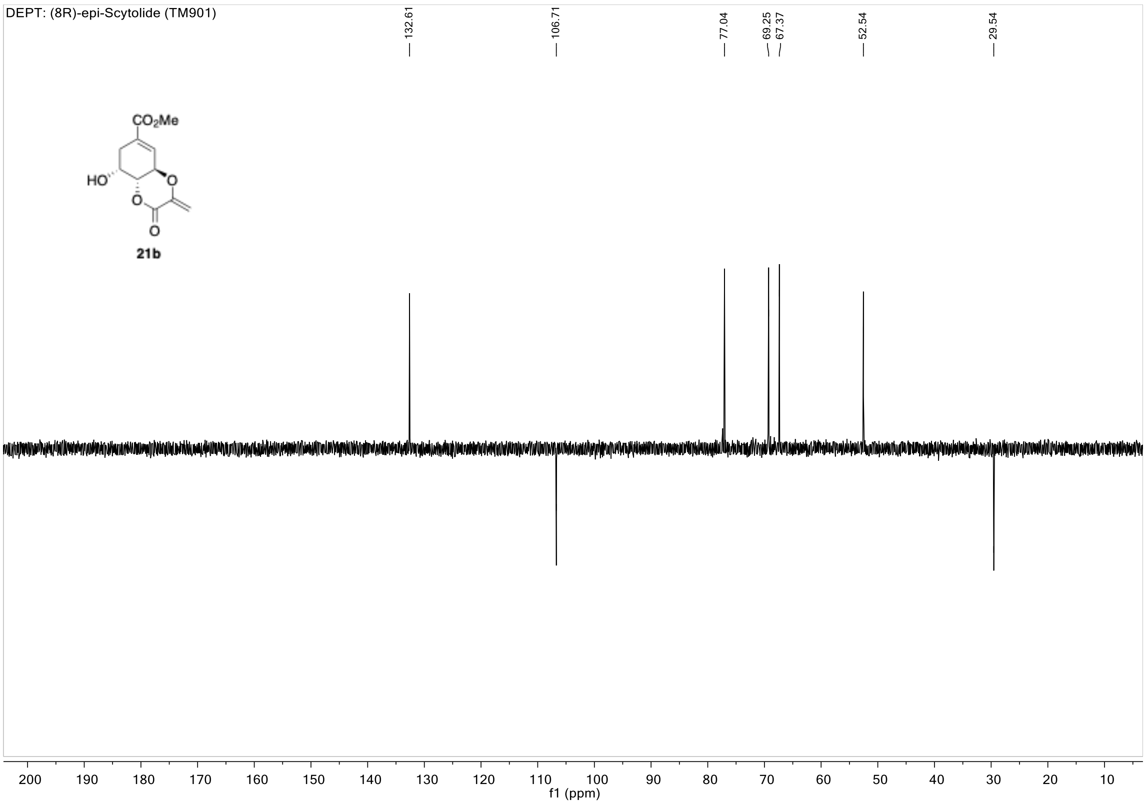


**Fig. S21** DEPT spectrum of (8*R*)-*epi*-scytolide (**21b**) measured in $CDCl_3$ at 75 MHz.

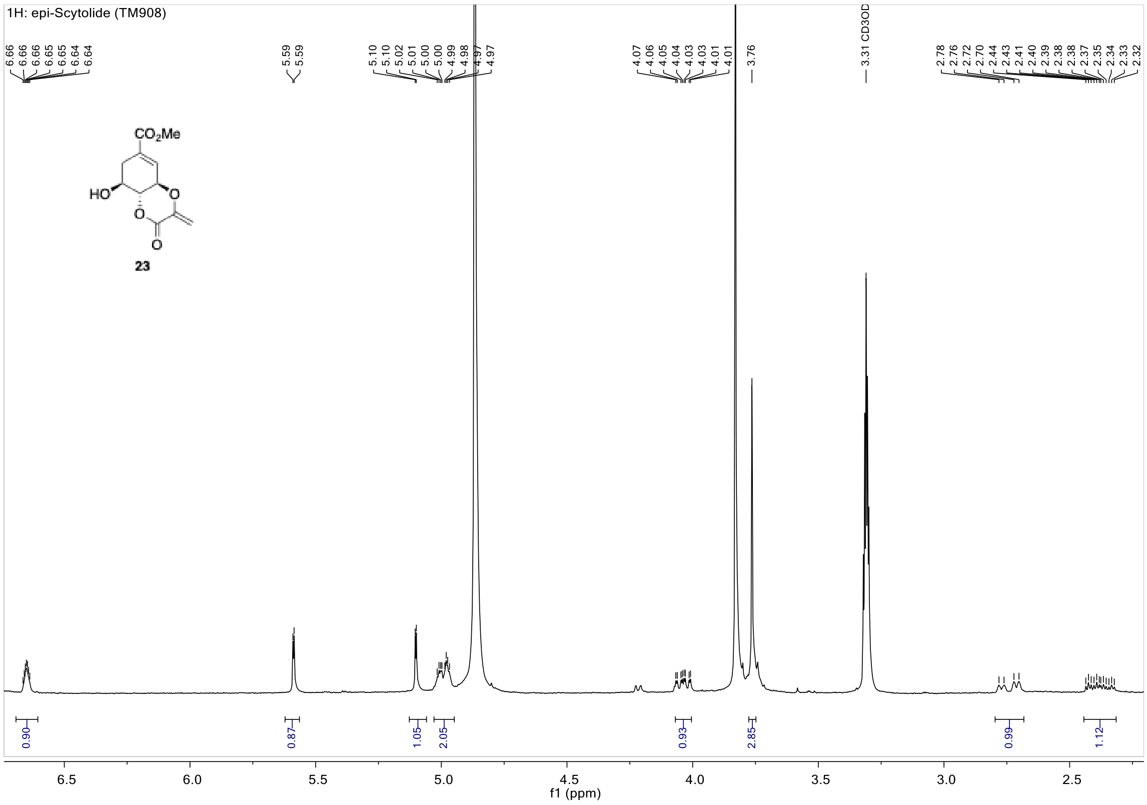


**Fig. S22** $^1$H-NMR spectrum of crude *epi*-scytolide (**23**), measured in MeOD at 300 MHz.

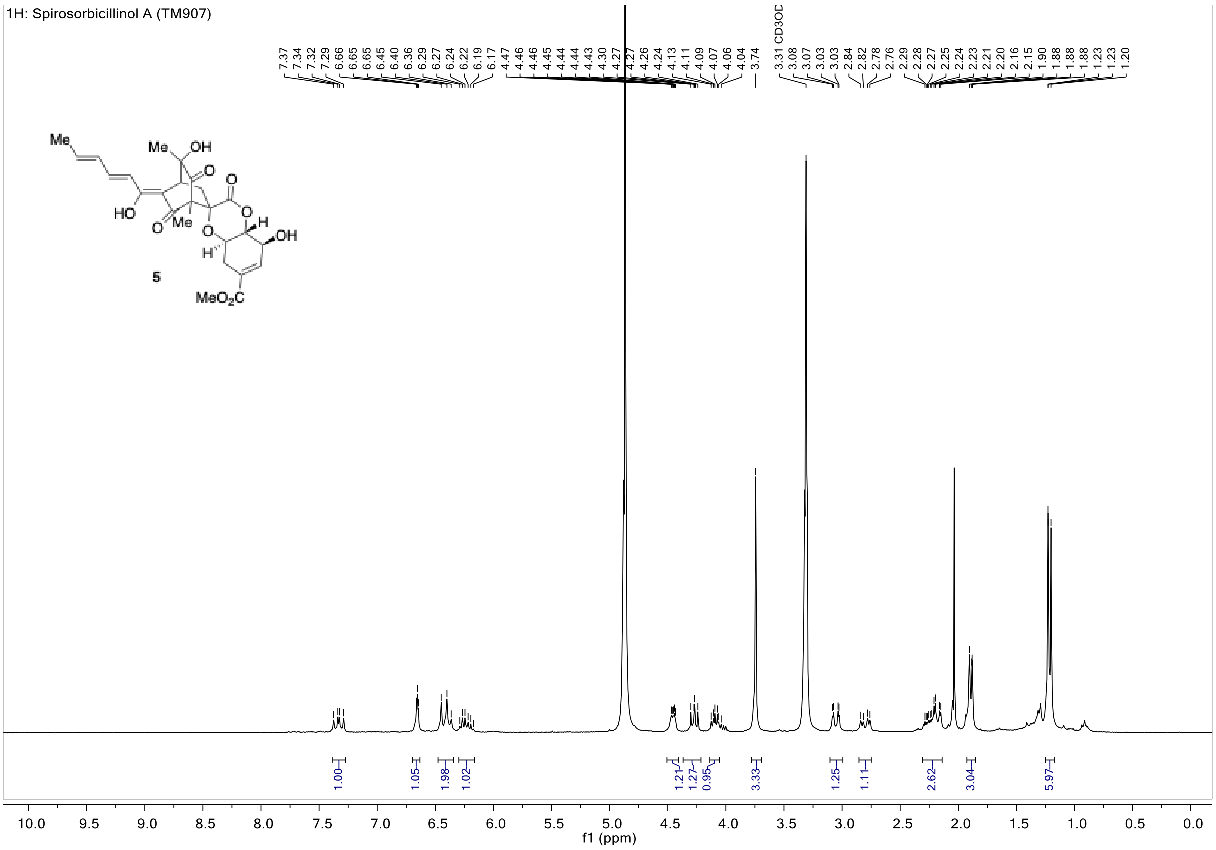


**Fig. S23** $^1$H-NMR spectrum of spirosorbicillinol A (**5**), measured in $CD_3OD$ at 300 MHz.

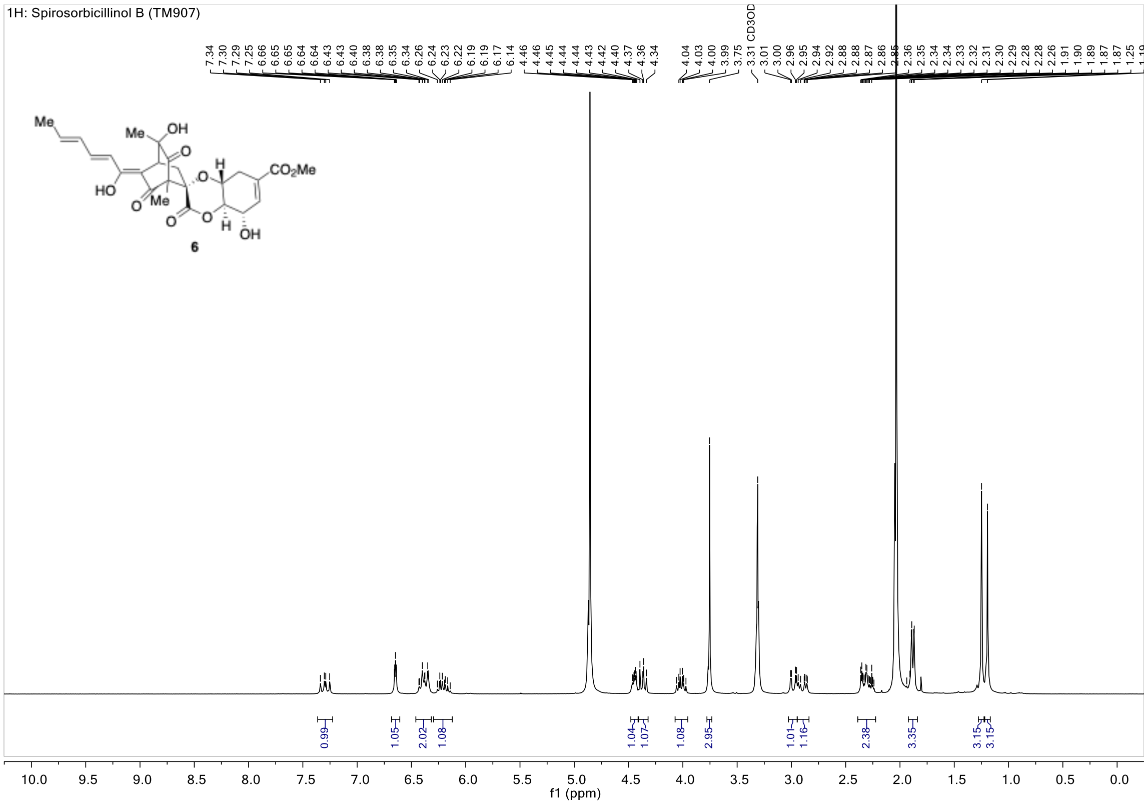


**Fig. S24** $^{1}$H-NMR spectrum of spirosorbicillinol B (**6**), measured in $CD_3OD$ at 300 MHz.

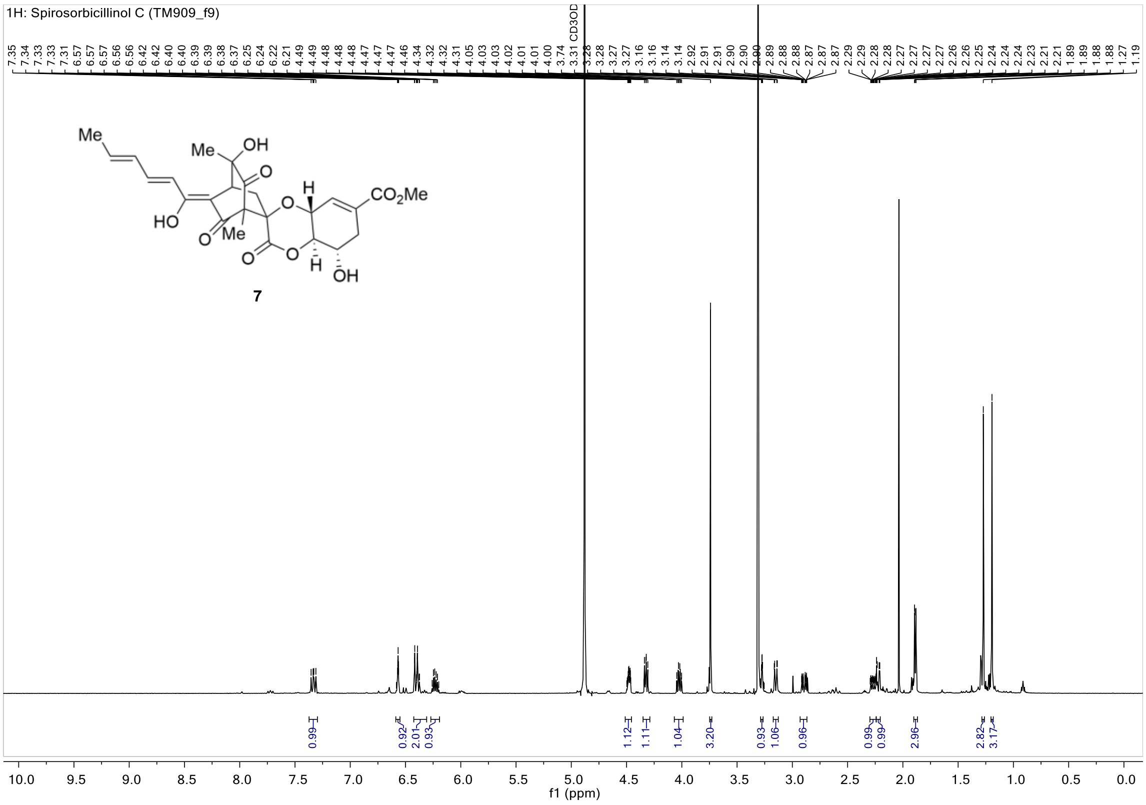


**Fig. S25** $^{1}$H-NMR spectrum of synthesized spirosorbicillinol C (**7**), measured in $CD_3OD$ at 600 MHz.

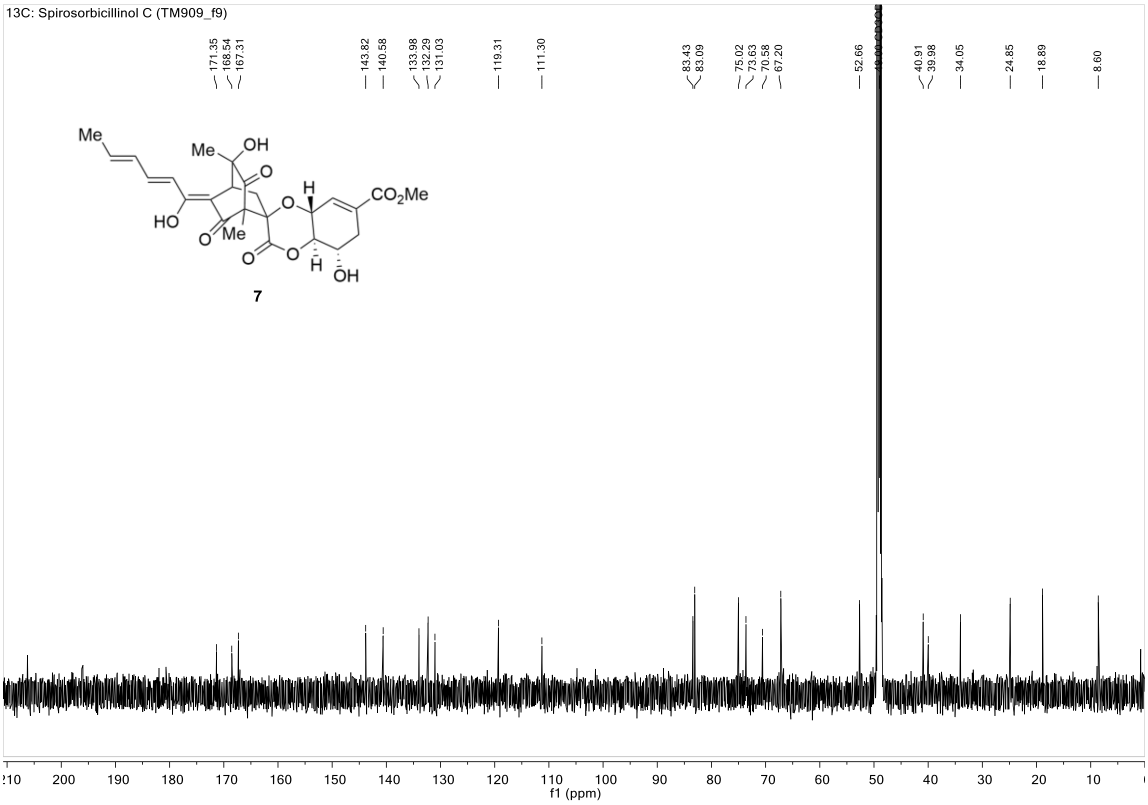


**Fig. S26** $^{13}$C-NMR spectrum of synthesized spirosorbicillinol C (**7**), measured in $CD_3OD$ at 150 MHz..

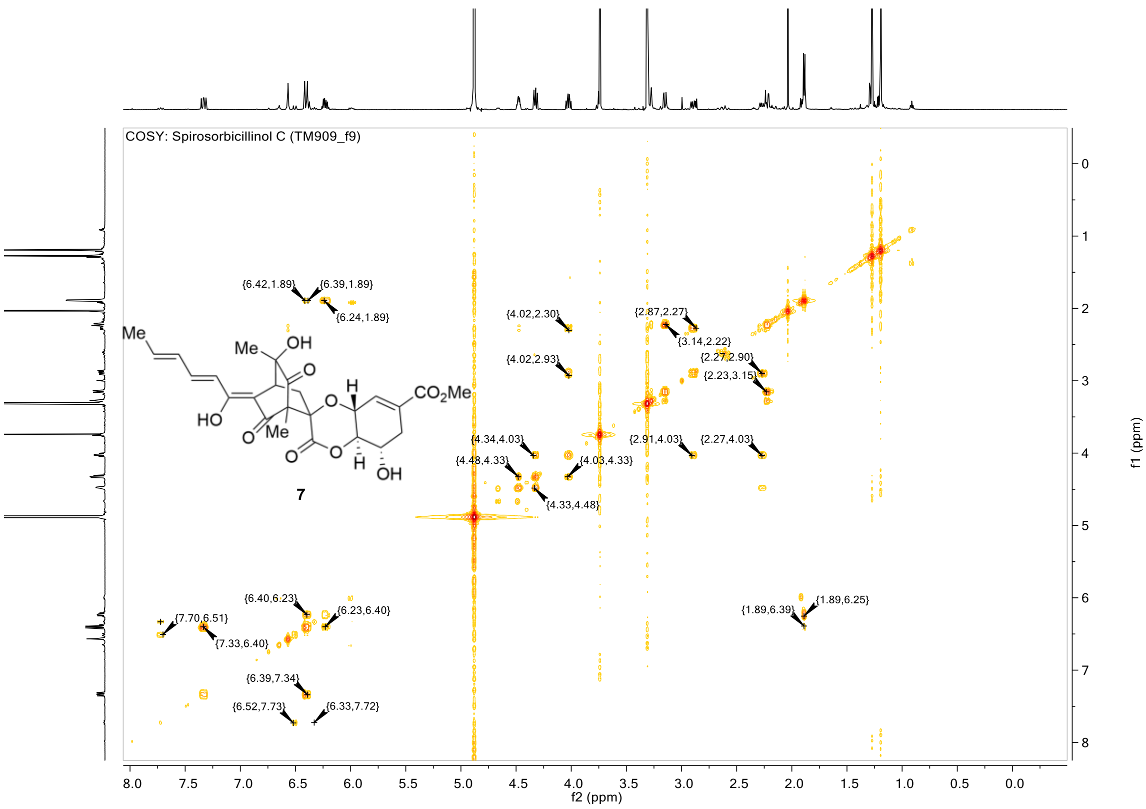


**Fig. S27** COSY spectrum of synthesized spirosorbicillinol C (**7**), measured in $CD_3OD$.

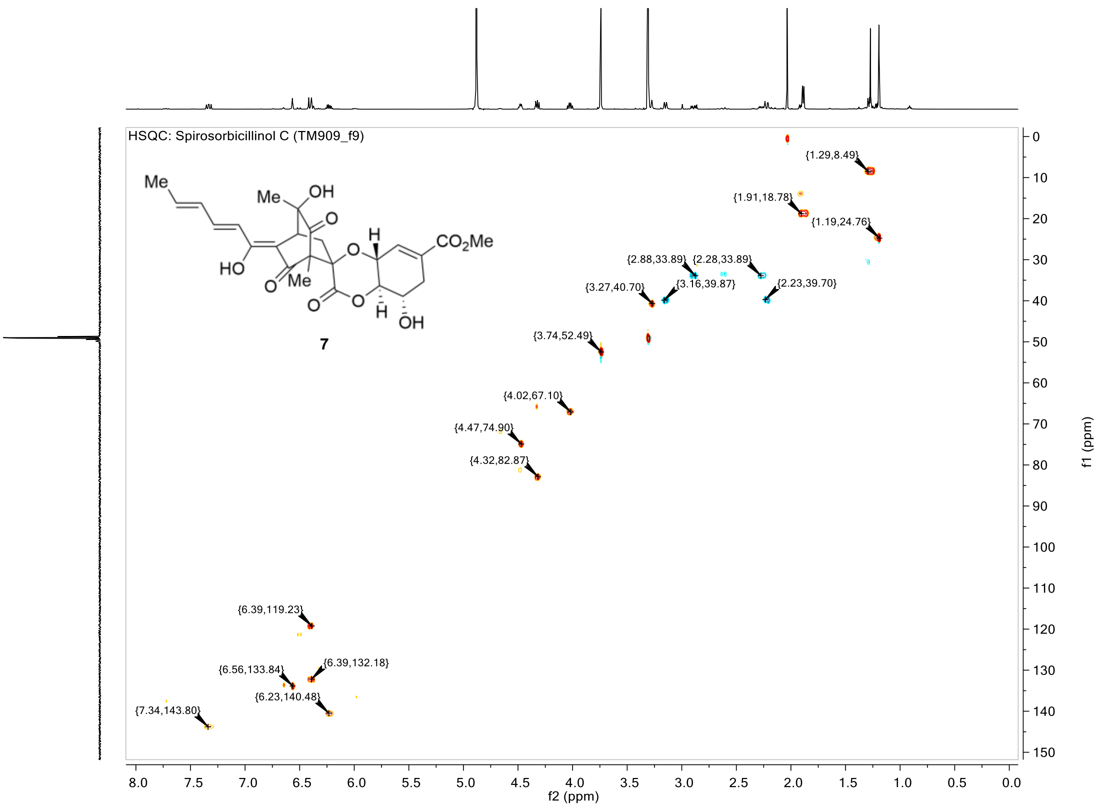


**Fig. S28** HSQC spectrum of synthesized spirosorbicillinol C (**7**), measured in $CD_3OD$.

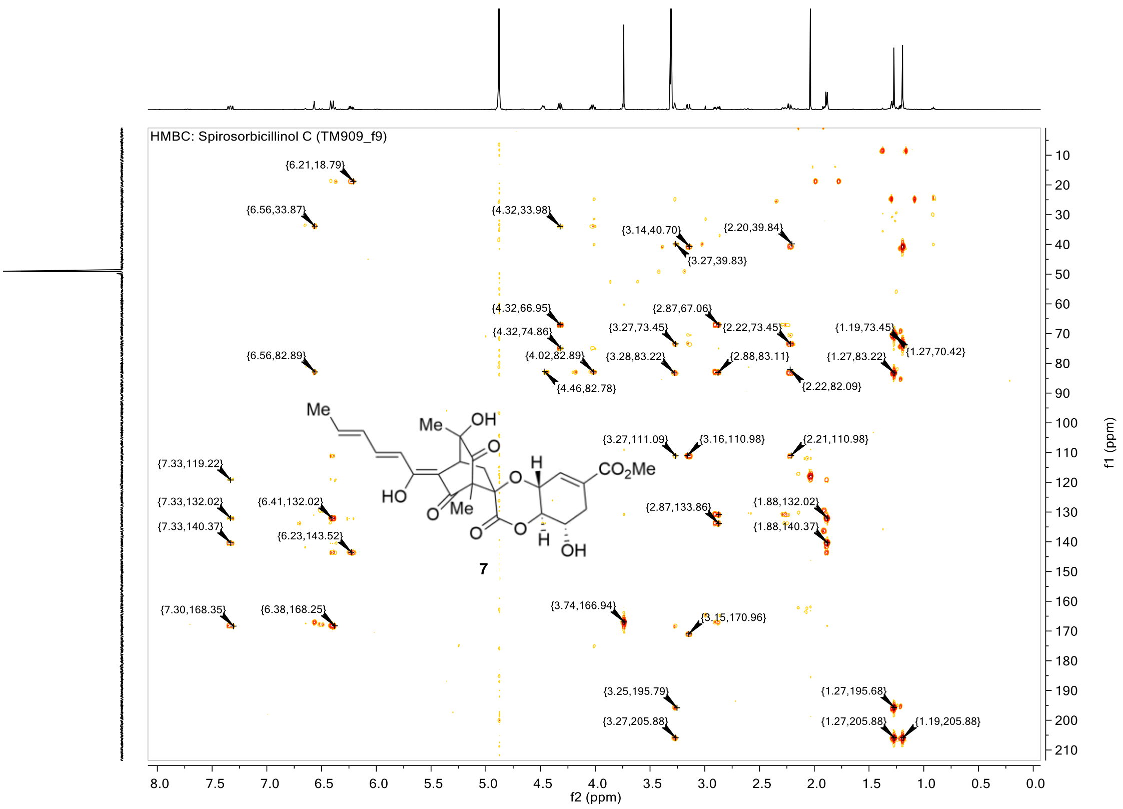


**Fig. S29** HMBC spectrum of synthesized spirosorbicillinol C (**7**), measured in $CD_3OD$.

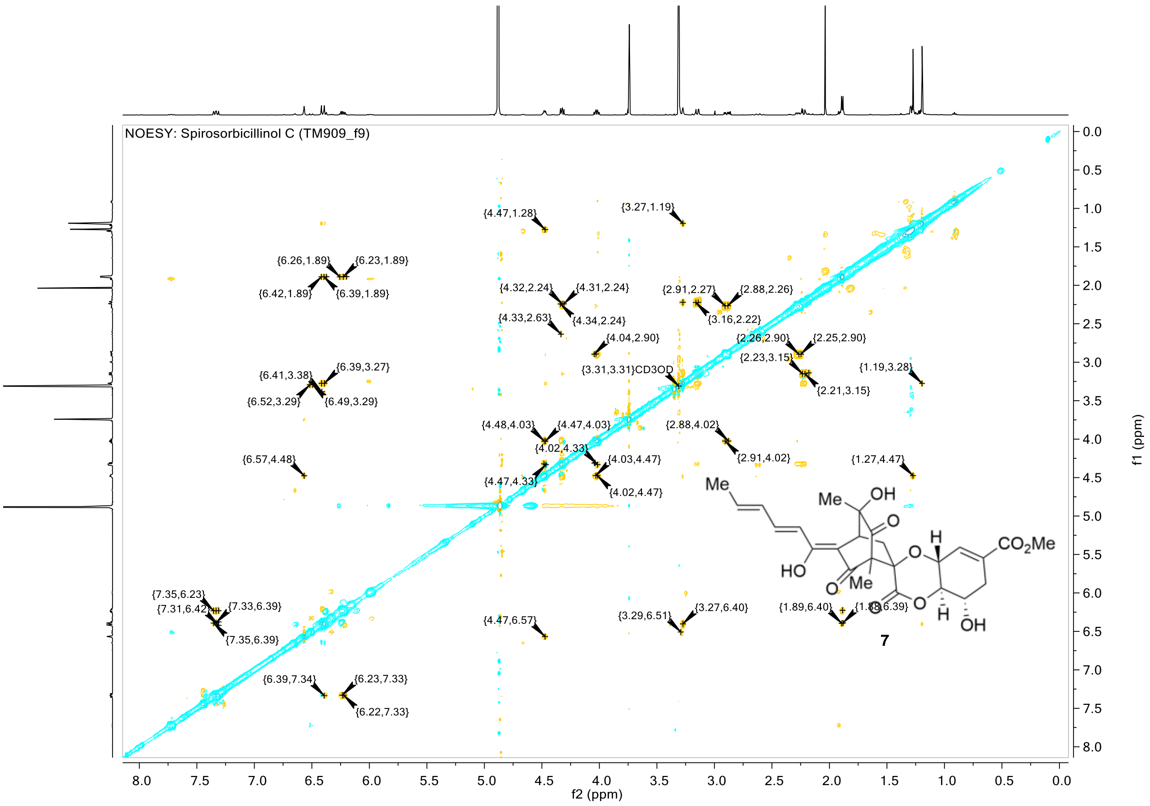


**Fig. S30** NOESY spectrum of synthesized spirosorbicillinol C (**7**), measured in $CD_3OD$.

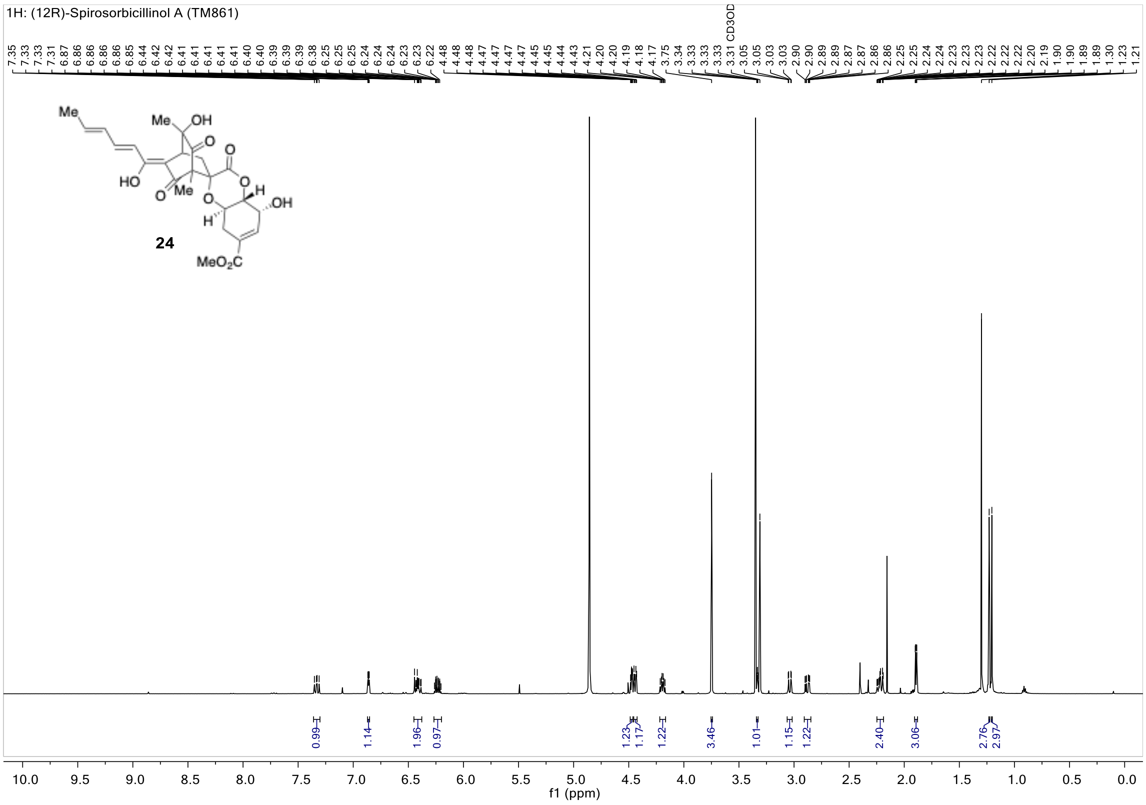

**Fig. S31** $^{1}$H-NMR spectrum of (12*R*)-spirosorbicillinol A (**24**), measured in $CD_3OD$ at 600 MHz.

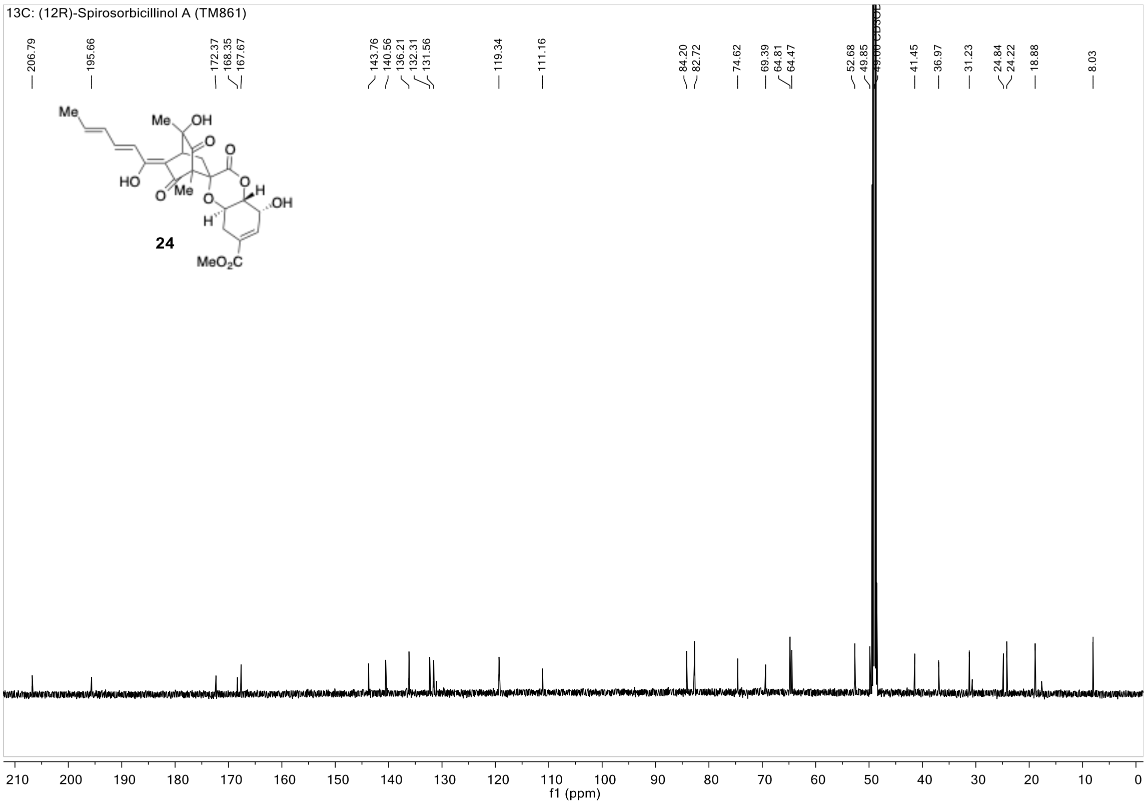

**Fig. S32** $^{13}$C-NMR spectrum of (12*R*)-spirosorbicillinol A (**24**), measured in $CD_3OD$ at 150 MHz.

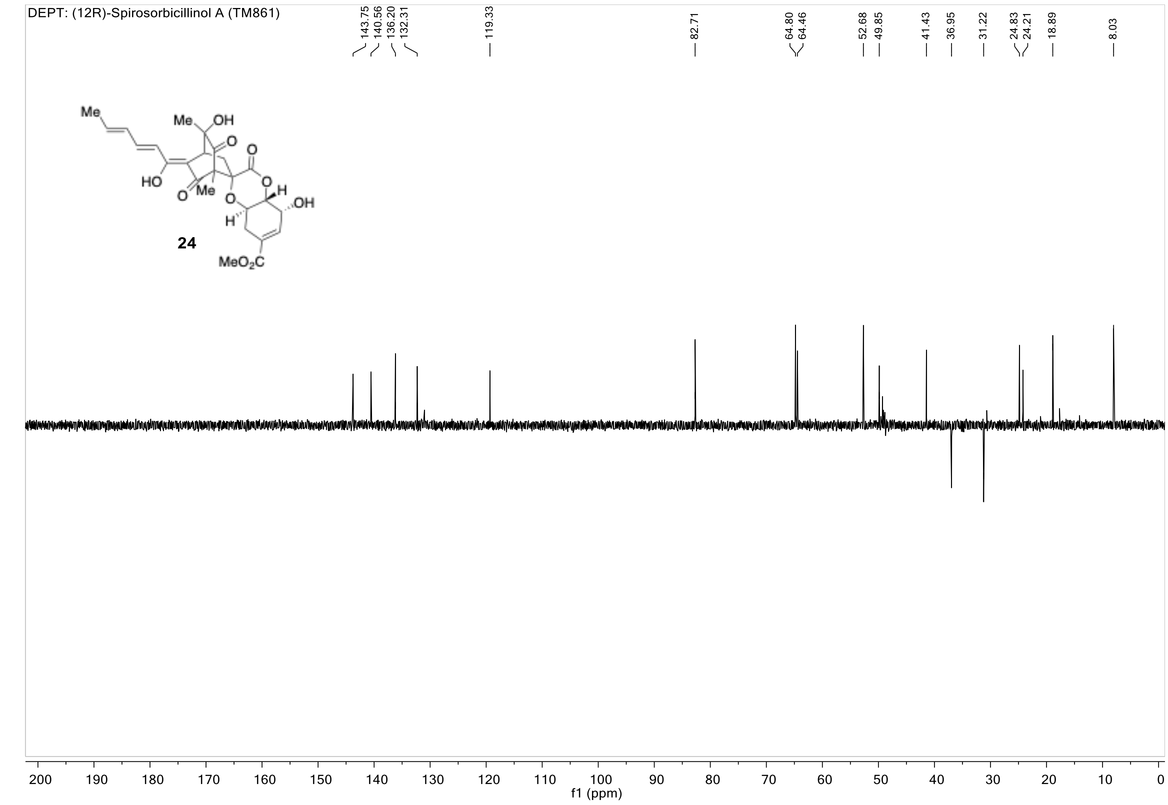

**Fig. S33** DEPT spectrum of (12*R*)-spirosorbicillinol A (**24**), measured in $CD_3OD$ at 150 MHz.

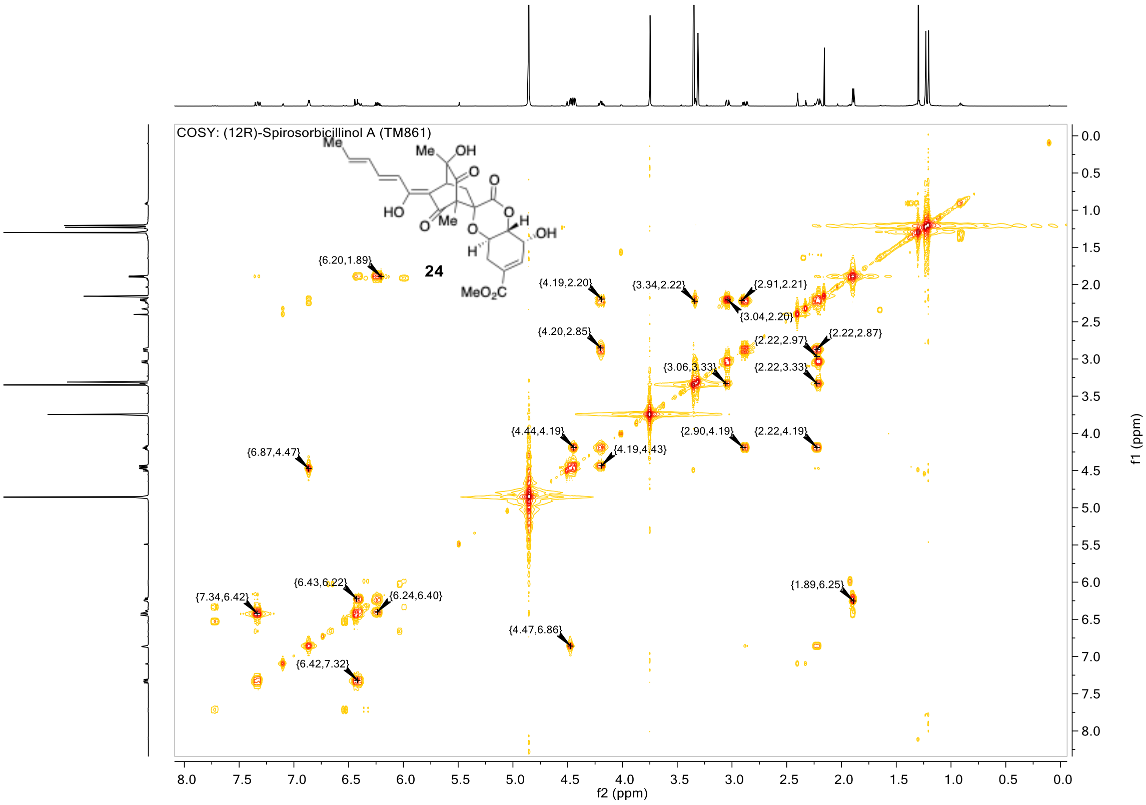

**Fig. S34** COSY spectrum of (12*R*)-spirosorbicillinol A (**24**), measured in $CD_3OD$.

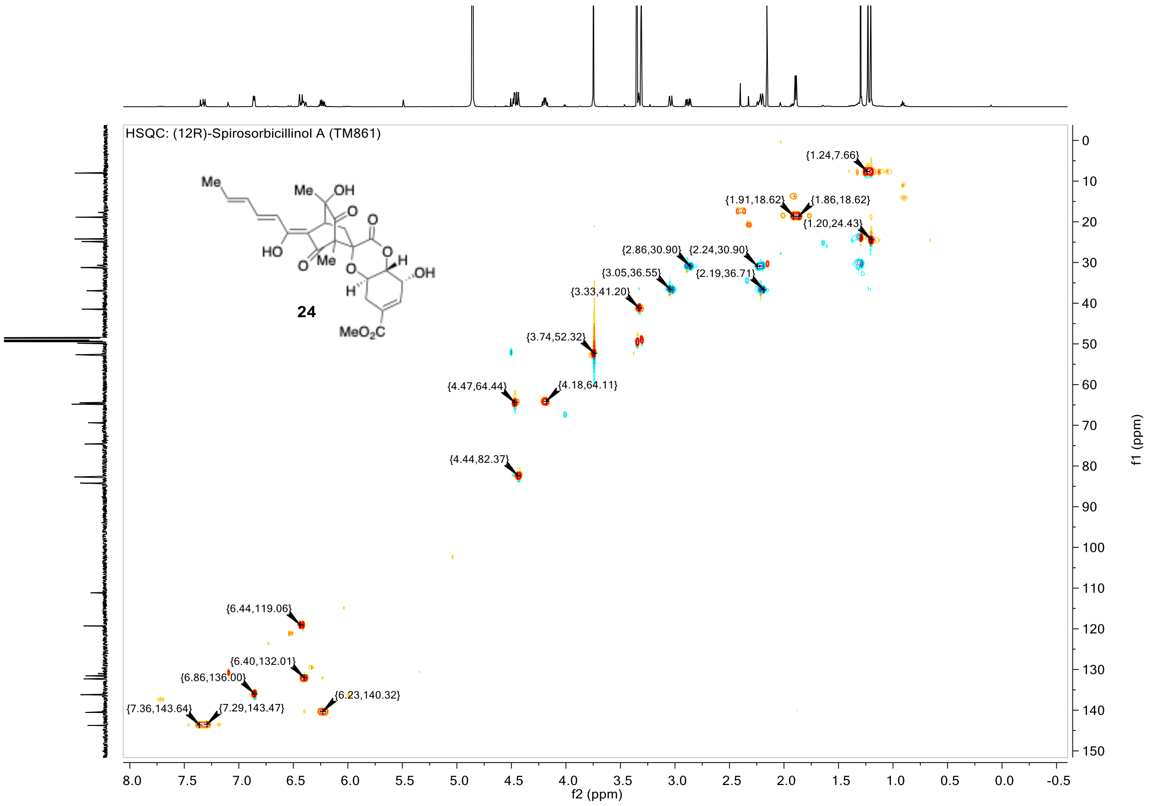

**Fig. S35** HSQC spectrum of (12*R*)-spirosorbicillinol A (**24**), measured in $CD_3OD$.

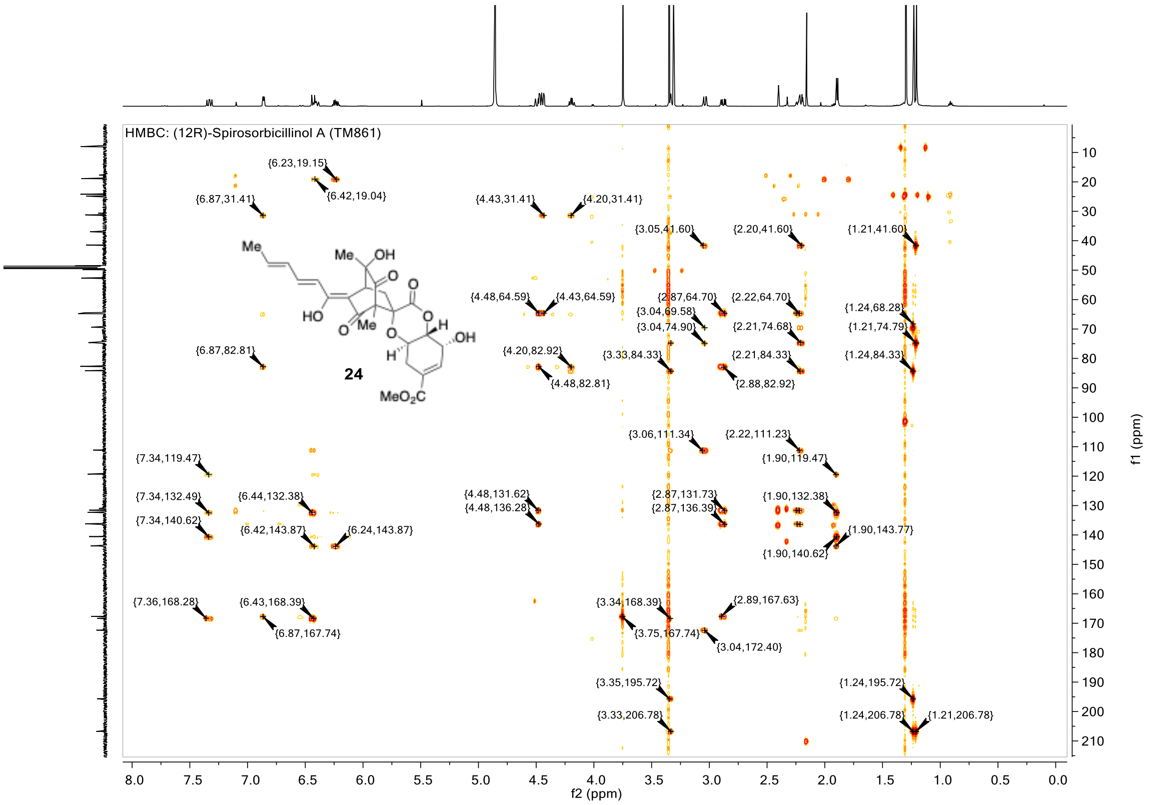

**Fig. S36** HMBC spectrum of (12*R*)-spirosorbicillinol A (**24**), measured in $CD_3OD$.

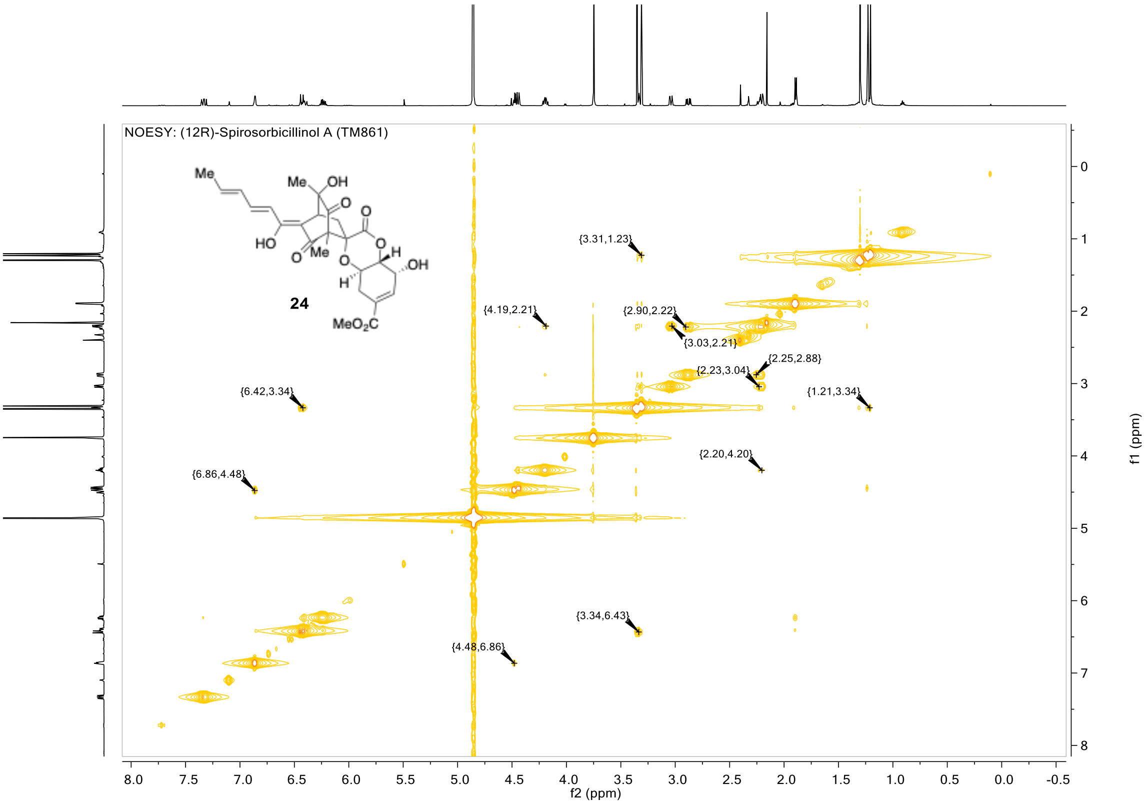


**Fig. S37** NOESY spectrum of (12*R*)-spirosorbicillinol A (**24**), measured in $CD_3OD$.

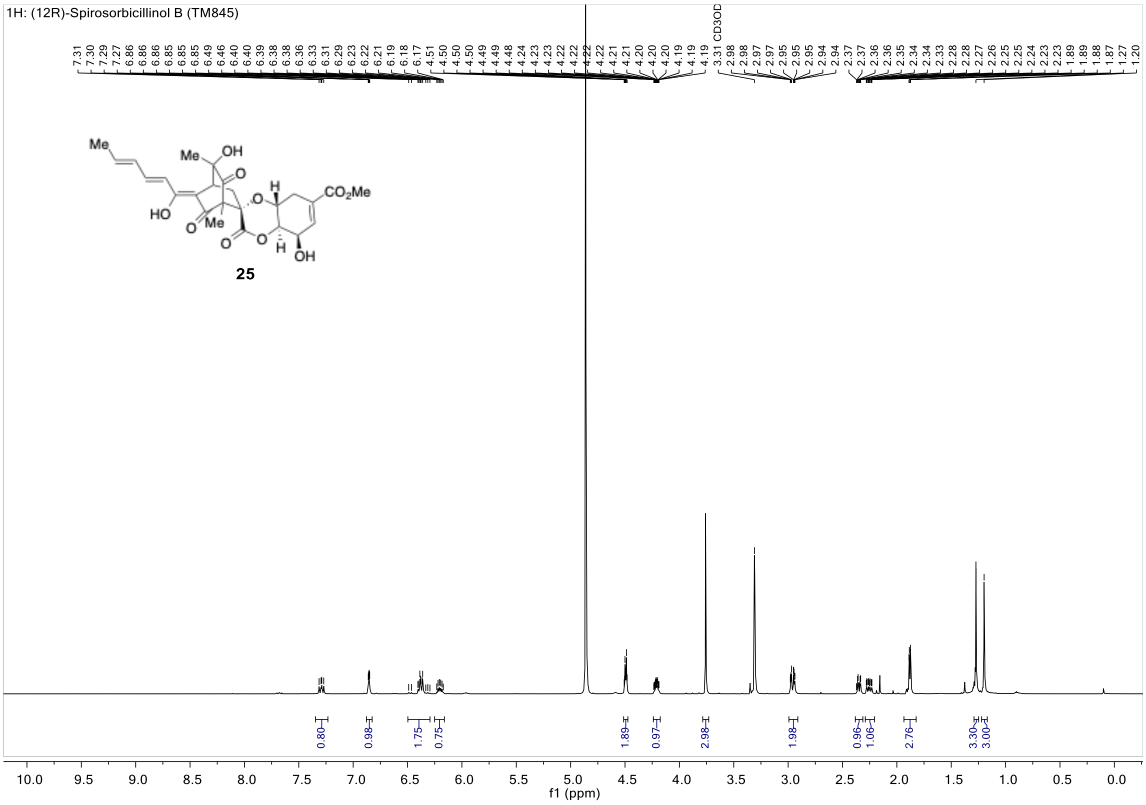


**Fig. S38** ¹H-NMR spectrum of (12*R*)-spirosorbicillinol B (**25**), measured in $CD_3OD$ at 600 MHz.

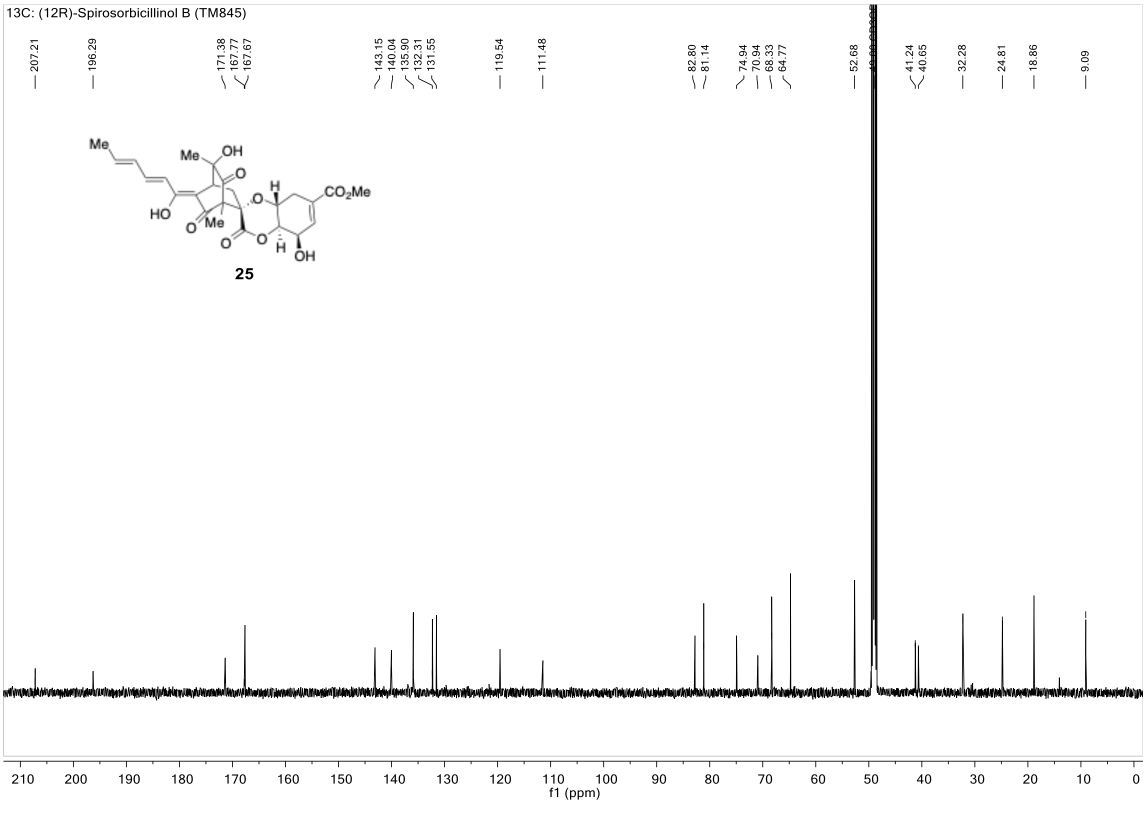


**Fig. S39** ¹³C-NMR spectrum of (12*R*)-spirosorbicillinol B (**25**), measured in $CD_3OD$ at 150 MHz.

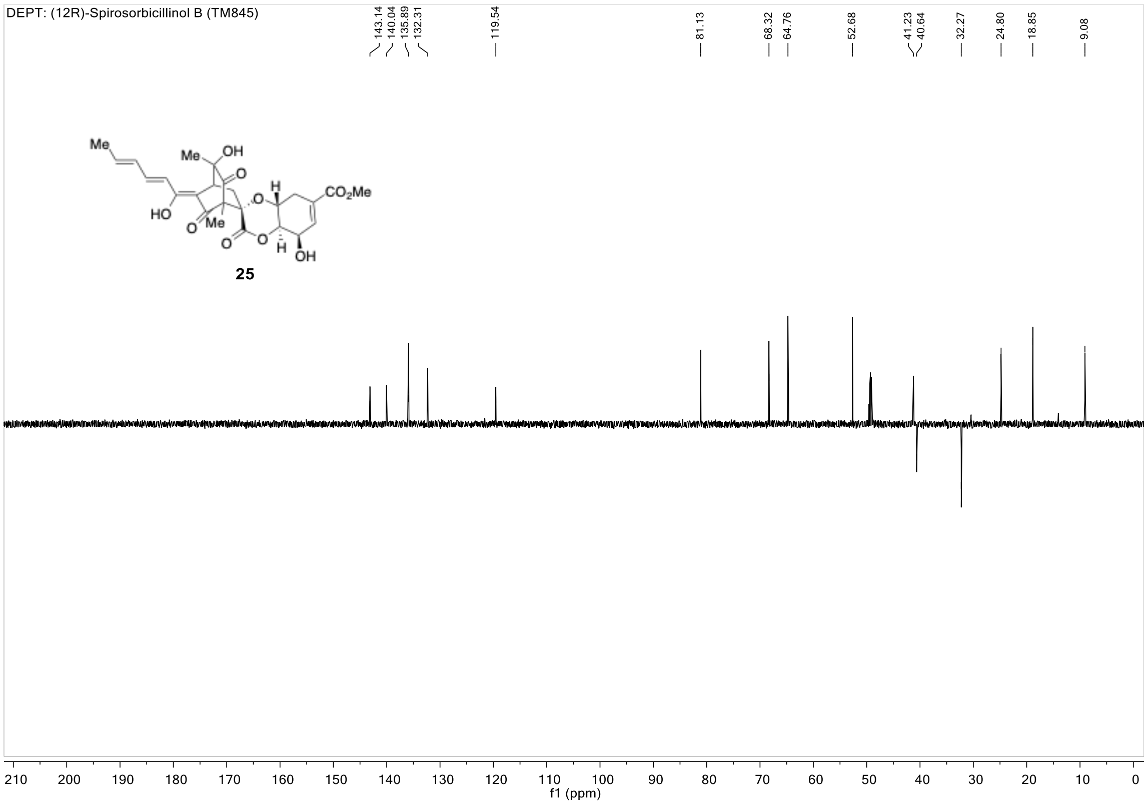


**Fig. S40** DEPT spectrum of (12*R*)-spirosorbicillinol B (**25**), measured in CD3OD at 150 MHz.

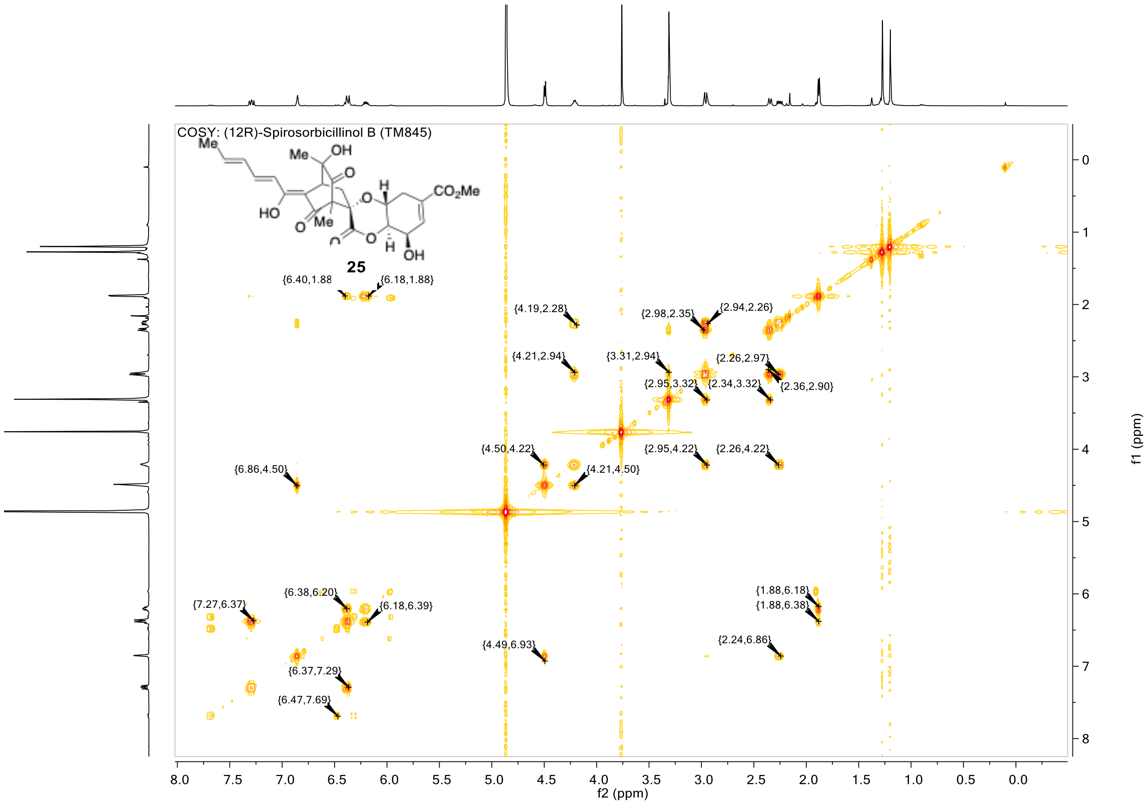


**Fig. S41** COSY spectrum of (12*R*)-spirosorbicillinol B (**25**), measured in CD3OD.

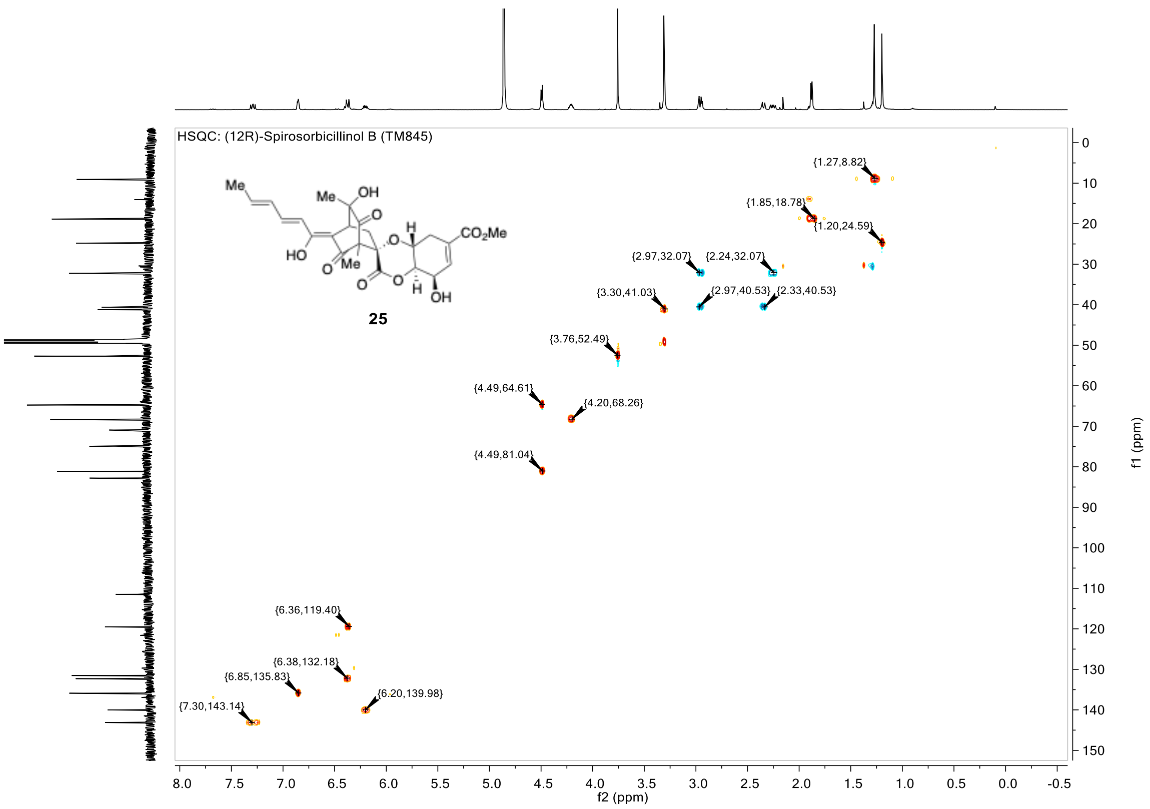


**Fig. S42** HSQC spectrum of (12*R*)-spirosorbicillinol B (**25**), measured in $CD_3OD$.

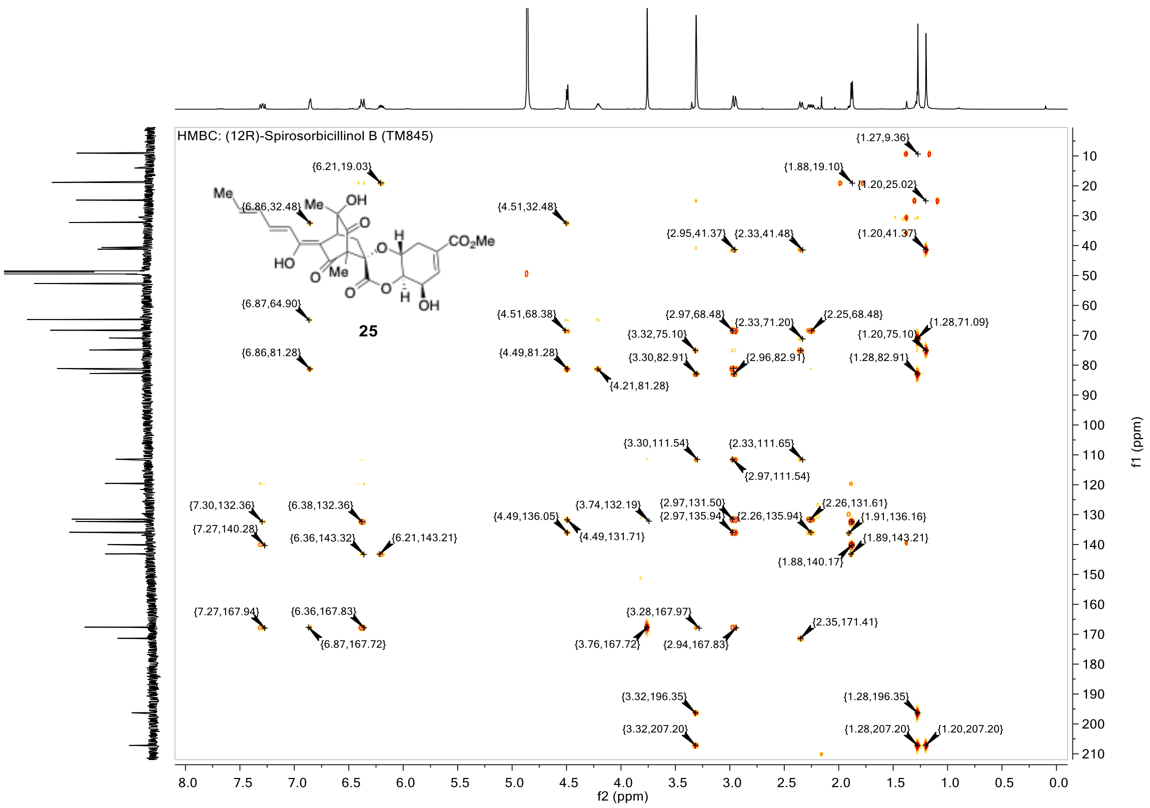


**Fig. S43** HMBC spectrum of (12*R*)-spirosorbicillinol B (**25**), measured in $CD_3OD$.

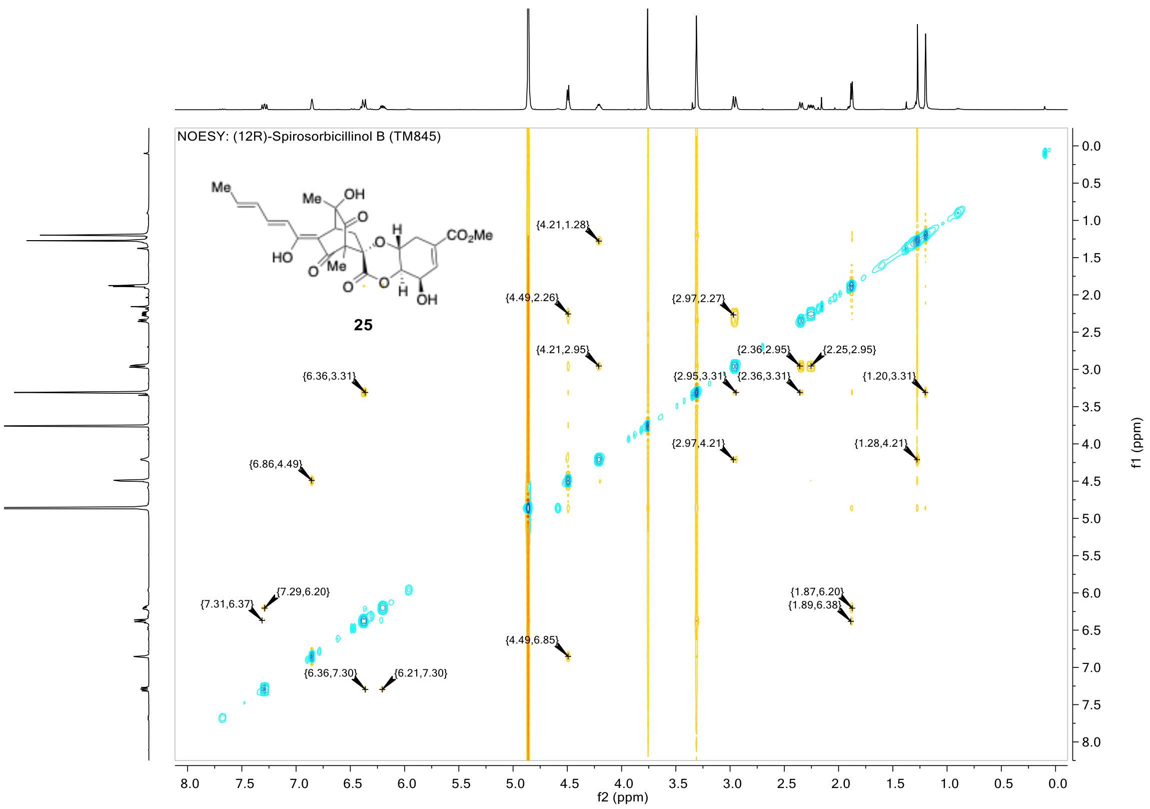


**Fig. S44** NOESY spectrum of (12*R*)-spirosorbicillinol B (**25**), measured in $CD_3OD$.

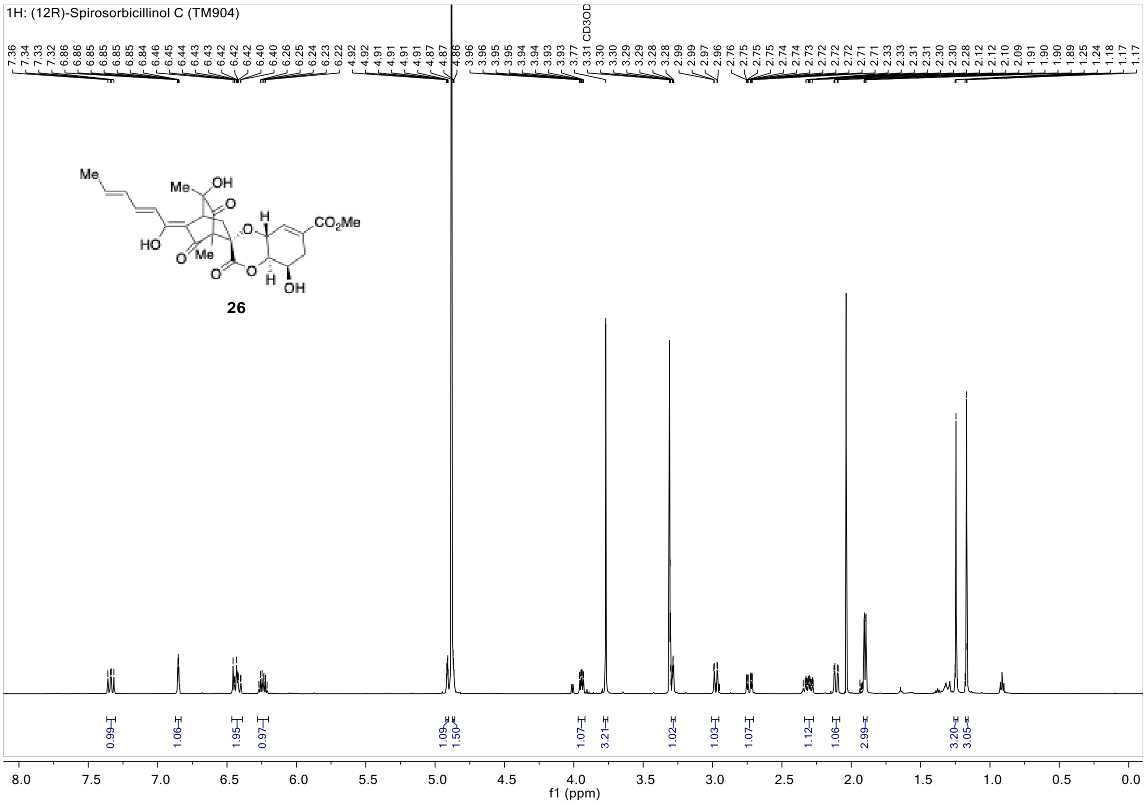


**Fig. S45** $^1$H-NMR spectrum of (12*R*)-spirosorbicillinol C (**26**), measured in $CD_3OD$ at 600 MHz.

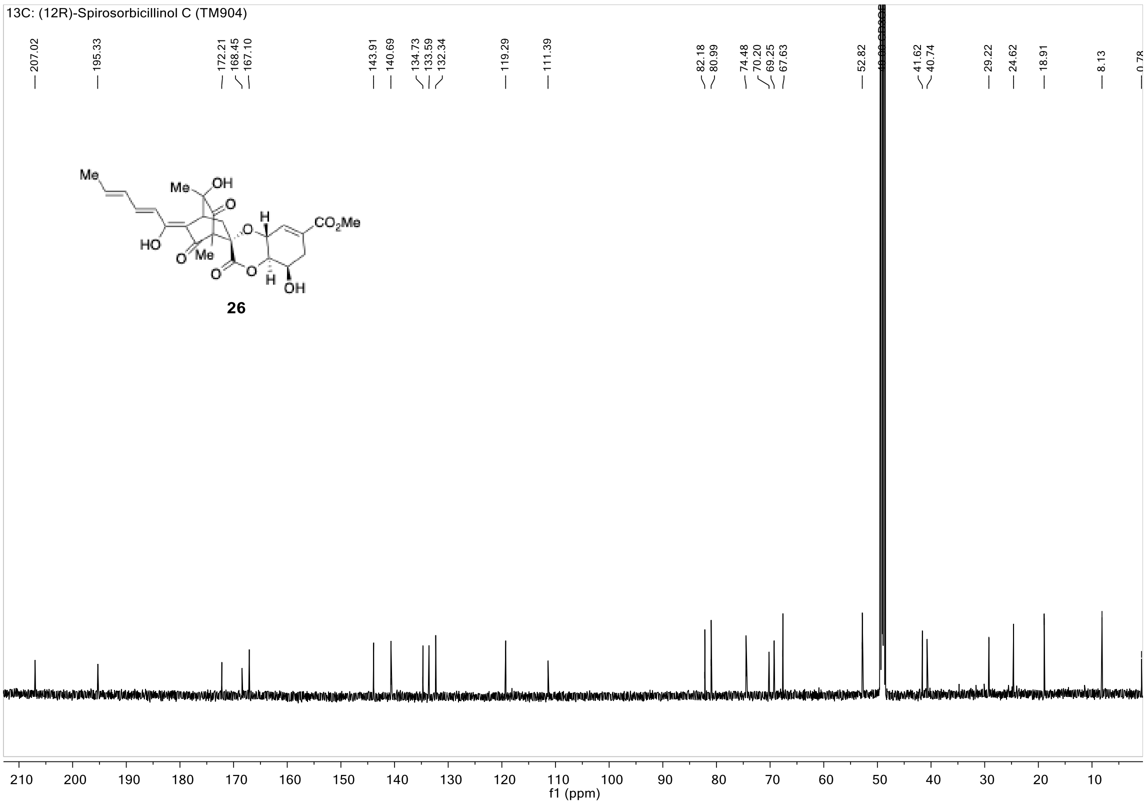


**Fig. S46** $^{13}$C-NMR spectrum of (12*R*)-spirosorbicillinol C (**26**), measured in $CD_3OD$ at 75 MHz.

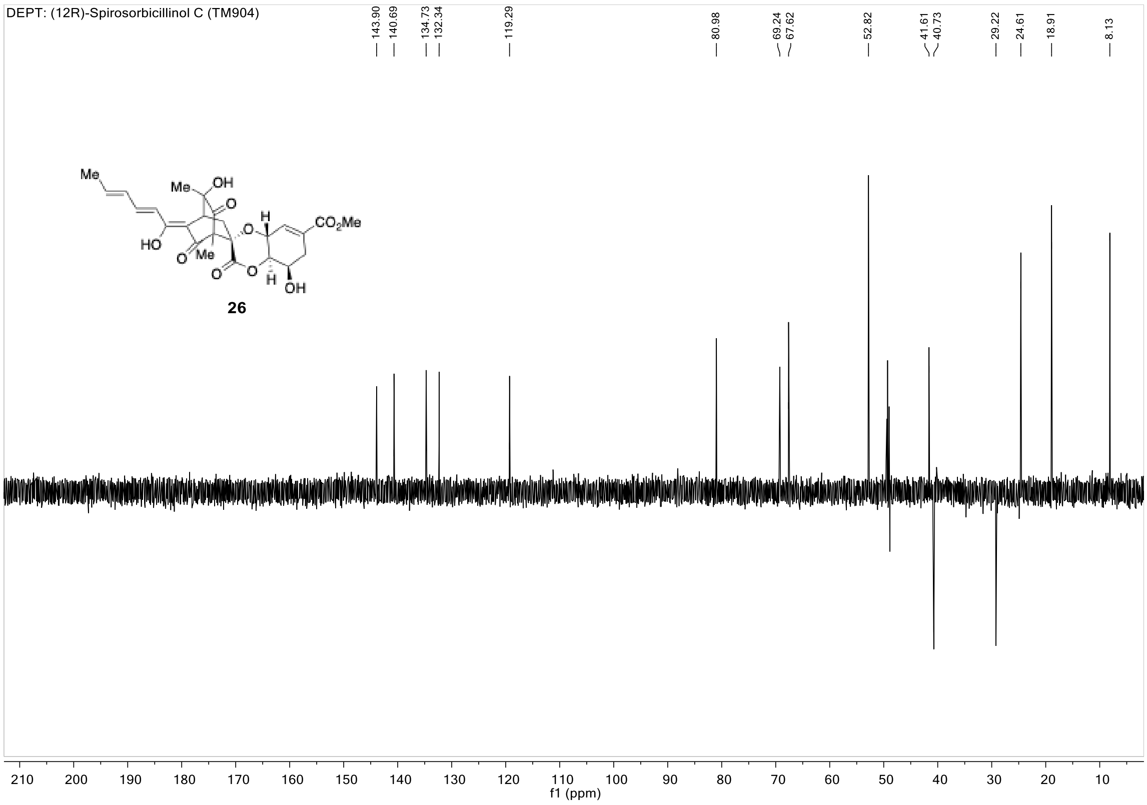


**Fig. S47** DEPT spectrum of (12*R*)-spirosorbicillinol C (**26**), measured in $CD_3OD$ at 75 MHz.

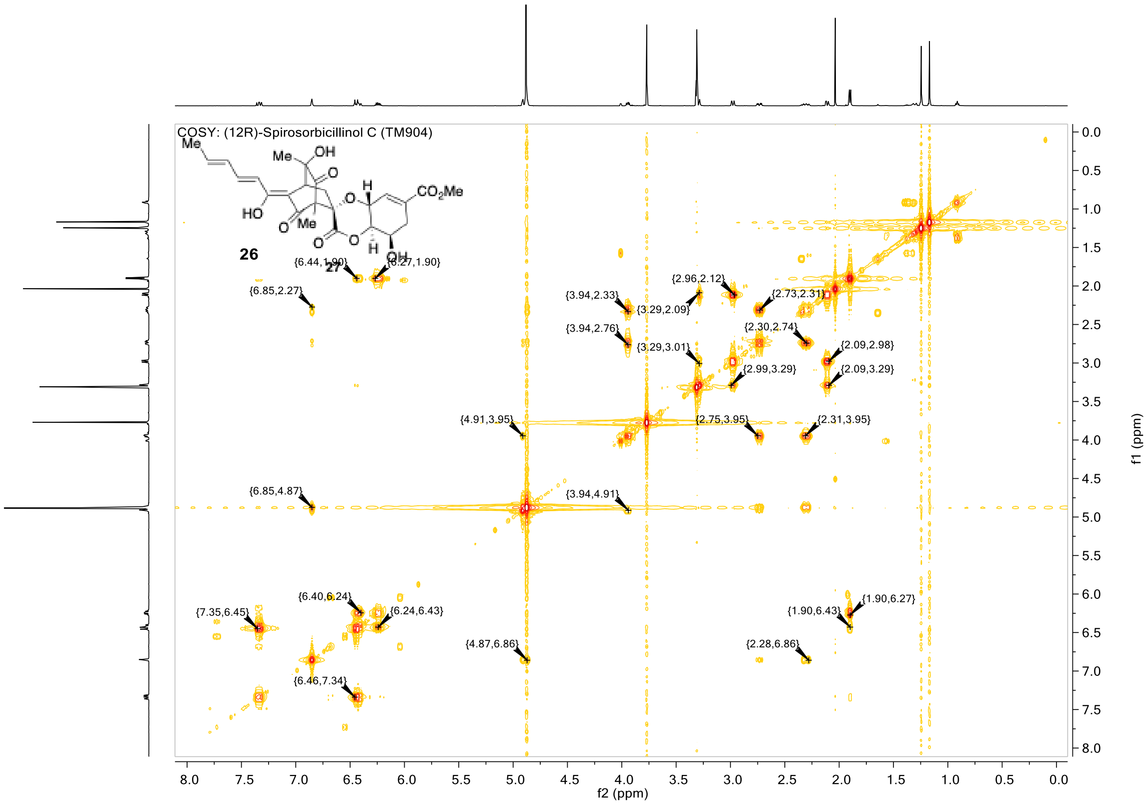


**Fig. S48** COSY spectrum of (12*R*)-spirosorbicillinol C (**26**), measured in $CD_3OD$.

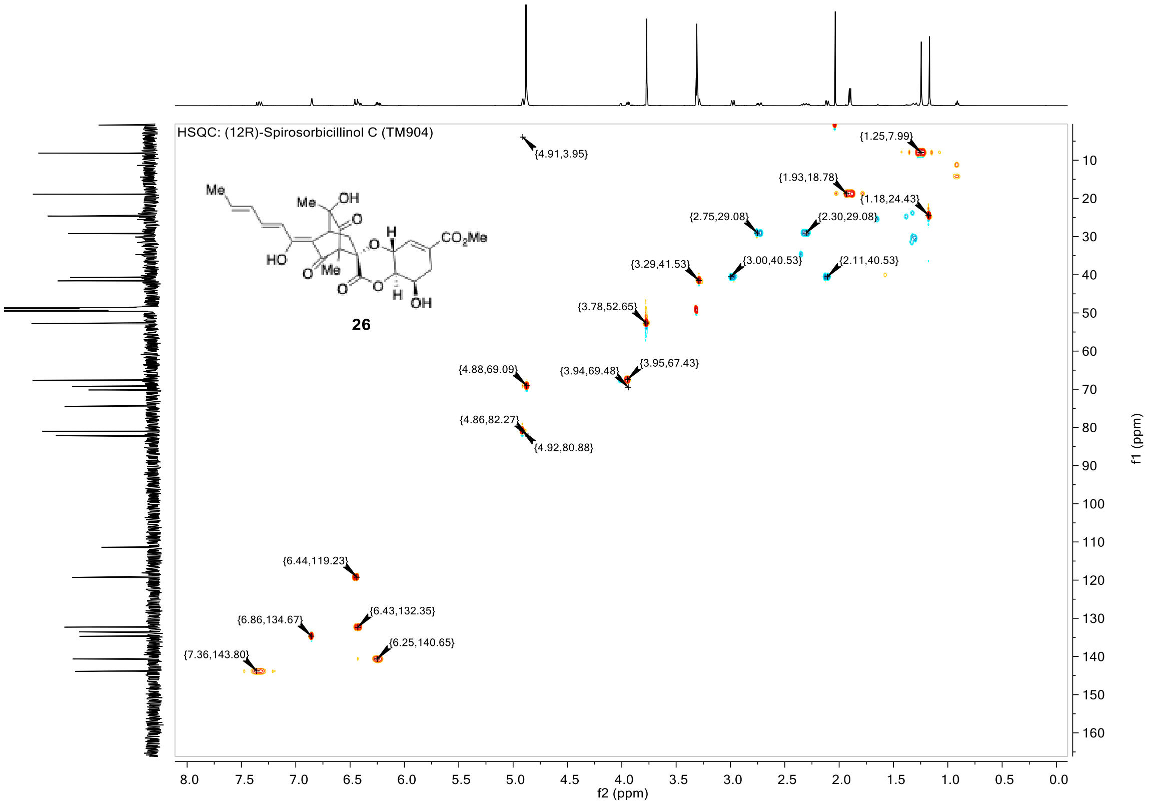


**Fig. S49** HSQC spectrum of (12*R*)-spirosorbicillinol C (**26**), measured in $CD_3OD$.

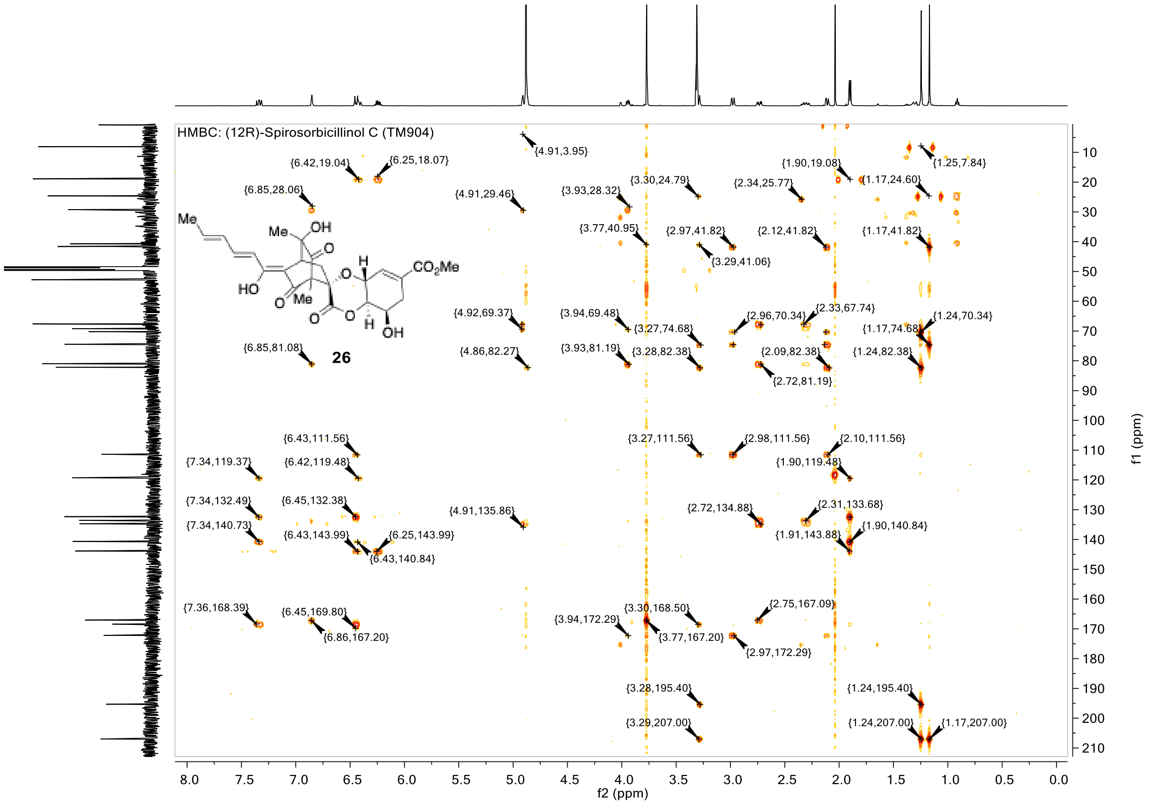


**Fig. S50** HMBC spectrum of (12*R*)-spirosorbicillinol C (**26**), measured in $CD_3OD$.

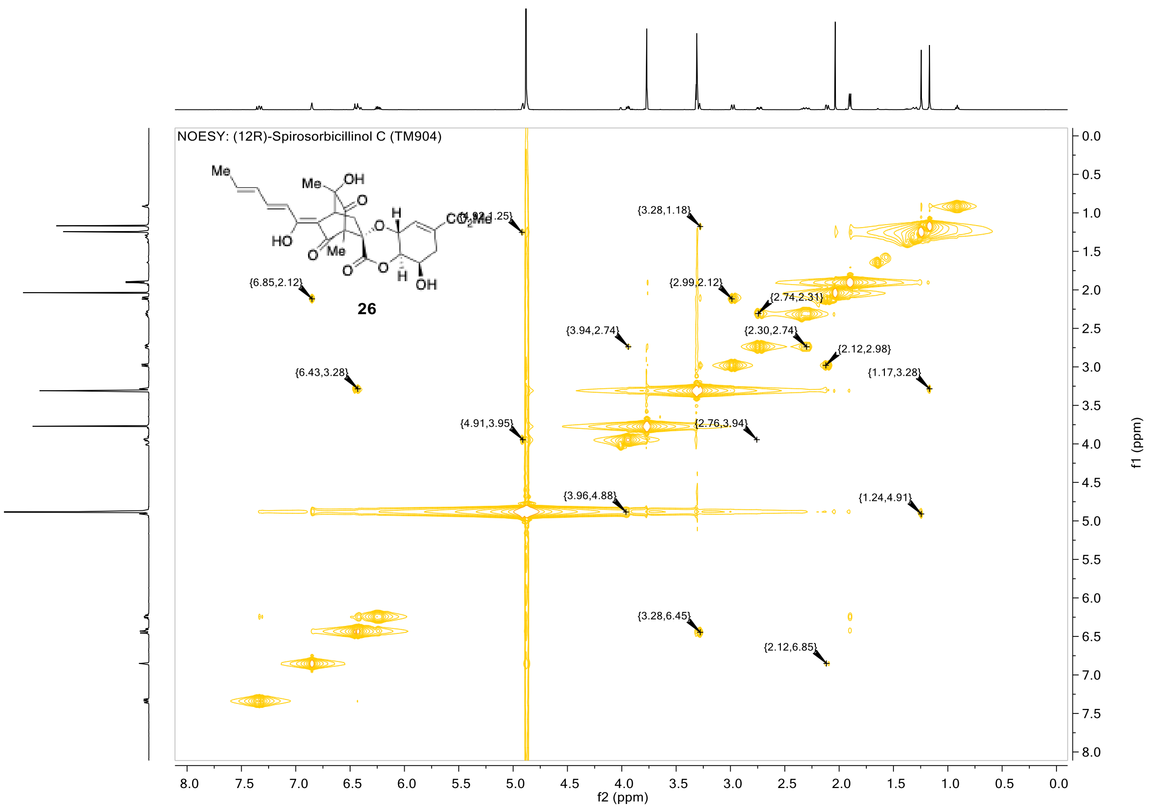


**Fig. S51** NOESY spectrum of (12*R*)-spirosorbicillinol C (**26**), measured in $CD_3OD$.

## 3. Supplementary References


(1) Chahoua, L.; Baltas, M.; Gorrichon, L.; Tisnès, P.; Zedde, C. Synthesis of (–)-Shikimate and (–)-Quinate 3-Phosphates by Differentiation of the Hydroxyl Functions of (–)-Shikimic and (–)-Quinic Acids *J. Org. Chem.* **1992**, 57, 5798–5801.

(2) Chouinard, P. M.; Bartlett, P. A. Conversion of shikimic acid to 5-enolpyruvylshikimate 3-phosphate *J. Org. Chem.* **1986**, 51, 75–78.

(3) Ayer, W. A.; Fukazawa, Y.; & Orszanska, H. Scytolide, A New Shikimate Metabolite from the Fungus *Scytalidium Uredinicol*a *Nat. Prod. Lett.* **1993**, 2, 77–82.

(4) Mazzeo, G.; Santoro, E.; Andolfi, A.; Cimmino, A.; Troselj, P.; Petrovic, A. G.; Superchi, S.; Evidente, A.; Berova, N. Absolute Configurations of Fungal and Plant Metabolites by Chiroptical Methods. ORD, ECD, and VCD Studies on Phyllostin, Scytolide, and Oxysporone *J. Nat. Prod.* **2013**, 76, 588–599.

(5) Banwell, M. G.; Edwards, A. J.; Essers, M.; Jolliffe, K. A. Conversion of (−)-3-Dehydroshikimic Acid into Derivatives of the (+)-Enantiomer *J. Org. Chem.* **2003**, 68, 6839–6841.

(6) Sánchez-Abella, L.; Fernández, S.; Armesto, N.; Ferrero, M.; Gotor, V. Novel and Efficient Syntheses of (−)-Methyl 4-epi-Shikimate and 4,5-Epoxy-Quinic and -Shikimic Acid Derivatives as Key Precursors to Prepare New Analogues *J. Org. Chem.* **2006**, 71, 5396–5399.

(7) Shing, T. K. M.; Tang, Y. (−)-Quinic acid in organic synthesis. 2. Facile syntheses of pseudo-β-D-mannopyranose and pseudo-β-D-fructopyranose *Tetrahedron* **1991**, 47, 4571–4578.

(8) Arnone, A.; Cardillo, R.; Nasini, G.; de Pava, O. V. Cyathiformines A–D, new chorismate-derived metabolites from the fungus clitocybe cyathiformis *Tetrahedron* **1993**, 49, 7251–7258.

(9) Washida, K.; Abe, N.; Sugiyama, Y.; Hirota, A. Novel Secondary Metabolites, Spirosorbicillinols A, B, and C, from a Fungus. *Biosci. Biotechnol. Biochem.* **2009**, *73*, 1355–1361.
